# Design and synthesis of novel 2-(2-(4-bromophenyl)quinolin-4-yl)-1,3,4-oxadiazole derivatives as anticancer and antimicrobial candidates: *in vitro* and *in silico* studies†

**DOI:** 10.1039/d4ra06712f

**Published:** 2024-10-25

**Authors:** Noha Ryad, Ayman Abo Elmaaty, Samy Selim, Mohammed S. Almuhayawi, Soad K. Al Jaouni, Mohamed S. Abdel-Aziz, Arwa Sultan Alqahtani, Islam Zaki, Lina M. A. Abdel Ghany

**Affiliations:** a Pharmaceutical Organic Chemistry Department, College of Pharmaceutical Sciences and Drug Manufacturing, Misr University for Science and Technology 6th of October City, P.O. Box 77 Giza Egypt; b Medicinal Chemistry Department, Faculty of Pharmacy, Port Said University Port Said 42526 Egypt; c Department of Clinical Laboratory Sciences, College of Applied Medical Sciences, Jouf University Sakaka 72388 Saudi Arabia sabdulsalam@ju.edu.sa; d Department of Clinical Microbiology and Immunology, Faculty of Medicine, King Abdulaziz University Jeddah 21589 Saudi Arabia; e Department of Hematology/Oncology, Yousef Abdulatif Jameel Scientific Chair of Prophetic Medicine Application, Faculty of Medicine, King Abdulaziz University Jeddah 21589 Saudi Arabia; f Microbial Chemistry Department, Biotechnology Research Institute, National Research Centre Cairo Egypt; g Department of Chemistry, College of Science, Imam Mohammad Ibn Saud Islamic University (IMSIU) P.O. Box, 90950 Riyadh 11623 Saudi Arabia; h Pharmaceutical Organic Chemistry Department, Faculty of Pharmacy, Port Said University Port Said 42526 Egypt eslam.zaki@pharm.psu.edu.eg; i Pharmaceutical Chemistry Department, College of Pharmaceutical Sciences and Drug Manufacturing, Misr University for Science and Technology 6th of October City, P.O. Box 77 Giza Egypt; j Pharmaceutical Organic Chemistry Department, Clinical Pharmacy Program, East Port Said National University Port Said 42526 Egypt

## Abstract

Cancer is the second leading cause of death globally, surpassed only by heart disease. Moreover, bacterial infections remain a significant global health burden, contributing substantially to morbidity and mortality, especially among hospitalized patients. EGFR has emerged as a prime therapeutic target due to its pivotal role in driving uncontrolled cell growth and survival across numerous cancer types. In addition, DNA gyrase represents a promising target for the development of novel antimicrobial agents. Therefore, we aimed to design and synthesize new multi-target quinoline hybrids (7–17e) capable of acting as anti-proliferative and antimicrobial agents by inhibiting EGFR and microbial DNA gyrase, respectively. The inhibitory potential of the synthesized compounds was determined using *in vitro* and *in silico* approaches. The antiproliferative activity of the synthesized quinoline-oxadiazole derivatives 7–17e was assessed against two cancer cell lines, namely, hepatocellular carcinoma (HepG2) and breast adenocarcinoma (MCF-7). The assessed compounds 7–17e showed considerable cytotoxic activity activities against HepG2 and MCF-7 with IC_50_ values of 0.137–0.332 and 0.164–0.583 μg mL^−1^, respectively, in comparison to erlotinib as the positive control, which showed an IC_50_ value of 0.308 and 0.512 μg mL^−1^, respectively. Moreover, an EGFR tyrosine kinase inhibition assay was conducted on the most prominent candidates. The results showed good IC_50_ values of 0.14 and 0.18 μM for compounds 8c and 12d, respectively, compared to lapatinib (IC_50_ value of 0.12 μM). Furthermore, the minimum antimicrobial inhibitory concentration was evaluated for the most prominent candidates with *S. aureus*, *E. coli*, and *C. albicans*. Compounds 17b, 17d and 17e displayed the most potent inhibitory activity, exhibiting 4-, 16- and 8-fold more activity, respectively, than the reference neomycin. Hence, we can conclude that the afforded compounds can be used as lead dual anticancer and antimicrobial candidates for future optimization.

## Abstract 2

A sequence of novel quinoline-1,3,4-oxadiazole hybrids has been synthesized and evaluated for their *in vitro* anti-proliferative and antimicrobial activities.

## Graphical abstract

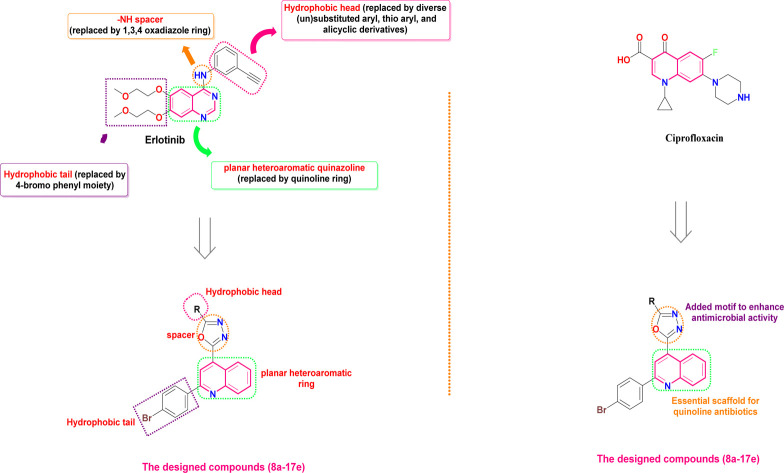

## 1. Introduction

Despite significant advancements in cancer treatment, the disease remains a major global health challenge. The increasing cancer burden is disproportionately affecting lower- and middle-income countries, underscoring the complex interplay between socioeconomic status and health outcomes.^1^ Cancer is the second leading cause of death globally, surpassed only by heart disease. This stark reality underscores the urgent need for continued research and development of effective prevention, diagnostic, and therapeutic strategies.^2^ Furthermore, the limited selectivity of chemotherapeutic agents often results in adverse side effects, including immunosuppression, nausea, anemia, and hair loss. These off-target toxicities highlight the critical challenge of balancing efficacy against cancer cells with minimal harm to healthy tissues.^3^ In response to the urgent need for improved cancer treatments, researchers worldwide are actively pursuing innovative therapeutic strategies. Development of more effective and targeted therapies with reduced side effects remains a primary focus of cancer research.

Protein kinases (PKs) are essential enzymes that regulate a wide range of critical cellular processes, including metabolism, cell growth, survival, and death. Their pivotal role in cellular signaling pathways has made them prime targets for therapeutic interventions, particularly in cancer research.^4^ Protein kinases catalyze the transfer of a phosphate group from ATP to specific hydroxyl residues of amino acids, such as serine, threonine, or tyrosine, on target proteins, a process known as phosphorylation. This crucial phosphorylation process regulates a wide range of cellular functions through intricate signaling networks.^5^ Consequently, aberrant kinase activity, resulting from either hyper activation or mutations, disrupts critical cellular signaling pathways, contributing to the pathogenesis of various diseases, including cancer.^6^

The epidermal growth factor receptor (EGFR) is a prominent protein kinase that plays a pivotal role in regulating cell proliferation and migration.^7^ Many solid tumors, such as non-small cell lung cancer,^8^ hepatocellular carcinoma,^9^ and breast cancer,^4^ overexpress EGFR. Recent advancements in cancer therapy have focused on targeting specific molecules that regulate cancer cell growth and survival.^10,11^ Consequently, EGFR has emerged as a prime therapeutic target due to its pivotal role in driving uncontrolled cell growth and survival across numerous cancer types.^12–17^

**Fig. 1:**
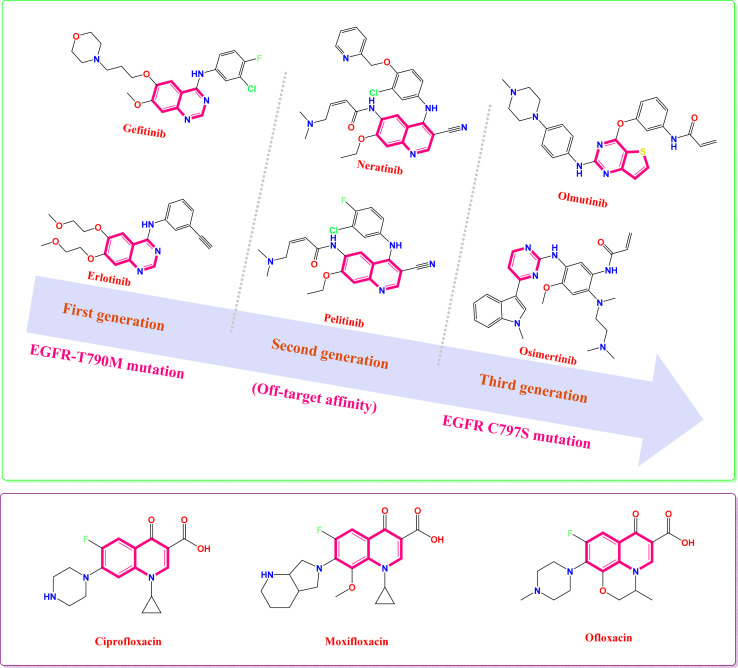
Some FDA-approved quinoline antibiotics and EGFR TK inhibitors, with their different generations as well as their drawbacks.

Erlotinib and gefitinib are examples of first-generation EGFR tyrosine kinase inhibitors.^18–21^ However, their efficacy can be compromised by the development of resistance mechanisms, such as the EGFR-T790M mutation, which diminishes their anticancer potency.^22^ To address the emergence of resistance associated with first-generation EGFR tyrosine kinase inhibitors, second-generation EGFR tyrosine kinase inhibitors (*e.g.*, pelitinib and neratinib) were developed.^23–28^ These drugs have equal affinities towards the wild-type EGFR (WT) and mutant EGFR (EGFR-T790M), resulting in rash and diarrhea.^29^ Hence, the maximal tolerated dose (MTD) displayed by these drugs^30,31^ has led to the emergence of third-generation irreversible EGFR-tyrosine kinase inhibitors (*e.g.*, osimertinib and olmutinib),^32–35^ as shown in Fig. 1. Recently, fourth-generation EGFR tyrosine kinase inhibitors (TKIs) have emerged as a novel therapeutic strategy to address the challenge of acquired resistance mediated by the EGFR C797S mutation, and were subjected to further clinical evaluations.^36^ Fourth-generation EGFR TKIs offer a novel approach to overcoming resistance to EGFR inhibitors by targeting a distinct binding site on the receptor (allosteric inhibitors). This allosteric mechanism of action differentiates them from previous generations of ATP-competitive inhibitors.^36^ The continuous emergence of resistance mechanisms underscores the urgent need for innovative strategies to develop novel EGFR inhibitors with enhanced efficacy and safety profiles.

However, bacterial infections remain a significant global health burden, contributing substantially to morbidity and mortality, especially among hospitalized patients.^37^ Despite the availability of numerous antimicrobial agents, their effectiveness is often compromised by the emergence of bacterial resistance, limiting their clinical utility.^38^ Antibiotic resistance is a pressing global health crisis, contributing to an estimated 700 000 deaths annually.^39^ Without significant advancements in antimicrobial strategies, drug-resistant infections are projected to claim an estimated 10 million lives annually by 2050.^39^ Hence, to address the growing problem of bacterial infections, scientists are desperately searching for new antibiotics that can effectively fight both common and antibiotic-resistant bacteria. These new agents have attracted significant interest in medicinal chemistry research and offer a promising solution to the urgent need for more effective treatments.^37^ Bacterial DNA gyrase, an essential type II topoisomerase, plays a critical role in DNA replication and transcription by introducing negative supercoils into DNA.^40,41^ Given its pivotal role in bacterial survival, DNA gyrase represents a promising target for the development of novel antimicrobial agents. Quinolines (*e.g.*, ciprofloxacin, moxifloxacin, and ofloxacin) have been identified as potent inhibitors of DNA gyrase (Fig. 1). By targeting this essential enzyme, quinolines can effectively disrupt bacterial DNA replication and recombination, leading to cell death.^42^

Furthermore, quinoline and oxadiazole, privileged scaffolds in medicinal chemistry,^43^ have been extensively investigated for its diverse biological properties in numerous research endeavors exhibiting a wide range of pharmacological activities, making them a versatile scaffold for drug discovery, including anticancer,^44–49^ anti-viral,^50–52^ anti-microbial,^53–57^ anti-diabetic,^58–60^ and anti-inflammatory activities.^61–63^ In particular, the literature revealed that quinoline oxadiazole hybrids were utilized as antimicrobial and/or anti-proliferative agents.^42,64,65^

### 1.1. Design rationale

**Fig. 2:**
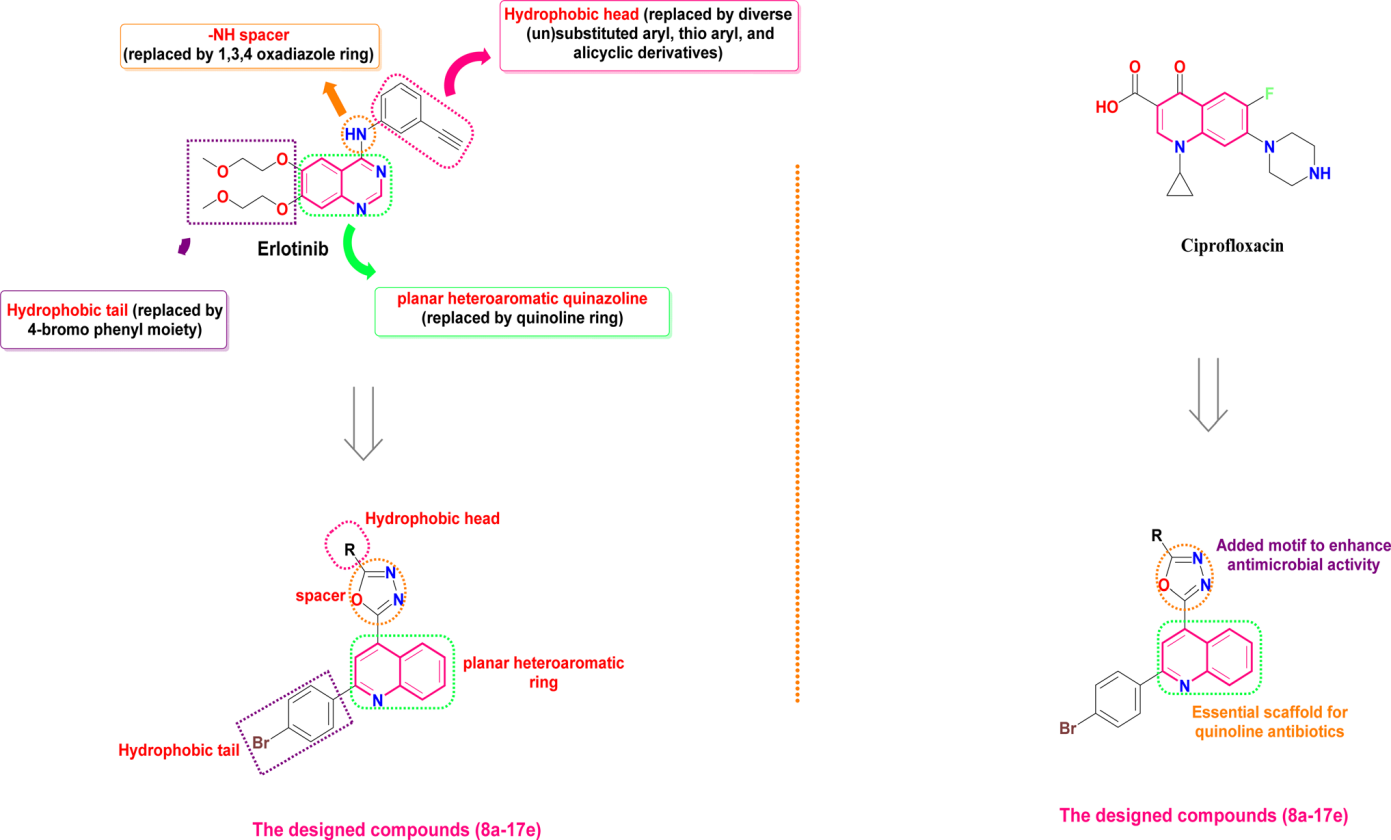
The design rationale of the synthesized compounds (7a–17e) as antiproliferative and antimicrobial agents.

The EGFR-TK pocket, where ATP binds, comprises five main key regions: (a) the adenine binding site, responsible for hydrogen bonding with the ATP adenine moiety; (b) the hydrophilic sugar binding region; (c) hydrophobic region I, critical for inhibitor selectivity; (d) hydrophobic region II, contributing to inhibitor specificity; and (e) the phosphate binding region, which influences inhibitor pharmacokinetics.^66^ A comprehensive understanding of the EGFR-TK binding pocket's structural features is essential for the rational design of potent and selective EGFR inhibitors.^7^ EGFR-TK inhibitors like erlotinib share specific structural features that allow them to bind effectively to the EGFR-TK enzyme. These features include a hydrophobic head fitting into hydrophobic region I, a –NH spacer, a flat heteroaromatic ring system fitting into the adenine binding site and composing hydrogen bonds with amino acids Thr854, Met793, and Thr790, and a hydrophobic tail fitting into the hydrophobic region II. These common pharmacophores enable these inhibitors to interact effectively with EGFR-TK to block its activity.^7^ Herein, *via* application of a molecular hybridization approach to attain all crucial pharmacophoric features, the hydrophobic head of erlotinib was replaced by diverse (un)substituted aryl, thio aryl, alicyclic derivatives for SAR studies. Moreover, the –NH spacer of erlotinib was replaced by a 1,3,4-oxadiazole ring, whereas the flat heteroaromatic quinazoline ring was replaced by the quinoline ring (Fig. 2). In addition, the hydrophobic tail of erlotinib was replaced by a 4-bromo phenyl moiety to afford the designed compounds (8a–17e). However, previous studies have demonstrated the promising antimicrobial potential of 2-phenylquinoline derivatives, highlighting their potential as scaffolds for novel antibacterial agents.^42^ Additionally, attaching the 2-phenyl quinoline scaffold with 1,3,4-oxadiazole motifs could afford molecular hybrids with broad-spectrum antimicrobial activity^42^ (Fig. 2). Hence, in this work, we hoped to design and synthesize a new set of 2-(2-phenylquinolin-4-yl)-1,3,4-oxadiazole hybrids that would act as both anticancer agents (targeting EGFR-TK), as well as antimicrobial agents. The synthesized quinoline-oxadiazole molecules were pursued using *in vitro* and *in silico* approaches for their anti-proliferative and antimicrobial properties.

## 2. Results and discussion

### 2.1. Chemistry

**Scheme 1:**
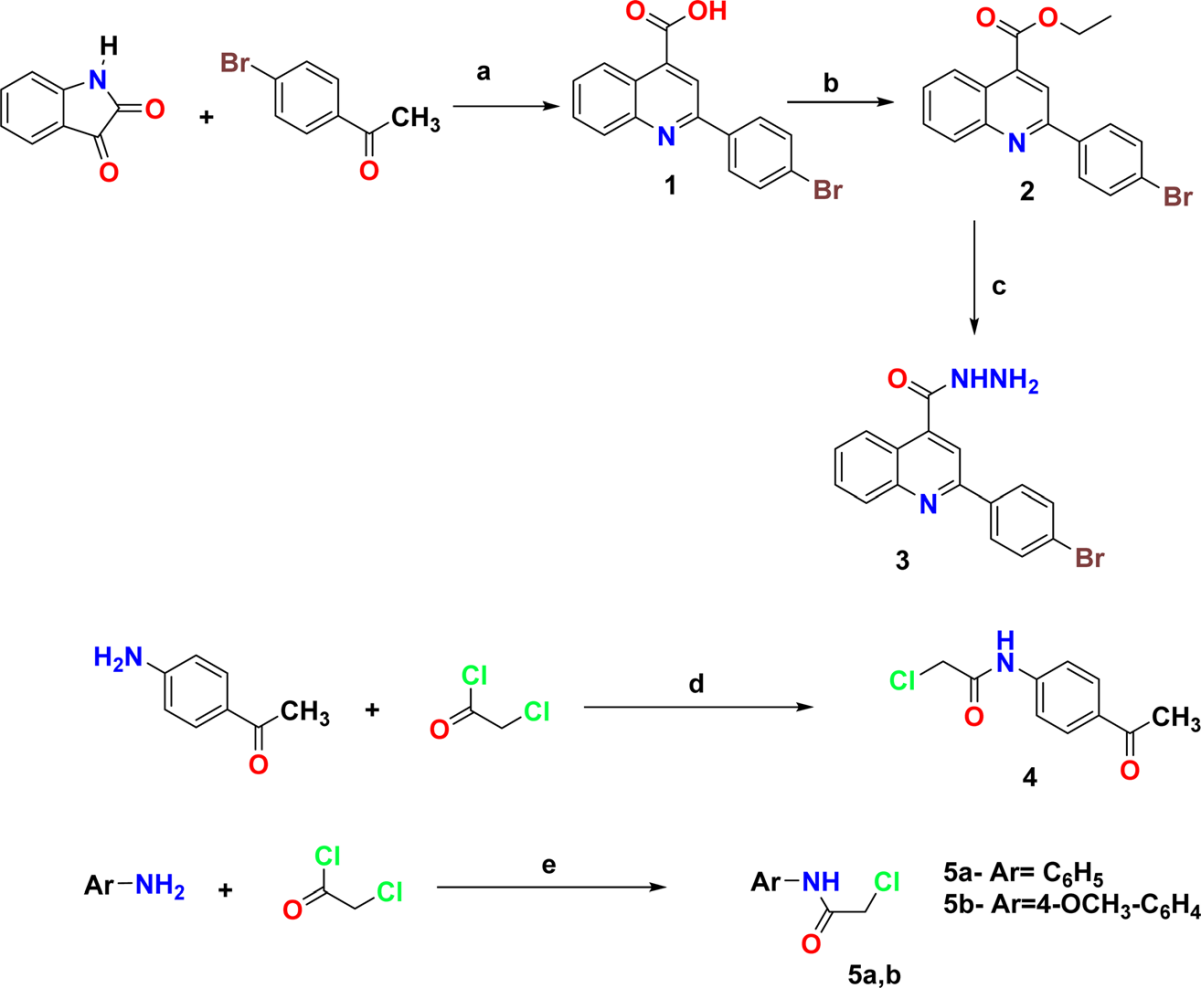
Synthesis of the starting compounds. Reagents and condition: (a) 33% KOH, 96% EtOH, reflux 12 h; (b) absolute EtOH, Conc. H_2_SO_4_, reflux 12 h; (c) NH_2_NH_2_·H_2_O, absolute EtOH, reflux 7 h; (d) CH_2_Cl_2_, TEA, 12, r.t.; (e) glacial AcOH, NaOAc, stirring overnight at r.t.

**Scheme 2:**
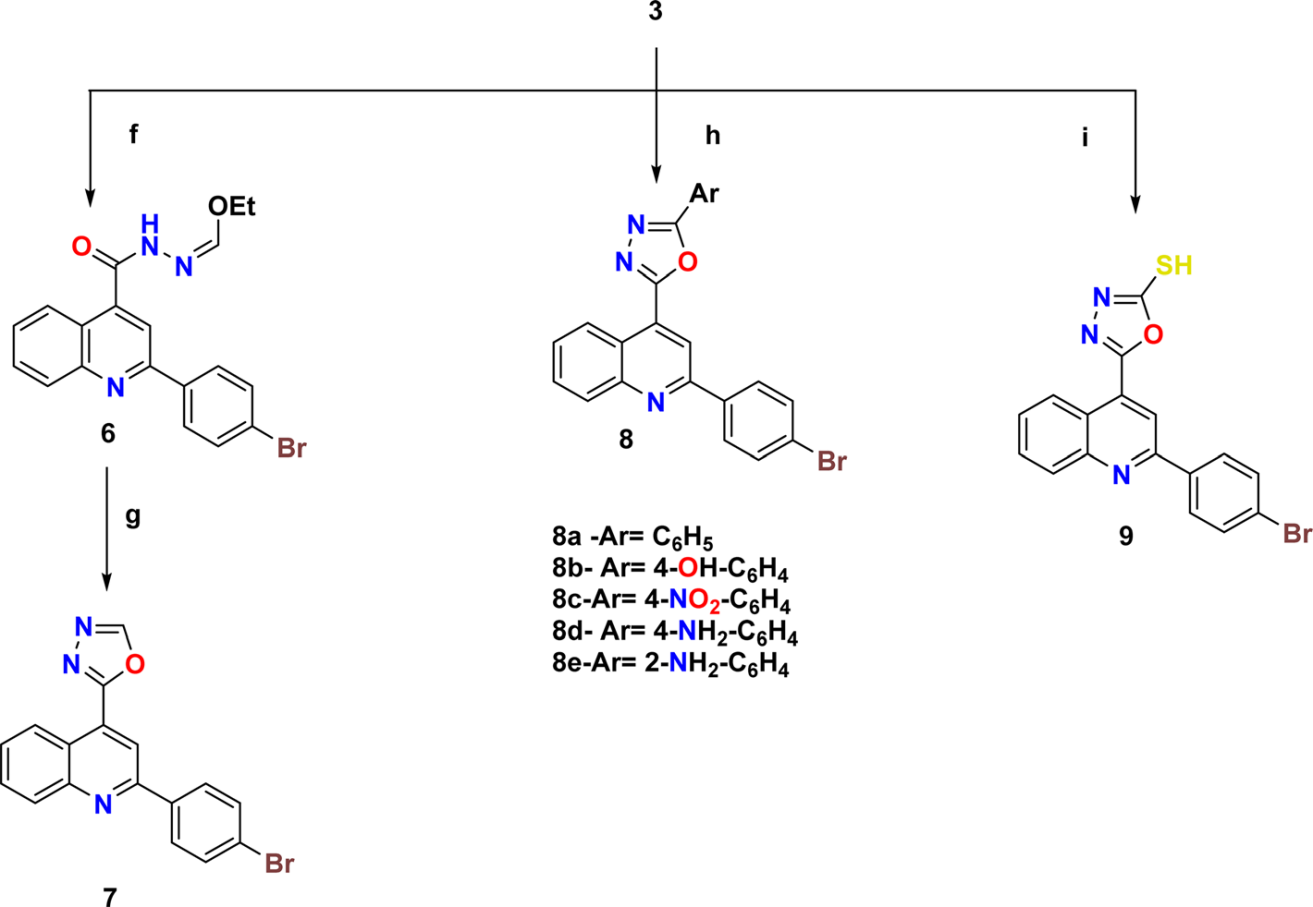
Synthetic pathway for compounds 7–9. Reagents and condition: (f) triethyl orthoformate, reflux 6 h; (g) fusion 30 min; (h) carboxylic acid derivatives, POCl_3_, reflux 6–8 h; (i) KOH, CS_2_, absolute EtOH, reflux, 12 h.

**Scheme 3:**
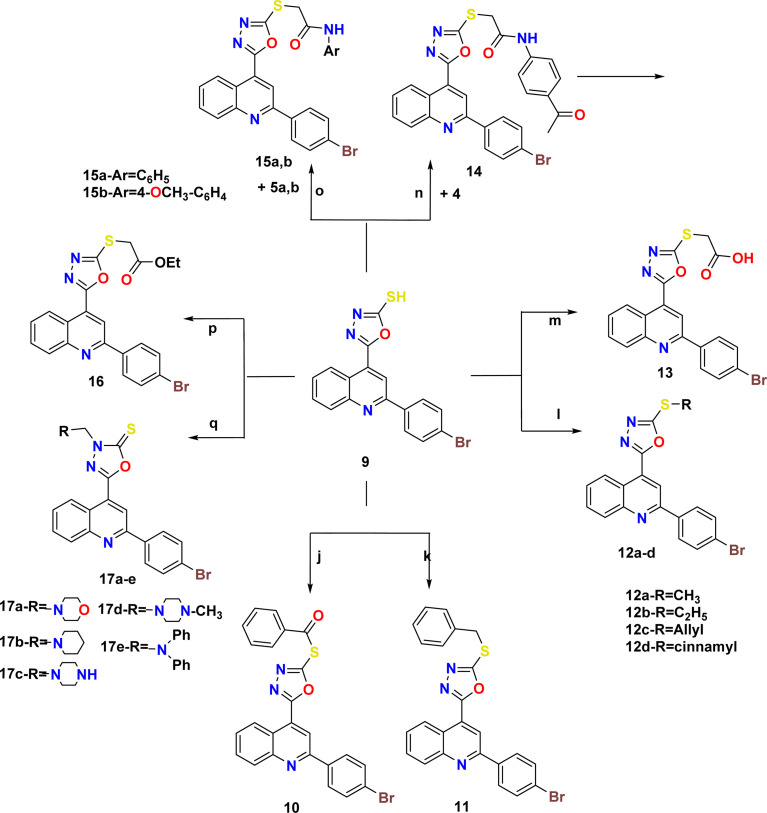
Synthetic pathway for compounds 10–17e. Reagents and conditions: (j) benzoyl chloride, dioxane, stirring overnight; (k) benzyl chloride, anhydrous K_2_CO_3_, acetone, reflux 6 h; (l) alkyl halide derivatives, KOH, aqueous EtOH, reflux 50–60 °C, 4–6 h; (m) chloroacetic acid, TEA, CH_2_Cl_2_, reflux 6 h; (n) TEA, CH_2_Cl_2_, reflux 6 h; (o) KOH, aqueous EtOH, reflux 6–8 h; (p) ethyl chloroacetate, anhydrous K_2_CO_3_, acetone, reflux 5 h; (q) 2^ry^ amine derivatives, KOH, absolute EtOH, 36% HCHO reflux 4–6 h.

The synthetic pathways 2 and 3 were used to create the target products 7–17a–e, with Scheme 1 showing the synthesis of the starting compounds. In Scheme 2, the acid hydrazide 3 is regarded as the important intermediate. Treatment of compound 3 with triethyl orthoformate afforded compound 6,^67^ which was cyclized upon heating in an oil bath at 10 °C above its melting point to give 2-(2-(4-bromophenyl)quinolin-4-yl)-1,3,4-oxadiazole (7) in 61% isolated yield. The IR spectrum of the obtained compound showed the disappearance of the NH and C=O stretching bands. The ^1^H NMR spectrum revealed the characteristic singlet signal at *δ* 9.57 ppm assigned to the proton of the oxadiazole ring. The ^13^C NMR spectrum showed the disappearance of signals corresponding to the ethoxy group, and appearance of two signals at *δ* 154.76 and 167.19 ppm that refer to C5 and C2 of the oxadiazole moiety, respectively. When the acid hydrazide 3 was reacted with appropriate aromatic carboxylic acid derivatives in the presence of phosphorous oxychloride, 5-substituted phenyl-1,3,4-oxadiazoles 8a–e were formed in 62–69% yield. The prepared target molecules were identified by ^1^H NMR spectra, which revealed the fading of two signals of NH and NH_2_ present in the spectra of the parent hydrazide 3, and appearance of signals corresponding to the protons of the added aromatic rings at the expected chemical shift. Heating the acid hydrazide 3 with carbon disulfide in an ethanolic solution of potassium hydroxide resulted in the formation of the 1,3,4-oxadiazole-2-thiol derivative 9 in 83% yield. The IR spectrum of the obtained compound showed the presence of the NH stretching band at 3155 cm^−1^ and a band at 1238 cm^−1^ corresponding to C=S, while the ^1^H NMR spectrum showed singlet signals at *δ* 11.13 ppm that corresponded to the exchangeable SH proton. Furthermore, Scheme 3 describes how new molecules 10–17a–e were created using compound 9 as a crucial intermediate. *S*-(5-(2-(4-Bromophenyl)quinolin-4-yl)-1,3,4-oxadiazol-2-yl) benzothioate (10) was prepared in good yield by stirring oxadiazole-2-thiol 9 with benzoyl chloride in dioxane. The IR spectrum of the synthesized compound showed the absence of an absorption band corresponding to the NH group, and the presence of an absorption band at 1707 cm^−1^ that referred to the C=O group. The ^1^H NMR spectrum exhibited the presence of an additional 5 aromatic protons and the absence of the SH proton, thereby indicating the presence of the benzoyl moiety. The ^13^C NMR spectrum detected the appearance of a signal referring to the C=O group at *δ* 178.86 ppm as well, indicating the elevated number of aromatic carbons of the benzoyl moiety. Heating compound 9 with benzyl chloride in acetone containing K_2_CO_3_ afforded compound 11 in 70% yield. The ^1^H NMR spectrum exhibited the presence of a singlet signal at *δ* 4.62 ppm due to the CH_2_ protons of the benzyl moiety, in addition to signals of the aromatic protons. On the other hand, the ^13^C NMR spectrum showed a CH_2_ signal at *δ* 33.16 ppm, along with signals of the aromatic carbons. 2-(2-(4-Bromophenyl)quinolin-4-yl)-5-(substituted thio)-1,3,4-oxadiazole (12a–d) were prepared in 69–73% yield *via* alkylation of compound 9 with different alkyl halides in ethanol and KOH. The structures of the synthesized compounds were elucidated by ^1^H NMR and ^13^C NMR spectra. The ^1^H NMR spectra showed the disappearance of the signal corresponding to the SH group, and the appearance of a singlet signal at *δ* 2.87 ppm referring to the CH_3_ group in compound 12a. Meanwhile, compound 12b showed triplet and quartet signals at *δ* 1.49 and 3.31–3.44 ppm, which correspond to the CH_3_ and CH_2_- groups, respectively. Furthermore, the allyl group in compound 12c appeared as four signals, a doublet signal at *δ* 4.10 ppm that corresponds to the S-CH_2_ protons, two doublet signals of CH=C̲H_2_ protons at *δ* 5.24 and 5.43 ppm, in which *J* = 10 Hz and 17.2 Hz correspond to cis and trans protons, respectively, due to the vicinal coupling with these non-equivalent protons, and a multiplet signal referring to the C̲H=CH_2_ protons at *δ* 6.00–6.13 ppm. However, compound 12d was confirmed through the appearance of a doublet signal at *δ* 4.28 ppm due to S-CH_2_ protons, a multiplet signal at *δ* 6.50–6.54 ppm due to the =C̲H–CH_2_ proton, doublet signal at *δ* 6.80 ppm due to =C̲H-Ph, and the protons of the phenyl group appeared at *δ* 7.23–8.43 ppm. In the ^13^C NMR spectra, the signal of CH_3_ in compound 12a appeared at *δ* 14.92 ppm. In contrast, compound 12b displayed peaks at *δ* 14.87 and 27.80 ppm due to the CH_3_–CH_2_-group. Meanwhile, compound 12c displayed signals at *δ* 34.90 ppm due to the SCH_2_ carbon and two signals at 118.10 and 133.07 ppm due to CH_2_=CH carbons, respectively. Lastly, the presence of signals at *δ* 35.35, 124.93 and 134.46 pointing to SCH_2_, CH=CH-Ph, respectively, together with the carbons of the phenyl moiety, elucidated the structure of compound 12d. Likewise, heating compound 9 with 2-chloroacetic acid in methylene chloride containing a few drops of TEA led to the formation of compound 13. Moreover, the IR spectrum of the attained compound showed the appearance of the OH and C=O stretching bands of the carboxylic group at 3419 and 1716 cm^−1^, respectively. The ^1^H NMR spectrum revealed characteristic singlet signals at *δ* 4.19 and 5.72 ppm assigned to CH_2_ protons and the exchangeable proton of OH, respectively. The ^13^C NMR spectrum showed two signals at *δ* 36.62 and 169.00 ppm that refer to CH_2_ and C=O, respectively. Also, *N*-(4-acetylphenyl)-2-((5-(2-(4-bromophenyl)quinolin-4-yl)-1,3,4-oxadiazol-2-yl)thio)acetamide (14) was prepared in 86% yield *via* reaction of compound 9 with *N*-(4-acetylphenyl)-2-chloroacetamide (4). Two stretching bands at 3446 and 1670 cm^−1^ emerged in the IR spectrum of compound 14 pertaining to the NH and C=O groups, respectively. The ^1^H NMR spectrum detected distinct singlet signals at *δ* 2.57 and 4.51 ppm assigned to COCH_3_ and CH_2_ protons, respectively, as well as an exchangeable NH proton at *δ* 10.90 ppm. Similarly, heating 1,3,4-oxadiazole 9 with the corresponding acetamide derivative 5a,b afforded 2-((5-(2-(4-bromophenyl)quinolin-4-yl)-1,3,4-oxadiazol-2-yl)thio)-*N*-arylacetamide (15a,b) in 50–55% yield. The structure of the synthesized compounds was verified by IR spectra, which showed the appearance of stretching bands from the NH and C=O groups at 3429–3446 cm^−1^ and 1651–1672 cm^−1^, respectively. The ^1^H NMR spectrum of compound 15b revealed a singlet signal at *δ* 4.24–4.27 ppm assigned to CH_2_ protons, a singlet signal at *δ* 10.75–10.96 ppm pointing to the exchangeable NH proton, and a singlet signal at *δ* 3.83 ppm corresponding to the OCH_3_ protons. The ^13^C NMR spectrum of compound 15b displayed two signals at *δ* 33.63–34.00 and 166.67–168.03 ppm that correspond to CH_2_ and C=O, respectively, and a signal at *δ* 56.06 belonging to OCH_3_. The ester derivative 16 was prepared in 87% yield by heating compound 9 under reflux with ethyl chloroacetate and anhydrous potassium carbonate in dry acetone. The IR spectrum of the obtained compound showed a stretching band of C=O groups at 1739 cm^−1^. Meanwhile, the ^1^H NMR spectrum showed signals at *δ* 1.20 (triplet) and 4.17–4.22 ppm (quartet), corresponding to the CH_3_- and CH_2_-groups, respectively, and a singlet signal at *δ* 4.41 ppm that was assigned to CH_2_ protons. The ^13^C NMR spectrum showed signals for CH_3_, CH_2_, and OCH_2_ at *δ* 14.14 ppm, 32.14 ppm, and 62.29 ppm, respectively. Finally, a new series of Mannich bases from the 1,3,4-oxadiazole derivative was synthesized in 74–82% yield by reacting the 1,3,4-oxadiazole derivative 9 with formaldehyde and an appropriate secondary amine (morpholine, piperidine, piperazine, methylpiperazine and diphenyl amine). The structure of the synthesized compounds was demonstrated by ^1^H NMR spectrum, which showed a singlet signal at *δ* 5.14–5.23 ppm corresponding to CH_2_ protons, along with the added protons of either morpholine, piperidine, piperazine, methylpiperazine, or diphenyl moieties at the expected chemical shift. The ^13^C NMR spectrum showed a signal for CH_2_ at *δ* 69.34–83.48 ppm, and the signal of the respective carbons of the prepared compounds was verified on the basis of their chemical shift. The target compound's molecular ion peaks, which matched their calculated molecular weights, also provided more evidence of their structure, along with the elemental analyses of their CHN components.

### 2.2. Biological evaluation

#### 2.2.1. Cytotoxic activity against two cancer cell lines

The antiproliferative activity of the synthesized quinoline-oxadiazole derivatives 7–17e was assessed against two cancer cell lines; namely, the hepatocellular carcinoma (HepG2) and breast adenocarcinoma (MCF-7) cancer cell lines. From the obtained results in Table 1, it was found that the assessed quinoline having the oxadiazole moiety 7–17e showed considerable cytotoxic activity activities against HepG2 and MCF-7 with IC_50_ values of 0.137–0.332 and 0.164–0.583 μg mL^−1^, respectively, in comparison to erlotinib as a positive control, which showed IC_50_ values of 0.308 and 0.512 μg mL^−1^. Regarding the activity against HepG2 liver cancer cells, the quinoline-like oxadiazole compounds 8d, 12b and 13 were the least active molecules with IC_50_ values of 0.311, 0.327 and 0.332 μg mL^−1^, respectively, compared with other quinoline-oxadiazole derivatives (IC_50_ range: 0.137–0.272 μg mL^−1^). The 2-(4-hydroxyphenyl)-1,3,4-oxidiazole in 8b (IC_50_ = 0.139 μg mL^−1^) was equipotent to 2-(4-nitrophenyl)-1,3,4-oxidiazole 8c (IC_50_ = 0.137 μg mL^−1^). The presence of 2-(2-aminophenyl)-1,3,4-oxidiazole 8e (IC_50_ = 0.157 μg mL^−1^) resulted in higher activity than its positional congener 8d (IC_50_ = 0.311 μg mL^−1^) or unsubstituted phenyl derivative 8a (IC_50_ = 0.215 μg mL^−1^). Meanwhile, the methylthio group on the C2 of the 1,3,4-oxadiazole moiety resulted in compound 12a with good cytotoxic activity (IC_50_ = 0.138 μg mL^−1^). Conversely, changing the methylthio group with either ethylthio 12b or allylthio 12c resulted in a decrease of the cytotoxic activity with IC_50_ values of 0.327 and 0.158 μg mL^−1^, respectively. Moreover, replacement of the methylthio group with cinnamyl function resulted in compound 12d with equipotent cytotoxic action (IC_50_ = 0.138 μg mL^−1^). The *N*-(4-acetylphenyl)acetamidethio group in compound 14 (IC_50_ = 0.159 μg mL^−1^) showed equipotent antiproliferative activity against the HepG2 cell line to *N*-(4-methoxyphenyl)acetamidethio having compound 15b (IC_50_ = 0.154 μg mL^−1^), which was superior to the unsubstituted phenyl-bearing compound 15a (IC_50_ = 0.272 μg mL^−1^). The 3-(substituted methyl)-1,3,4-oxadiazole-2(3*H*)-thiones 17a–e showed the best activity in the case of the diphenylamino methyl derivative 17e (IC_50_ = 0.139 μg mL^−1^), followed by the morpholin-4-ylmethyl derivative 17a (IC_50_ = 0.141 μg mL^−1^), the piperazin-1-ylmethyl derivative 17c (IC_50_ = 0.164 μg mL^−1^), the piperidin-1-ylmethy derivative 17b (IC_50_ = 0.188 μg mL^−1^), and the least antiproliferative action was displayed by 4-methylpiperazin-1-ylmethyl molecule 17d (IC_50_ = 0.254 μg mL^−1^). With respect to antiproliferative activity against MCF-7 cells, quinoline compounds 8e incorporating the 2-(2-aminophenyl)-1,3,4-oxadiazole moiety and 15a having the 2-(*N*-phenylacetamidethio)-1,3,4-oxadiazole moiety were the best potent derivatives with IC_50_ values of 0.179 and 0.164 μg mL^−1^, respectively, exceeding the cytotoxic activity of the reference drug erlotinib (IC_50_ = 0.512 μg mL^−1^). Additionally, the majority of the quinoline-1,3,4-oxadiazole derivatives demonstrated considerable cytotoxic activity against the MCF-7 cells with IC_50_ values ranging from 0.217 to 0.583 μg mL^−1^ (Table 1).

**Table 1:** Results of the IC_50_ values of the target quinoline-1,3,4-oxadiazole hybrids 7–17e toward two cancer cell lines

Compd no.	IC_50_ (μg mL^−1^)	
	HepG-2	MCF-7
7	0.151	0.295
8a	0.215	0.440
8b	0.139	0.225
8c	0.137	0.481
8d	0.311	0.287
8e	0.157	0.179
9	0.152	0.358
10	0.217	0.446
11	0.141	0.227
12a	0.138	0.473
12b	0.327	0.287
12c	0.158	0.239
12d	0.138	0.473
13	0.332	0.287
14	0.159	0.217
15a	0.272	0.164
15b	0.154	0.411
17a	0.141	0.300
17b	0.188	0.583
17c	0.164	0.221
17d	0.254	0.406
17e	0.139	0.569
Erlotinib	0.308	0.512

#### 2.2.2. Cell cycle analysis

**Fig. 3:**
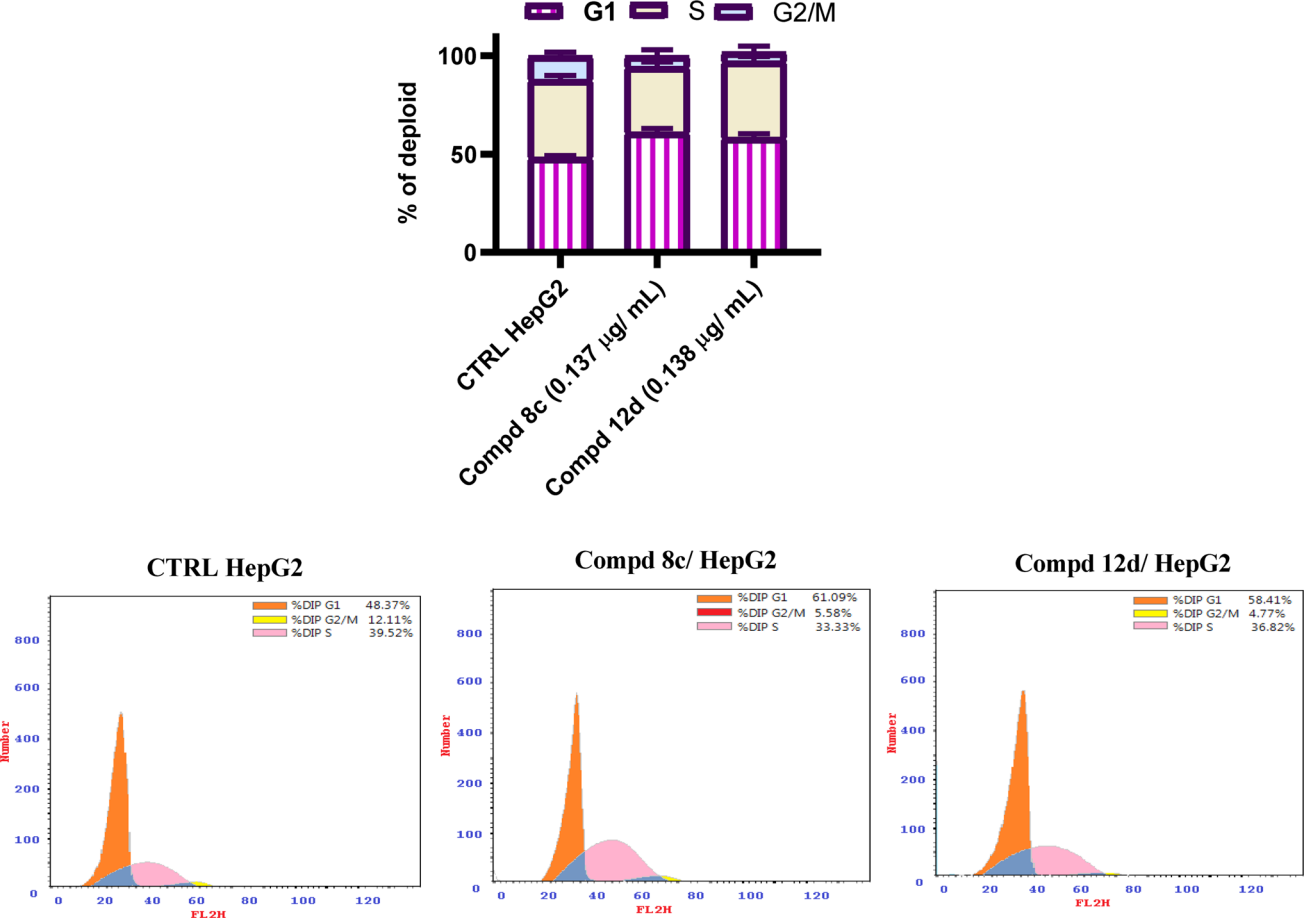
Influence of quinoline molecules 8c and 12d on the cellular cycle proportion following the HepG2 cancerous cell line treatment relative to untreated cells.

To investigate the molecular mechanism of the antiproliferative activity of the best potent quinoline compounds 8c having the 2-(4-nitrophenyl)-1,3,4-oxadiazole moiety and 12d containing the 2-(cinnamylthio)-1,3,4-oxadiazole moiety, the effects of these compounds at their IC_50_ concentration on the cellular cycle progression of HepG2 cancer cells were evaluated by FACS technique.^68^ As displayed in Fig. 3, the treatment of hepatocellular carcinoma HepG2 cells with quinoline compounds 8c having the 2-(4-nitrophenyl)-1,3,4-oxadiazole moiety and 12d containing the 2-(cinnamylthio)-1,3-4-oxadiazole moiety for 48 h induced cellular cycle arrest at the G1 phase. The proportion of HepG2 cells was increased from 48.37% in the untreated group to 61.09% and 58.41% in the cells treated with the examined compounds 8c and 12d, respectively. The results in this assay indicated that quinoline compounds 8c having the 2-(4-nitrophenyl)-1,3,4-oxadiazole moiety and 12d grafted with the 2-(cinnamylthio)-1,3,4-oxadiazole moiety possibly displayed antiproliferative activity through cellular cycle arrest at the G1 phase in hepatocellular carcinoma HepG2 cells.

#### 2.2.3. Apoptosis analysis

**Fig. 4:**
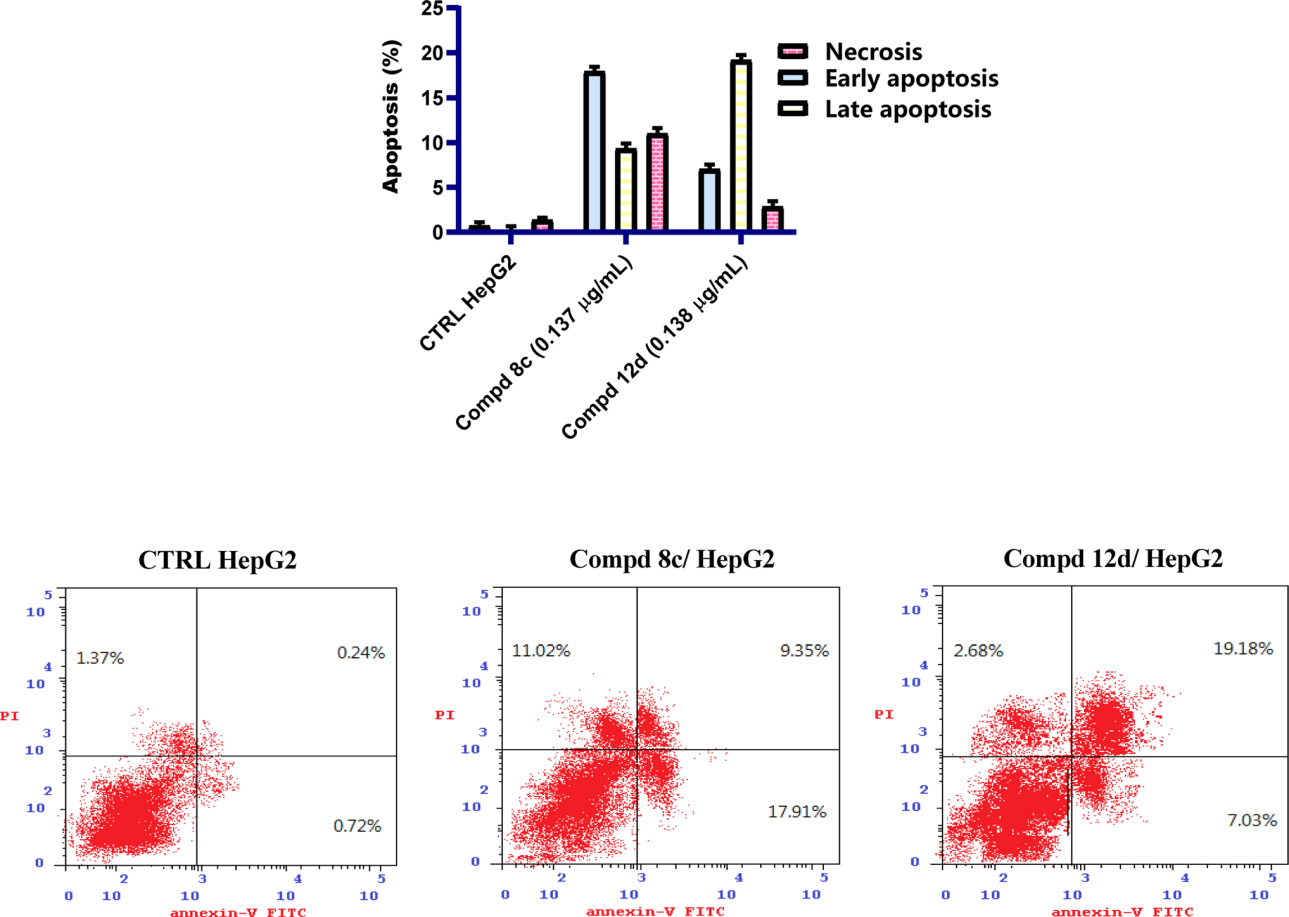
Influence of quinoline compounds 8c and 12d on the apoptosis concentration, following staining with Annexin V/PI in HepG2 cells relative to untreated cells.

The apoptotic effects of quinoline compounds 8c having the 2-(4-nitrophenyl)-1,3,4-oxadiazole moiety and 12d containing the 2-(cinnamylthio)-1,3,4-oxadiazole moiety on HepG2 cells were assessed. Fig. 4 illustrates the results after HepG2 cells were exposed to quinoline compounds 8c and 12d at the IC_50_ concentrations of 0.137 and 0.138 μg mL^−1^, respectively, for 48 h. The percentage of cells that were apoptotic was increased by 28.8- and 27.3-times, respectively. These proportions were greater than that of the control untreated cells, which afforded 0.96%. The percentage of early apoptotic cells of the examined compounds was increased by 24.9- and 9.8-times, respectively. In addition, the late apoptotic percentages after treatment with quinoline compounds 8c and 12d were increased by 38.9- and 79.9-fold, respectively. From the obtained data, it could be concluded that the quinoline derivatives 8c and 12d provoked HepG2 cellular apoptosis.

#### 2.2.4. EGFR tyrosine kinase inhibition assay

**Fig. 5:**
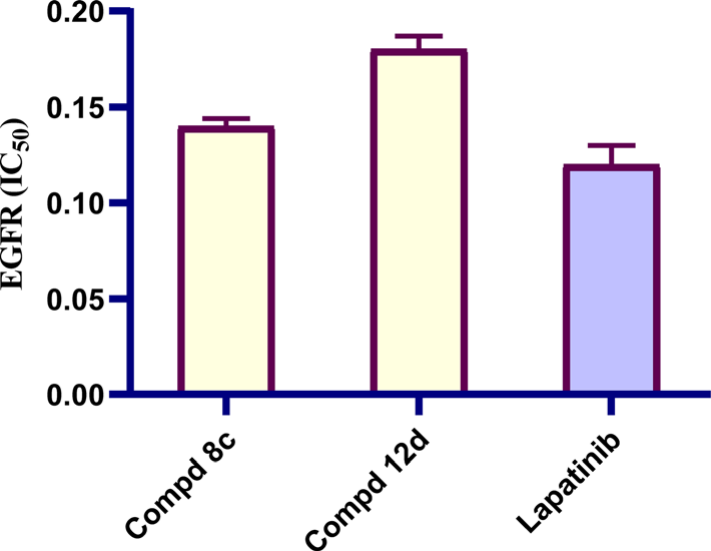
The IC_50_ (μM) of the target quinoline molecules 8c and 12d against EGFR kinase activity compared to lapatinib.

To further explore the antiproliferative effect of the prepared quinoline-1,3,4-oxadiazole derivatives, an additional mechanistic study was conducted by investigating the binding affinity of representative active quinoline compounds 8c having the 2-(4-nitrophenyl)-1,3-4-oxadiazole moiety and 12d containing the 2-(cinnamylthio)-1,3-4-oxadiazole moiety to EGFR-TK using lapatinib as the positive control. The results are shown in Fig. 5, and display good IC_50_ values of 0.14 and 0.18 μM on EGFR for the tested quinoline compounds 8c and 12d, respectively, compared to lapatinib (IC_50_ of 0.12 μM on EGFR). The observed results indicated that quinoline compounds 8c having the 2-(4-nitrophenyl)-1,3-4-oxadiazole moiety and 12d containing the 2-(cinnamylthio)-1,3-4-oxadiazole moiety are favourable for EGFR-TK inhibition.

#### 2.2.5. Effect of quinoline compounds on the expression of apoptotic markers

**Fig. 6:**
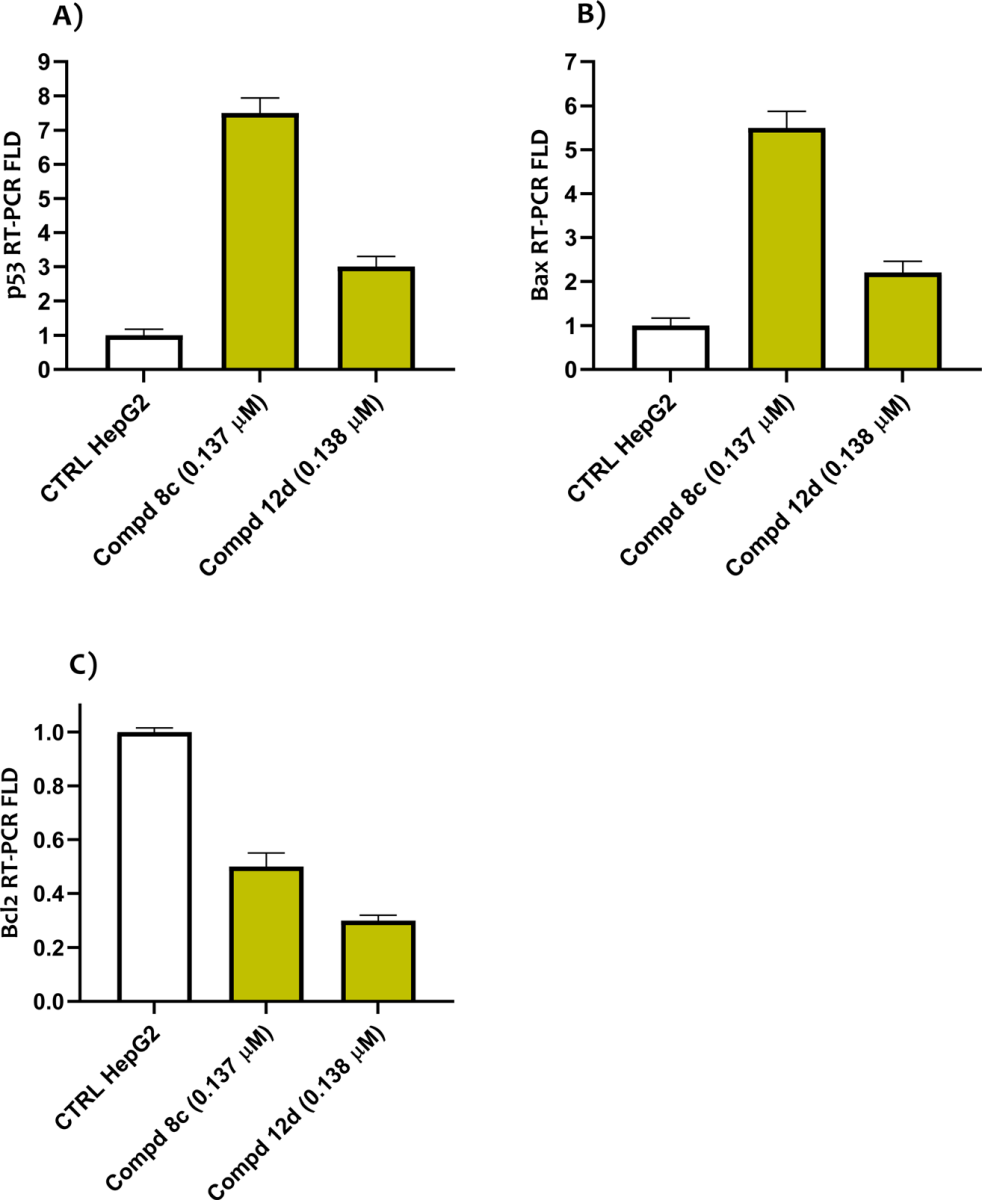
Influence of the quinoline-oxadiazole compounds 8c and 12d on apoptosis-associated proteins after 48 h treatment in HepG2 cells.

Three known tumor suppressor genes, p53, Bax and Bcl2, play a decisive role in the process of cell apoptosis.^69^ Downregulation of the Bcl-2 protein, along with overexpression of p53 and Bax, can cause cellular apoptosis.^70^ To understand the impacts of target quinoline molecules on the cellular death markers-dependent pathway, HepG2 cells were treated with quinoline compounds 8c and 12c at the IC_50_ concentration. A qRT-PCR assay was then used to assess the expression levels of p53, Bax and Bcl-2. When compared to the control untreated cells, it was shown that quinoline molecules 8c and 12c increased the level of p53 in HepG2 cells by 7.5- and 3-fold, respectively. Coordinately, the Bax levels of quinoline compounds 8c and 12c were 3- and 2.2-times more than those of the untreated controls. Simultaneously, the Bcl-2 concentrations were 0.5- and 0.3-lower than those of the untreated group (Fig. 6). According to these findings, the cytotoxicity and death of cancer cells generated by quinoline compounds 8c and 12c may be related to apoptosis.

#### 2.2.6. *In vitro* antimicrobial activity

The *in vitro* antimicrobial activities of the target quinoline-oxadiazole hybrids 7–17e were examined against the microbial strains *S. aureus*, *E. coli*, *C. albicans* and *A. niger*. The reference drugs (*e.g.*, neomycin and cyclohexamide) were utilized to investigate the antimicrobial activity of the examined quinoline-oxadiazole molecules 7–17e. The antimicrobial results are displayed in Table 2, and are presented as the average millimeter diameter of the microbial growth inhibitory zone encircling the disc. The results demonstrated that quinoline-oxadiazole compounds 9–11 and 17a–e displayed the most potent antimicrobial action against *S. aureus*, *E. coli*, and *C. albicans*. However, none of the examined quinoline-oxadiazole compounds were found to be effective against *A. niger* fungal strains. It was noted that compounds 17a–e were found to exhibit considerable antimicrobial action with a zone inhibition value range of 29–37 mm. Remarkably, quinoline-oxadiazole molecules 17a–e exhibited superior antimicrobial activity, surpassing the reference drugs neomycin and cyclohexamide. It was also shown that at 37 mm zone inhibition value, compound 17a was the most effective inhibitor against *S. aureus* and *C. albicans*. Conversely, compound 17d was the most active against *E. coli* (inhibition zone value of 37 mm). Furthermore, compound 10 was more potent against *E. coli* (inhibition zone value of 30 mm) than *S. aureus* (inhibition zone = 25 mm).

**Table 2:** The antimicrobial activity of quinoline-oxadiazole compounds 7–17e, neomycin and cyclohexamide

Comp. no.	Gram+ bacteria	Gram− bacteria	Fungi	
	*S. aureus*	*E. coli*	*C. albicans*	*A. niger*
7	12	14	13	0
8a	0	0	0	0
8b	0	0	0	0
8c	0	0	0	0
8d	0	0	0	0
8e	0	0	0	0
9	20	23	24	0
10	25	30	28	0
11	14	17	14	0
12a	0	0	0	0
12b	0	0	0	0
12c	0	0	0	0
12d	0	0	0	0
13	0	0	0	0
14	0	0	0	0
15a	0	0	0	0
15b	0	0	0	0
16	0	0	0	0
17a	37	35	37	0
17b	33	34	36	0
17c	35	36	35	0
17d	34	37	36	0
17e	29	32	30	0
Neomycin	26	24	30	—
Cycloheximide			0	21

#### 2.2.7. Determination of the minimum inhibitory concentration (MIC)

To find the minimal inhibitory concentration (MIC) of the quinoline-oxadiazole molecules 9–11 and 17a–e, a two-fold serial dilution assay was utilized. Neomycin was employed as the reference antimicrobial drug. Results in Table 3 revealed that all of the tested quinoline compounds demonstrated considerable activity against *S. aureus* with MIC ranges of 4.88–78.125 μg mL^−1^ relative to the reference compound (MIC = 78.125 μg mL^−1^). Compounds 17b, 17d and 17e displayed the most potent inhibitory activity with 4-, 16- and 8-times more potency than the reference neomycin, respectively. In addition, compounds 9, 10 and 17a showed equipotent MIC activity against *S. aureus* (MIC = 39.06 μg mL^−1^), which were 2-times more effective than the reference. Compound 11 showed equipotent MIC activity to the reference molecule neomycin (MIC = 78.125 μg mL^−1^). Regarding the MIC activity against *E. coli*, all of the examined quinoline derivatives 9–11 and 17a–e were less active with the MIC activity range of 312.5–625 μg mL^−1^ compared to neomycin (MIC = 39.06 μg mL^−1^ on *E. coli*). Against *C. albicans*, all of the examined quinoline compounds demonstrated significant MIC activity with MIC ranges of 4.88–39.06 μg mL^−1^ relative to the reference drug neomycin (MIC = 156.25 μg mL^−1^ on the *C. albicans* microbial strain).

**Table 3:** MIC data for the quinoline-oxadiazole derivatives 9–11 and 17a–e against different microbes

Comp. no.	MIC (μg mL^−1^)		
	*S. aureus*	*E. coli*	*C. albicans*
9	39.06	625	19.53
10	39.06	312.5	19.53
11	78.125	625	39.06
17a	39.06	156.25	4.88
17b	19.53	312.5	9.77
17c	78.125	312.5	4.88
17d	4.88	312.5	9.77
17e	9.77	312.5	4.88
Neomycin	78.125	39.06	156.25

#### 2.2.8. Determination of the minimum bactericidal concentration (MBC)

The current work evaluated the MBC inhibitory ability of the quinoline-oxadiazole hybrids 9–11 and 17a–e against the *S. aureus*, *E. coli* and *C. albicans* microbial strains. The results presented in Table 4 indicated that the *S. aureus* and *C. albicans* microbial strains were found to be more sensitive to the tested quinoline compounds 9–11 and 17a–e than the *E. coli* bacterial strain. Regarding *S. aureus*, all examined quinoline compounds displayed equipotent or more potent MBC inhibitory activity (MBC ranges: 19.53–312.5 μg mL^−1^) compared to the reference drug neomycin (MBC = 312.5 μg mL^−1^). 4-Methylpiperazin-1-ylmethyl-1,3,4-oxadiazole in 17d and diphenylamino-methyl-1,3,4-oxadiazole in 17e were the most potent compounds in this study with an equal MBC value of 19.53 μg mL^−1^. Replacement with either morpholin-4-ylmethyl-1,3,4-oxadiazole 17a or piperidin-1-ylmethy-1,3,4-oxadiazole 17b decreased the MBC activity against *S. aureus*. Conversely, the 2-(benzoylthio)-1,3,4-oxadiazole derivative 10 revealed higher activity than the 2-(benzylthio)-1,3,4-oxadiazole derivative 11. For *E. coli*, all of the tested quinoline derivatives 9–11 and 17a–e were less active with the MBC inhibition range of 312.5–1250 μg mL^−1^ relative to the reference drug neomycin (MBC = 156.5 μg mL^−1^ on the *E. coli* strain). Furthermore, the most potent quinoline derivative against *C. albicans* was morpholin-4-ylmethyl-1,3,4-oxadiazole 17a (MBC = 4.88 μg mL^−1^) compared to the reference drug neomycin (MBC = 625 μg mL^−1^). In addition, compounds 17b, 17c and 17e showed an equipotent MBC value of 9.77 μg mL^−1^, which was superior to that of the reference drug neomycin (MBC = 156.5 μg mL^−1^). It should also be noted that the 2-(benzoylthio)-1,3,4-oxadiazole derivative 10 (MBC = 19.53 μg mL^−1^) demonstrated higher MBC inhibitory activity than the 2-(benzylthio)-1,3,4-oxadiazole derivative 11 (MBC = 78.125 μg mL^−1^).

**Table 4:** MBC data for quinoline-oxadiazole derivatives 9–11 and 17a–e against different microbial strains

Comp. no.	MBC (μg mL^−1^)		
	*S. aureus*	*E. coli*	*C. albicans*
9	78.125	1250	39.06
10	156.25	625	19.53
11	312.5	625	78.125
17a	39.06	625	4.88
17b	39.06	625	9.77
17c	312.5	312.5	9.77
17d	19.53	1250	39.06
17e	19.53	1250	9.77
Neomycin	312.5	156.25	625

#### 2.2.9. Determination of the minimum biofilm inhibition concentration (MBIC)

The current study assessed the inhibition of biofilm formation in three microbial strains, including *S. aureus*, *E. coli* and *C. albicans*. To evaluate the biofilm inhibition ability of the quinoline-oxadiazole hybrids 9–11 and 17a–e under investigation, a crystal violet assay was used. The obtained results were contrasted with the reference positive drug, neomycin. Table 5 provides a summary of the assay data. With respect to *S. aureus*, compound 11 showed the best effective biofilm inhibition with a MBIC value of 9.77 μg mL^−1^, which is 31.99-times more active than the reference drug (MBIC = 312.5 μg mL^−1^). Conversely, compounds 9 and 17c with MBIC values of 39.06 and 19.53 μg mL^−1^, respectively, reduced the biofilm formation. In addition, compounds 10 and 17e exhibited four and two times the potency of the reference drug against *S. aureus*. Additionally, compounds 10, 11 and 17c demonstrated equipotent biofilm inhibition (MBIC = 9.77 μg mL^−1^), making them 16-times more active than neomycin, indicating that the *E. coli* microbial strain was the most susceptible. Compound 9 decreased the biofilm inhibition with a MBIC value of 19.53 μg mL^−1^, which is 8-times more active than the reference neomycin. Finally, with respect to the *C. albicans* microbial strain, compound 17c exhibited the best effective biofilm inhibition (9.77 μg mL^−1^), which is 63.98-time more potent than the reference drug neomycin. It should be noted that all of the examined quinoline compounds possess considerable and more potent biofilm inhibition activity (MBIC range of 19.53–78.125 μg mL^−1^) than the reference (MBIC = 625 μg mL^−1^), with an exception for compounds 17a and 17b (equal MBIC = 1250 μg mL^−1^). It should also be noted that compound 11 possessed broad spectrum biofilm inhibition against the *S. aureus* and *E. coli* microbial strains, with an identical MBIC score of 9.77 μg mL^−1^. Furthermore, compound 17c demonstrated equal MBIC activity against the *E. coli* and *C. albicans* strains.

**Table 5:** MIC of the biofilm inhibition data noted for the quinoline-oxadiazole derivatives 9–11 and 17a–e against different microbial strains

Comp. no.	MIC of biofilm inhibition (μg mL^−1^)		
	*S. aureus*	*E. coli*	*C. albicans*
9	39.06	19.53	39.06
10	78.125	9.77	19.53
11	9.77	9.77	19.53
17a	1250	1250	1250
17b	1250	1250	1250
17c	19.53	9.77	9.77
17d	625	1250	1250
17e	156.53	156.53	78.125
Neomycin	312.5	156.25	625

#### 2.2.10. Structure–activity relationship studies

In the current work, a structure–activity relationship study was conducted to give comprehensive insight into the effect of structural modifications on the activity against cancer and bacterial strains based on the mean IC_50_ values against cancer cell lines and the mean MIC values. Regarding the activity against cancer, it was revealed that the highest antiproliferative activity could be attained by substituting the 2-(2-(4-bromophenyl)quinolin-4-yl)-1,3,4-oxadiazole scaffold with 4-OH-phenyl (8b), NH_2_-phenyl (8e), thio-benzyl (11), allyl (12c), *N*-(4-acetylphenyl)-2-mercaptoacetamide (14), and 2-thione *N*-methyl piperazine (17c). However, it was shown that the best antimicrobial activity could be exhibited by substituting the 2-(2-(4-bromophenyl)quinolin-4-yl)-1,3,4-oxadiazole scaffold with 2-thione *N*-methyl morpholine (17a) and 2-thione *N*-methyl piperidine (17b).

### 2.3. *In silico* studies

#### 2.3.1. *In silico* physicochemical and ADMET properties

To predict the potential pharmacokinetic profile of the synthesized compounds, their physicochemical properties and ADME parameters were computationally evaluated using the Swiss ADME web platform. This *in silico* approach provided valuable insights into the compounds' absorption, distribution, metabolism, excretion, and toxicity, aiding in the early-stage assessment of their drug-like potential.^71^ Moreover, to assess the potential toxicity of the synthesized compounds, the pkCSM descriptor algorithm was employed. This computational approach predicts the toxicity profiles based on the compounds' structural features.^72^

**Fig. 7:**
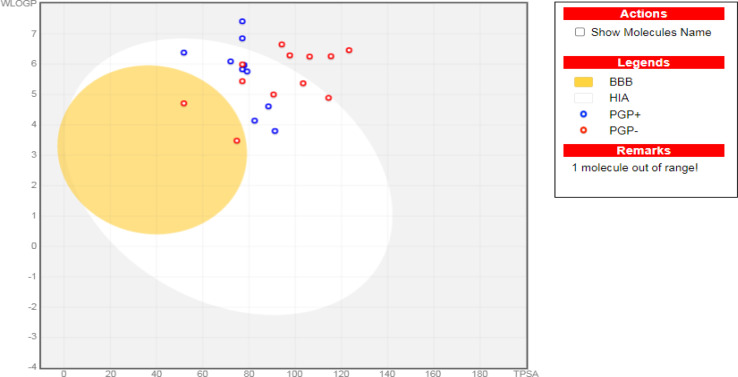
The boiled-egg representation for the synthesized quinoline derivatives (7–17e), as well as erlotinib as a reference control.

Accordingly, regarding their physicochemical features, except for compounds (8c, 10, 11, 12d, 14, 15a, 15b, and 17e), all synthesized compounds displayed high GIT absorption due to their feasible lipophilicity. Therefore, eligible oral bio-availabilities can be anticipated.^73,74^ Moreover, except for compound (7), all afforded compounds cannot pass through the blood–brain barrier. Thus, fewer CNS side effects can be assumed. Notably, compounds 7, 8c, 9, 10, 12a, 12c, 13, 14, 15a, 15b, 16, and 17e are not *P*-glycoprotein (*P*gp-) substrates (Tables 6, 7, and 8). Hence, better bioavailability could be assured, as shown in Fig. 7. Moreover, compound 8c did not show inhibition for the common hepatic metabolizing enzymes (CYP1A2, CYP2C19, CYP2C9, CYP2D6, and CYP3A4). Except for compounds 12d, 15a, and 17e, all of the synthesized quinoline derivatives match the Lipinski's rule of five,^75^ assuring their oral bioavailability. To further evaluate the compounds' bioavailability, we utilized the bioavailability radar tool provided by SwissADME. This visual representation offers a comprehensive assessment of drug-like properties within a hexagonal framework. Compounds falling within the optimal physicochemical space defined by the radar are considered to have favorable oral bioavailability. These radar plots are shown in the supplementary Fig. SI5.†

**Table 6:** The physicochemical, pharmacokinetics, and toxicity parameters of compounds (7–10)

		Comp. 7	Comp. 8a	Comp. 8b	Comp. 8c	Comp. 8d	Comp. 8e	Comp. 9	Comp. 10
Molecular properties	Molar refractivity	88.17	113.61	115.63	122.43	118.01	118.01	95.42	124.80
	TPSA (A^*z*^)	51.81	51.81	72.04	97.63	77.83	77.83	90.61	94.18
	log*P* o/w (WLOGP)	4.71	6.38	6.09	6.29	5.97	5.97	5.00	6.65
	Consensus log*P* o/w	3.96	5.39	5.01	4.61	4.84	4.84	4.29	5.47
	Water solubility	MS	PS	PS	PS	PS	PS	MS	PS
Pharmacokinetics parameters	GI absorption	High	High	High	Low	High	High	High	Low
	BBB permeant	Yes	No	No	No	No	No	No	No
	*P*-gp substrate	No	Yes	Yes	No	Yes	Yes	No	No
	CYP1A2 inhibitor	Yes	No	No	No	No	No	Yes	No
	CYP2C19 inhibitor	Yes	Yes	No	No	Yes	Yes	Yes	Yes
	CYP2C9 inhibitor	No	No	No	No	No	No	Yes	Yes
	CYP2D6 inhibitor	Yes	No	No	No	Yes	Yes	No	No
	CYP3A4 inhibitor	No	No	No	No	No	No	Yes	No
Drug/lead likeness	Drug likeness (lipinski)	Yes	Yes	Yes	Yes	Yes	Yes	Yes	Yes
	Lead likeness	No	No	No	No	No	No	No	No
Toxicity parameters	Ames toxicity	Yes	No	No	No	No	No	Yes	No
	Max. tolerated dose (log mg kg^−1^ per day)	0.362	0.649	0.626	0.596	0.624	0.633	0.4	0.671
	hERG I inhibitor	No	No	No	No	No	No	No	No
	hERG II inhibitor	No	Yes	Yes	Yes	Yes	Yes	Yes	Yes
	Oral rat acute toxicity (LD_50_) (mol kg^−1^)	2.229	2.347	2.77	2.677	2.993	3.074	2.512	2.786
	Oral rat chronic toxicity (LOAEL) (log mg kg^−1^ bw per day)	1.029	0.486	0.615	0.413	0.49	0.466	0.869	0.518
	Hepatotoxicity	Yes	Yes	Yes	Yes	Yes	Yes	No	Yes
	Minnow toxicity (log mM)	1.587	−0.692	−0.514	−2.631	−0.749	−0.467	1.396	−1.59

**Table 7:** The physicochemical, pharmacokinetics, and toxicity parameters of compounds (11–15a)

		Comp. 11	Comp. 12a	Comp. 12b	Comp. 12c	Comp. 12d	Comp. 13	Comp. 14	Comp. 15a
Molecular properties	Molar refractivity	124.38	99.89	104.70	109.03	134.31	106.47	143.92	133.73
	TPSA (A^*z*^)	77.11	77.11	77.11	77.11	77.11	114.41	123.28	106.21
	Log*P* o/w (WLOGP)	6.85	5.44	5.83	5.99	7.41	4.89	6.46	6.25
	Consensus log*P* o/w	5.81	4.63	4.95	5.17	6.34	4.03	5.12	5.17
	Water solubility	PS	MS	PS	PS	PS	MS	PS	PS
Pharmacokinetics parameters	GI absorption	Low	High	High	High	Low	High	Low	Low
	BBB permeant	No	No	No	No	No	No	No	No
	*P*-gp substrate	Yes	No	Yes	No	Yes	No	No	No
	CYP1A2 inhibitor	No	Yes	Yes	Yes	No	Yes	No	No
	CYP2C19 inhibitor	Yes	Yes	Yes	Yes	Yes	Yes	Yes	Yes
	CYP2C9 inhibitor	No	Yes	Yes	Yes	No	Yes	Yes	Yes
	CYP2D6 inhibitor	No	No	No	No	No	No	No	No
	CYP3A4 inhibitor	Yes	Yes	Yes	No	Yes	No	Yes	Yes
Drug/lead likeness	Drug likeness (lipinski)	Yes	Yes	Yes	Yes	No	Yes	Yes	No
	Lead likeness	No	No	No	No	No	No	No	No
Toxicity parameters	Ames toxicity	No	Yes	Yes	Yes	No	No	No	No
	Max. tolerated dose (log mg kg^−1^ per day)	0.692	0.346	0.493	0.574	0.671	1.035	0.736	0.764
	hERG I inhibitor	No	No	No	No	No	No	No	No
	hERG II inhibitor	Yes	Yes	Yes	Yes	Yes	No	Yes	Yes
	Oral rat acute toxicity (LD_50_) (mol kg^−1^)	2.547	2.317	2.264	2.213	2.531	2.604	3.091	3.009
	Oral rat chronic toxicity (LOAEL) (log mg kg^−1^ bw per day)	0.433	0.848	0.802	0.734	0.44	0.792	0.371	0.413
	Hepatotoxicity	Yes	No	No	No	Yes	Yes	Yes	Yes
	Minnow toxicity (log mM)	−2.429	0.929	0.54	0.091	−4.27	1.452	−1.724	−2.091

**Table 8:** The physicochemical, pharmacokinetics, and toxicity parameters of compounds (15b–17e)

		Comp. 15b	Comp. 16	Comp. 17a	Comp. 17b	Comp. 17c	Comp. 17d	Comp. 17e	Erlotinib
Molecular properties	Molar refractivity	140.22	115.60	125.47	129.19	131.10	136.00	154.43	111.40
	TPSA (A^*z*^)	115.44	103.41	88.41	79.18	91.21	82.42	79.18	74.73
	Log*P* o/w (WLOGP)	6.26	5.37	4.61	5.76	3.80	4.14	8.49	3.48
	Consensus log*P* o/w	5.10	4.77	4.32	5.17	4.01	4.26	6.78	3.28
	Water solubility	PS	PS	MS	PS	MS	PS	PS	MS
Pharmacokinetics parameters	GI absorption	Low	High	High	High	High	High	Low	High
	BBB permeant	No	No	No	No	No	No	No	Yes
	*P*-gp substrate	No	No	Yes	Yes	Yes	Yes	No	No
	CYP1A2 inhibitor	No	Yes	Yes	Yes	Yes	Yes	No	Yes
	CYP2C19 inhibitor	Yes	Yes	No	Yes	Yes	No	Yes	Yes
	CYP2C9 inhibitor	Yes	Yes	Yes	Yes	Yes	Yes	No	Yes
	CYP2D6 inhibitor	Yes	No	No	No	Yes	No	No	Yes
	CYP3A4 inhibitor	Yes	Yes	Yes	No	Yes	Yes	No	Yes
Drug/lead likeness	Drug likeness (lipinski)	Yes	Yes	Yes	Yes	Yes	Yes	No	Yes
	Lead likeness	No	No	No	No	No	No	No	No
Toxicity parameters	Ames toxicity	No	Yes	Yes	No	Yes	Yes	No	No
	Max. tolerated dose (log mg kg^−1^ per day)	0.761	0.537	0.276	0.31	0.415	0.434	0.494	0.355
	hERG I inhibitor	No	No	No	No	No	No	No	No
	hERG II inhibitor	Yes	Yes	Yes	Yes	Yes	Yes	Yes	Yes
	Oral rat acute toxicity (LD_50_) (mol kg^−1^)	3.049	2.406	2.767	2.725	2.752	2.755	3.034	3.058
	Oral rat chronic toxicity (LOAEL) (log mg kg^−1^ bw per day)	0.345	0.609	0.574	0.557	2.595	2.477	−0.569	1.558
	Hepatotoxicity	Yes	Yes	Yes	No	No	Yes	Yes	Yes
	Minnow toxicity (log mM)	−1.971	1.28	1.564	1.102	1.763	1.651	−3.411	−1.971

By employing the pkCSM descriptors algorithm protocol, it was revealed that compounds 7, 9, 12a, 12b, 12c, 16, 17a, 17c, and 17d could manifest Ames toxicity. Thus, a possible mutagenicity could be predicted.^76^ Additionally, all the synthesized quinoline derivatives are non-inhibitors of hERG I. Therefore, a cardiotoxic effect on the human heart's electrical activity cannot be assumed for these compounds.^77^ However, except for compounds 7 and 13, all synthesized compounds (including erlotinib) exhibit hERG II inhibitory activity, which raises concerns about possible cardiac arrhythmias,^78^ Notably, except for compounds 9, 12a, 12b, 12c, 17b, and 17c, all of the synthesized compounds are hepatotoxic.

## 3. Conclusions

By retaining the main pharmacophores of EGFR and DNA gyrase inhibitors, the synthesized compounds (7–17e) can emerge as promising lead anticancer and antimicrobial agents. The anti-proliferative activity was assured by the eligible cytotoxic activities of compounds (7–17e) against HepG2 and MCF-7 with IC_50_ values of 0.137–0.332 and 0.164–0.583 μg mL^−1^, respectively, in comparison to erlotinib, which showed IC_50_ values of 0.308 and 0.512 μg mL^−1^. Moreover, the investigated compounds could induce cell cycle arrest at the G1 phase. The eligible EGFR tyrosine kinase inhibitory potential of the investigated compounds (IC_50_ values of 0.14 and 0.18 μM for compounds 8c and 12d, respectively, compared to lapatinib (of 0.12 μM)) emphasized the paper hypothesis. Furthermore, the antimicrobial activity was assured by eligible MIC values against compounds 17b, 17d and 17e in particular, which displayed the most potent inhibitory activity with 4-, 16- and 8-fold greater potency, respectively, than the reference neomycin. The conducted molecular modeling assured the feasible binding pattern of the investigated compounds at the EGFR and DNA gyrase binding sites. Moreover, the established *in silico* studies assured the eligible pharmacokinetic profiles and toxicity parameters of the synthesized compounds. Accordingly, the synthesized drugs can undergo additional pre-clinical and clinical studies to determine their effectiveness as a double-edged sword for cancer and microbial infection treatment.

## 4. Experimental

### 4.1. Chemistry

#### 4.1.1. Procedure for the synthesis of 2-(4-bromophenyl)quinoline-4-carboxylic acid (1)

Synthesis of 2-(4-bromophenyl)quinoline-4-carboxylic acid (1) as reported in ref. 67 and 79.

#### 4.1.2. Procedure for synthesis of ethyl 2-(4-bromophenyl)quinoline-4-carboxylate (2)

Synthesis of ethyl 2-(4-bromophenyl)quinoline-4-carboxylate (2) as reported in ref. 67, 80 and 81.

#### 4.1.3. Procedure for synthesis of 2-(4-bromophenyl)quinoline-4-carbohydrazide (3)

Synthesis of 2-(4-bromophenyl)quinoline-4-carbohydrazide (3) as reported in ref. 67 and 81–83.

#### 4.1.4. Procedure for synthesis of *N*-(4-acetylphenyl)-2-chloroacetamide (4)

Synthesis of *N*-(4-acetylphenyl)-2-chloroacetamide (4) as reported in ref. 84.

#### 4.1.5. Procedure for synthesis of 2-chloro-*N*-aryl-acetamides (5a,b)

Synthesis of 2-chloro-*N*-aryl-acetamides (5a,b) as reported in ref. 67 and 85.

#### 4.1.6. Procedure for synthesis of ethyl-*N*-(2-(4-bromophenyl)quinoline-4-carbonyl)formohydrazonate (6)

Synthesis of ethyl-*N*-(2-(4-bromophenyl)quinoline-4-carbonyl)formohydrazonate (6) as reported in ref. 67.

Compound 6 (10 mmol, 3.98 g) was heated at 10 °C above its melting point for 30 min in an oil bath. After cooling the reaction, quinoline-oxadiazole molecule 7 was attained by crystallization from ethanol (70%).

Buff crystals, yield 61%, m.p. 140–142 °C. IR (KBr, cm^−1^): 3138 (CH aromatic), 2993 (CH aliphatic), 1537 (C=N). ^1^H NMR (400 MHz, DMSO-*d*_6_), *δ* ppm: 7.60–7.66 (m, 3H, Ar-H), 7.76 (t, 1H, Ar-H), 7.99 (d, 1H, *J* = 8.4 Hz, Ar-H), 8.04 (d, 2H, *J* = 8.4 Hz, Ar-H), 8.27 (s, 1H, Ar-H), 8.88 (d, 1H, *J* = 8.4 Hz, Ar-H), 9.57 (s, 1H, oxadiazole-H). ^13^C NMR (100 MHz, DMSO-*d*_6_), *δ* ppm: 121.17, 124.41, 124.78, 125.42, 127.35, 128.37, 129.33(2C), 130.00, 130.65, 132.27(2C), 137.52, 147.26, 147.93, 154.76, 167.19. MS *m*/*z* (%): 354.03 (M + 2, 17.31), 353.47 (M + 1, 12.36), 352.46 (M^+^, 18.72), 302.58 (100). Anal. calcd for C_17_H_10_BrN_3_O (352.19): C, 57.98; H, 2.86; N 11.93; found: C, 58.14; H, 3.02; N, 11.85.

A mixture of acid hydrazide 3 (10 mmol, 3.42 g), carboxylic acid derivatives (10 mmol) and phosphorous oxychloride (5 mL) was heated at 60 °C for 6–8 h, and then allowed to cool at room temperature. After the reaction mixture was added to ice-cold water, a saturated sodium bicarbonate solution was used to neutralize it. The obtained precipitate was crystallized from ethanol (70%) to give the corresponding oxadiazole product 8a–e.

Buff powder, yield 69%, m.p. 90–92 °C. IR (KBr, cm^−1^): 3070 (CH aromatic), 2954 (CH aliphatic), 1531 (C=N). ^1^H NMR (400 MHz, DMSO-*d*_6_), *δ* ppm: 7.52 (t, 2H, Ar-H), 7.59–7.63 (m, 1H, Ar-H), 7.76–7.90 (m, 3H, Ar-H), 7.92–7.96 (m, 3H, Ar-H), 8.15–8.28 (m, 3H, Ar-H), 8.47 (s, 1H, Ar-H), 8.80 (d, 1H, *J* = 8 Hz, Ar-H). ^13^C NMR (100 MHz, DMSO-*d*_6_), *δ* ppm: 117.81, 123.52, 124.58, 125.56, 126.30, 127.29(2C), 128.34(2C), 129.33(2C), 130.65, 131.78, 132.04(2C), 133.39, 137.45, 140.85, 143.92, 147.99, 154.77, 165.31, 166.00. MS *m*/*z* (%): 430.40 (M + 2, 31.26), 428.20 (M^+^, 30.16), 292.89 (100). Anal. calcd for C_23_H_14_BrN_3_O (428.29): C, 64.50; H, 3.29; N 9.81; found: C, 64.32; H, 3.50; N, 10.04.

White powder, yield 63%, m.p. 190–192 °C. IR (KBr, cm^−1^): 3099 (CH aromatic), 2993 (CH aliphatic), 1543 (C=N). ^1^H NMR (400 MHz, DMSO-*d*_6_), *δ* ppm: 5.75 (s, 1H, OH, D_2_O exchangeable), 7.47 (d, 2H, *J* = 8.4 Hz, Ar-H), 7.75–7.95 (m, 4H, Ar-H), 8.23–8.27 (m, 2H, Ar-H), 8.33 (d, 1H, *J* = 8.8 Hz, Ar-H), 8.39 (d, 2H, *J* = 7.2 Hz, Ar-H) 8.82 (s, 1H, Ar-H), 9.20 (d, 1H, *J* = 8.8 Hz, Ar-H). ^13^C NMR (100 MHz, DMSO-*d*_6_), *δ* ppm: 116.74(2C), 119.15(2C), 122.16, 123.27, 123.89, 124.86, 126.30, 128.33, 129.03(2C), 129.99, 131.05, 132.01(2C), 132.71, 140.52, 143.25, 147.98, 154.77, 163.65, 164.64. MS *m*/*z* (%): 446.02 (M + 2, 17.91), 443.82 (M^+^, 19.02), 325.62 (100). Anal. calcd for C_23_H_14_BrN_3_O_2_ (444.29): C, 62.18; H, 3.18; N 9.46; found: C, 62.40; H, 3.29; N, 9.58.

White powder, yield 66%, m.p. 288–290 °C. IR (KBr, cm^−1^): 3086 (CH aromatic), 2947 (CH aliphatic), 1546 (C=N). ^1^H NMR (400 MHz, DMSO-*d*_6_), *δ* ppm: 7.83–7.89 (m, 3H, Ar-H), 7.97 (t, 1H, Ar-H), 8.26 (d, 1H, *J* = 8.8 Hz, Ar-H), 8.40 (d, 2H, *J* = 8.8 Hz, Ar-H), 8.50 (d, 2H, *J* = 8.4 Hz, Ar-H), 8.60 (d, 2H, *J* = 8.4 Hz, Ar-H), 8.88 (s, 1H, Ar-H), 9.20 (d, 1H, *J* = 8 Hz, Ar-H). ^13^C NMR (100 MHz, DMSO-*d*_6_), *δ* ppm: 116.00, 121.86, 123.90, 124.00, 125.29, 126.65(2C), 127.29(2C), 128.33(2C), 128.39, 132.03, 133.39(2C), 133.77, 139.19, 141.33, 142.18, 147.48, 155.44, 166.00, 166.44. MS *m*/*z* (%): 475.22 (M + 2, 59.19), 473.60 (M^+^, 60.06), 44.39 (100). Anal. calcd for C_23_H_13_BrN_4_O_3_ (473.29): C, 58.37; H, 2.77; N 11.84; found: C, 58.51; H, 2.94; N, 12.07.

Buff powder, yield 62%, m.p. 220–222 °C. IR (KBr, cm^−1^): 3095 (CH aromatic), 2980 (CH aliphatic), 1573 (C=N). ^1^H NMR (400 MHz, DMSO-*d*_6_), *δ* ppm: 6.74 (d, 2H, *J* = 8.0 Hz, Ar-H), 7.82 (m, 3H, Ar-H), 7.94–8.28 (m, 5H, Ar-H + NH_2_ D_2_O exchangeable), 8.35–8.39 (m, 3H, Ar-H), 8.78 (s, 1H, Ar-H), 9.20 (d, 1H, *J* = 8.0 Hz, Ar-H). ^13^C NMR (100 MHz, DMSO-*d*_6_), *δ* ppm: 114.71(2C), 117.47, 122.17, 124.19, 125.93, 126.60, 127.66, 128.34, 128.62(2C), 129.03(2C), 129.68, 130.68, 132.04(2C), 142.20, 147.31, 149.06, 154.76, 164.33, 165.67. MS *m*/*z* (%): 445.95 (M + 2, 22.61), 443.72 (M^+^, 22.39), 400.91 (100). Anal. calcd for C_23_H_15_BrN_4_O (443.30): C, 62.32; H, 3.41; N 12.64; found: C, 62.56; H, 3.64; N, 12.85.

Brown powder, yield 64%, m.p. 177–179 °C, IR (KBr, cm^−1^): 3091 (CH aromatic), 2924 (CH aliphatic), 1537 (C=N). ^1^H NMR (400 MHz, DMSO-*d*_6_), *δ* ppm: 6.83 (t, 1H, Ar-H), 6.95 (t, 1H, Ar-H), 7.07 (d, 1H, *J* = 8.0 Hz, Ar-H), 7.35–7.36 (m, 1H, Ar-H), 7.62–7.94 (m, 4H, Ar-H), 8.08 (d, 1H, *J* = 8.8 Hz, Ar-H), 8.22 (s, 2H, NH_2_ D_2_O exchangeable), 8.37 (d, 2H, *J* = 8.0 Hz, Ar-H), 8.78 (s, 1H, Ar-H), 9.20 (d, 1H, *J* = 8.0 Hz, Ar-H). ^13^C NMR (100 MHz, DMSO-*d*_6_), *δ* ppm: 117.12, 118.77, 119.84, 121.48, 122.84, 123.21, 123.88, 125.57, 126.98, 127.96, 129.69(2C), 129.99, 130.67, 132.02(2C), 133.08, 134.73, 141.21, 147.99, 154.76, 165.69, 166.66 MS *m*/*z* (%): 445.47 (M + 2, 16.37), 442.91 (M^+^, 16.05), 237.34 (100). Anal. calcd for C_23_H_15_BrN_4_O (443.30): C, 62.32; H, 3.41; N 12.64; found: C, 62.50; H, 3.57; N, 12.79.

An equimolar amount of acid hydrazide 3 (10 mmol, 3.42 g) and potassium hydroxide (0.56 g) with carbon disulfide (2 mL) in absolute ethanol (20 mL) was heated under reflux for 12 h. After reaction completion, the excess solvent was evaporated, then neutralized with dil. HCl. The crystallized solid was separated from isopropanol to attain compound 9.

Yellow powder, yield 83%, m.p. 260–262 °C. IR (KBr, cm^−1^): 3155 (NH), 3086 (CH aromatic), 2899 (CH aliphatic), 1543 (C=N), 1238 (C=S). ^1^H NMR (400 MHz, DMSO-*d*_6_), *δ* ppm: 7.75–7.86 (m, 3H, Ar-H), 7.88 (t, 1H, Ar-H), 8.15 (d, 1H, *J* = 8.0 Hz, Ar-H), 8.21 (d, 2H, *J* = 8.0 Hz, Ar-H), 8.37 (s, 1H, Ar-H), 8.76 (d, 1H, *J* = 8.0 Hz, Ar-H), 11.13 (s, 1H, SH, D_2_O exchangeable). ^13^C NMR (100 MHz, DMSO-*d*_6_), *δ* ppm: 110.33, 118.37, 122.15, 123.88, 125.33, 127.61, 128.80(2C), 129.36, 130.55, 131.10(2C), 132.49, 136.54, 141.41, 148.67, 154.66. MS *m*/*z* (%): 386.53 (M + 2, 19.17), 384.51 (M^+^, 20.25), 293.51 (100). Anal. calcd for C_17_H_10_BrN_3_OS (384.25): C, 53.14; H, 2.62; N 10.94; found: C, 53.41; H, 2.86; N, 11.17.

Benzoyl chloride (10 mmol, 1.40 mL) was added dropwise to a well-stirred solution of 1,3,4-oxadiazole-2-thiol 9 (10 mmol, 3.84 g) in dioxane (20 mL), and the reaction mixture was stirred at ambient temperature for the entire night. After adding 10% sodium carbonate solution (25 mL) to the reaction mixture, the resulting solid was crystallized from ethanol to get compound 10.

Buff powder, yield 70%, m.p. 220–222 °C. IR (KBr, cm^−1^): 3061 (CH aromatic), 2920 (CH aliphatic), 1707 (C=O), 1589 (C=N). ^1^H NMR (400 MHz, DMSO-*d*_6_), *δ* ppm: 7.50 (t, 2H, Ar-H), 7.61 (t, 1H, Ar-H), 7.76–7.81 (m, 3H, Ar-H), 7.90–7.96 (m, 3H, Ar-H), 8.19 (d, 1H, *J* = 8.0 Hz, Ar-H), 8.27 (d, 2H, *J* = 8 Hz, Ar-H), 8.45 (s, 1H, Ar-H), 8.80 (d, 1H, *J* = 8 Hz, Ar-H). ^13^C NMR (100 MHz, DMSO-*d*_6_), *δ* ppm: 118.48, 122.55, 123.91, 124.58, 125.26, 127.96, 128.37(2C), 128.65(2C), 129.33, 130.38, 130.68, 131.05, 131.74(2C), 132.71(2C), 136.48, 154.77, 158.84, 159.89, 167.72, 178.86. MS *m*/*z* (%): 490.09 (M + 2, 25.22), 488.91 (M^+^, 26.64), 363.31 (100). Anal. calcd for C_24_H_14_BrN_3_O_2_S (488.36): C, 59.03; H, 2.89; N 8.60; found: C, 59.31; H, 3.02; N, 8.87.

Benzyl chloride (10 mmol, 1.26 g) was added to a well-stirred suspension of 1,3,4-oxadiazole-2-thiol 9 (10 mmol, 3.84 g) and anhydrous potassium carbonate (10 mmol, 1.38 g) in dry acetone (20 mL). The reaction mixture was heated to reflux for 6 h, and then filtered off. Compound 11 was produced by evaporating the excess solvent and crystallizing it from ethanol.

To a well-stirred suspension of 1,3,4-oxadiazole-2-thiol 9 (10 mmol, 3.84 g) and anhydrous potassium carbonate (10 mmol, 1.38 g) in dry acetone (20 mL), benzyl chloride (10 mmol, 1.26 g) was added. The reaction mixture was heated under reflux for 6 h, and then filtered off. Excess solvent was evaporated, and the obtained solid was dried and crystallized from ethanol to give compound 11.

Yellowish green powder, yield 70%, m.p. 142–144 °C. IR (KBr, cm^−1^): 3099 (CH aromatic), 2929 (CH aliphatic), 1587 (C=N). ^1^H NMR (400 MHz, DMSO-*d*_6_), *δ* ppm: 4.62 (s, 2H, CH_2_), 7.24–7.36 (m, 3H, Ar-H), 7.49 (d, 2H, *J* = 7.2 Hz, Ar-H), 7.67–7.69 (m, 3H, Ar-H), 7.82 (t, 1H, Ar-H), 8.06–8.29 (m, 4H, Ar-H), 8.83 (d, 1H, *J* = 8.4 Hz, Ar-H). ^13^C NMR (100 MHz, DMSO-*d*_6_), *δ* ppm: 33.16, 117.36, 121.99, 124.39, 124.52, 125.82, 125.91, 126.21(2C), 126.59, 129.25, 129.63(2C), 129.81(2C), 130.57, 130.83, 131.63(2C), 142.34, 143.10, 148.17, 155.38, 166.40. MS *m*/*z* (%): 476.74 (M + 2, 38.62), 474.76 (M^+^, 37.00), 437.47 (100). Anal. calcd for C_24_H_16_BrN_3_OS (474.38): C, 60.77; H, 3.40; N 8.86; found: C, 60.90; H, 3.62; N, 9.04.

The alkylating agent (20 mmol) was added to a well-stirred suspension of 1,3,4-oxadiazole-2-thiol 9 (10 mmol, 3.84 g) and potassium hydroxide (20 mmol, 1.12 g) in a mixture of ethanol (20 mL) and water (10 mL). The reaction mixture was agitated at 50–60 °C for 4–6 h. Compounds 12a–d were produced by filtering out, drying and crystallizing the produced solid from ethanol.

White powder, yield 70%, m.p. 170–172 °C. IR (KBr, cm^−1^): 3057 (CH aromatic), 2931 (CH aliphatic), 1571 (C=N). ^1^H NMR (400 MHz, DMSO-*d*_6_), *δ* ppm: 2.87 (3, 3H, CH_3_), 7.75–7.79 (m, 3H, Ar-H), 7.89 (t, 1H, Ar-H), 8.16 (d, 1H, *J* = 8.4 Hz, Ar-H), 8.26 (d, 2H, *J* = 8 Hz, Ar-H), 8.51 (s, 1H, Ar-H), 9.00 (d, 1H, *J* = 8.4 Hz, Ar-H). ^13^C NMR (100 MHz, DMSO-*d*_6_), *δ* ppm: 14.92, 119.84, 122.17, 123.22, 126.31, 127.66, 129.31(2C), 130.02, 130.40, 132.02(2C), 138.80, 144.30, 149.04, 153.79, 163.66, 165.00. MS *m*/*z* (%): 400.18 (M + 2, 21.71), 398.73 (M^+^, 22.46), 258.95(100). Anal. calcd for C_18_H_12_BrN_3_OS (398.28): C, 54.28; H, 3.04; N 10.55; found: C, 54.56; H, 3.21; N, 10.79.

White powder, yield 73%, m.p. 121–123 °C. IR (KBr, cm^−1^): 3055 (CH aromatic), 2962 (CH aliphatic), 1573 (C=N). ^1^H NMR (400 MHz, DMSO-*d*_6_), *δ* ppm: 1.49 (t, 3H, CH_3_), 3.31–3.44 (q, 2H, CH_2_), 7.74–7.79 (m, 3H, Ar-H), 7.89 (t, 1H, Ar-H), 8.15 (d, 1H, *J* = 8 Hz, Ar-H), 8.24 (d, 2H, *J* = 8.4 Hz, Ar-H), 8.47 (s, 1H, Ar-H), 8.98 (d, 1H, *J* = 8 Hz, Ar-H). ^13^C NMR (100 MHz, DMSO-*d*_6_), *δ* ppm: 14.87, 27.80, 118.11, 120.80, 122.16, 125.94, 127.29, 129.70, 129.99(2C) 130.36, 131.34, 132.03(2C), 137.15, 148.66, 155.15, 163.64, 165.31 MS *m*/*z* (%): 414.16 (M + 2, 22.22), 412.18 (M^+^, 22.67), 190.72(100). Anal. calcd for C_19_H_14_BrN_3_OS (412.31): C, 55.35; H, 3.42; N 10.19; found: C, 55.17; H, 3.64; N, 10.45.

White powder, yield 69%, m.p. 117–119 °C. IR (KBr, cm^−1^): 3084 (CH aromatic), 2980 (CH aliphatic), 1598 (C=N). ^1^H NMR (400 MHz, DMSO-*d*_6_), *δ* ppm: 4.10 (d, 2H, *J* = 6.8 Hz, SCH_2_), 5.24 (d, 1H, *J*_cis_ = 10 Hz, CH=CHH̲), 5.43 (d, 1H, *J*_trans_ = 17.2 Hz, –CH=CH̲H), 6.00–6.13 (m, 1H, CH=CH_2_), 7.74–7.78 (m, 3H, Ar-H), 7.88 (t, 1H, Ar-H), 8.13 (d, 1H, *J* = 8 Hz, Ar-H), 8.23 (d, 2H, *J* = 7.6 Hz, Ar-H), 8.44 (s, 1H, Ar-H), 8.97 (d, 1H, *J* = 8 Hz, Ar-H). ^13^C NMR (100 MHz, DMSO-*d*_6_), *δ* ppm: 34.90, 118.10, 119.84, 121.48, 121.84, 123.89, 125.57, 127.96, 129.03(2C), 129.68, 130.69, 132.41(2C), 133.07, 136.47, 148.00, 154.76, 163.65, 165.69. MS *m*/*z* (%): 426.79 (M + 2, 17.69), 424.77 (M^+^, 18.08), 222.87(100). Anal. calcd for C_20_H_14_BrN_3_OS (424.32): C, 56.61; H, 3.33; N 9.90; found: C, 56.86; H, 3.50; N, 10.14.

White powder, yield 72%, m.p. 176–178 °C. IR (KBr, cm^−1^): 3080 (CH aromatic), 2972 (CH aliphatic), 1587 (C=N). ^1^H NMR (400 MHz, DMSO-*d*_6_), *δ* ppm: 4.28 (d, 2H, *J* = 6.4 Hz, SCH_2_), 6.50–6.54 (m, 1H, =CH̲–CH_2_), 6.80 (d, 1H, *J* = 15.6 Hz, =CH-ph), 7.23–7.29 (m, 3H, Ar-H), 7.42 (d, 2H, *J* = 6.8 Hz, Ar-H), 7.71–7.77 (m, 3H, Ar-H), 7.85 (t, 1H, Ar-H), 8.12 (d, 1H, *J* = 8 Hz, Ar-H), 8.19 (d, 2H, *J* = 7.6 Hz, Ar-H), 8.43 (s, 1H, Ar-H), 8.96 (d, 1H, *J* = 8.4 Hz, Ar-H). ^13^C NMR (100 MHz, DMSO-*d*_6_), *δ* ppm: 35.35, 122.18, 122.64, 124.49, 124.93, 125.83, 126.27, 126.73, 129.23(2C), 129.71(2C), 129.93(2C), 130.35, 131.29(2C), 132.39, 134.46, 136.32, 137.17, 139.95, 148.80, 155.14, 162.89, 165.13. MS *m*/*z* (%): 502.37 (M + 2, 23.55), 500.27 (M^+^, 25.10), 434.76(100). Anal. calcd for C_26_H_18_BrN_3_OS (500.41): C, 62.41; H, 3.63; N 8.40; found: C, 62.32; H, 3.79; N, 8.67.

A mixture of 1,3,4-oxadiazole-2-thiol 9 (10 mmol, 3.84 g) and 2-chloroacetic acid (10 mmol, 0.95 g) in methylene chloride (20 mL) containing a few drops of TEA was heated under reflux for 10 h. After completion of the reaction, the excess solvent was evaporated under vacuum and the crude precipitate was crystallized from ethanol to give compound 13.

White powder, yield 69%, m.p. 258–260 °C. IR (KBr, cm^−1^): 3419 (OH), 3080 (CH aromatic), 2983 (CH aliphatic), 1716 (C=O), 1598 (C=N). ^1^H NMR (400 MHz, DMSO-*d*_6_), *δ* ppm: 4.19 (s, 2H, CH_2_), 5.72 (s, 1H, OH, D_2_O exchangeable), 7.76–7.80 (m, 3H, Ar-H), 7.90 (t, 1H, Ar-H), 8.17 (d, 1H, *J* = 8.4 Hz, Ar-H), 8.29 (d, 2H, *J* = 8.00 Hz, Ar-H), 8.54 (s, 1H, Ar-H), 9.00 (d, 1H, *J* = 8.0 Hz, Ar-H). ^13^C NMR (100 MHz, DMSO-*d*_6_), *δ* ppm: 36.62, 118.54, 122.91, 124.89, 125.91, 128.57, 128.93, 129.55(2C), 130.21, 131.28 132.24(2C), 136.62, 148.27, 155.29, 164.04, 166.34, 169.00. MS *m*/*z* (%): 444.62 (M + 2, 20.14), 442.56 (M^+^, 21.06), 326.68(100). Anal. calcd for C_19_H_12_BrN_3_O_3_S (442.29): C, 51.60; H, 2.73; N 9.50; found: C, 51.78; H, 2.90; N, 9.43.

An equimolar amount of 1,3,4-oxadiazole-2-thiol 9 (10 mmol, 3.84 g) and compound 4 (10 mmol, 2.11 g) in methylene chloride (20 mL) containing a few drops of TEA was heated under reflux for 6 h. The reaction mixture was filtered, and the resulting solid thus obtained was crystallized from ethanol to give compound 14.

White powder, yield 86%, m.p. 219–221 °C. IR (KBr, cm^−1^): 3446 (NH), 3061 (CH aromatic), 2927 (CH aliphatic), 1670 (C=O), 1597 (C=N). ^1^H NMR (400 MHz, DMSO-*d*_6_), *δ* ppm: 2.57 (s, 3H, CH_3_), 4.51 (s, 2H, CH_2_), 7.71–7.75 (m, 5H, Ar-H), 7.87–7.93 (m, 3H, Ar-H), 8.16 (d, 1H, *J* = 8.4 Hz, Ar-H), 8.21 (d, 2H, *J* = 8 Hz, Ar-H), 8.49 (s, 1H, Ar-H), 8.98 (d, 1H, *J* = 8.4 Hz, Ar-H), 10.90 (s, 1H, NH, D_2_O exchangeable). MS *m*/*z* (%): 561.96 (M + 2, 17.69), 559.09 (M^+^, 18.60), 404.96(100). Anal. calcd for C_27_H_19_BrN_4_O_3_S (559.44): C, 57.97; H, 3.42; N 10.01; found: C, 58.12; H, 3.61; N, 10.29.

The corresponding acetamide derivative 5a,b (10 mmol) was added to a well-stirred solution of 1,3,4-oxadiazole-2-thiol 9 (10 mmol, 3.84 g) and potassium hydroxide (20 mmol, 1.12 g) in a mixture of ethanol (20 mL) and water (10 mL). The reaction mixture was heated to reflux for 6–8 h. Compound 15a,b was produced by filtering out, drying and crystallizing the produced solid from ethanol.

White powder, yield 50% (2.4 g), m.p. 292–294 °C. IR (KBr, cm^−1^): 3429 (NH), 3061 (CH aromatic), 2968 (CH aliphatic), 1672 (C=O), 1587 (C=N). ^1^HNMR (400 MHz, DMSO-*d*_6_), *δ* ppm: 4.27 (s, 2H, CH_2_), 7.07 (t, 1H, Ar-H), 7.43 (d, 2H, *J* = 7.6 Hz, Ar-H), 7.57 (t, 1H, Ar-H), 7.70 (d, 2H, *J* = 7.7 Hz, Ar-H), 7.80 (t, 2H, Ar-H), 7.86 (t, 1H, Ar-H), 8.17 (d, 2H, *J* = 8.Hz, Ar-H), 8.26 (d, 1H, *J* = 8.4 Hz, Ar-H), 8.38 (s, 1H, Ar-H) 9.12 (d, 1H, *J* = 8.4 Hz, Ar-H), 10.96 (s, 1H, NH, D_2_O exchangeable). ^13^C NMR (100 MHz, DMSO-*d*_6_), *δ* ppm: 34.00, 117.20, 118.86, 119.91, 120.87(2C), 122.54, 123.89, 128.94(2C), 129.92(2C), 130.60, 131.59(2C), 133.23, 134.60, 135.56, 138.29, 141.97, 148.67, 149.94, 154.62, 163.65, 168.03. MS *m*/*z* (%): 519.48 (M + 2, 46.21), 517.38 (M^+^, 43.91), 195.66(100). Anal. calcd for C_25_H_17_BrN_4_O_2_S (517.40): C, 58.04; H, 3.31; N 10.83; found: C, 58.31; H, 3.47; N, 11.04.

Grayish white powder, yield 55%, m.p. 565–267 °C. IR (KBr, cm^−1^): 3446 (NH), 3078 (CH aromatic), 2956 (CH aliphatic), 1651 (C=O), 1591 (C=N). ^1^H NMR (400 MHz, DMSO-*d*_6_), *δ* ppm: 3.83 (s, 3H, OCH_3_), 4.24 (s, 2H, CH_2_), 7.10 (d, 2H, *J* = 6.4 Hz, Ar-H), 7.33 (d, 2H, *J* = 6 Hz, Ar-H), 7.61–7.78 (m, 4H, Ar-H), 8.18–8.36 (m, 4H, Ar-H), 9.13 (d, 1H, *J* = 7.2 Hz. Ar-H), 10.75 (s, 1H, NH, D_2_O exchangeable). ^13^C NMR (100 MHz, DMSO-*d*_6_), *δ* ppm: 33.63, 56.06, 114.54(2C), 116.52, 117.58, 119.90, 124.21(2C), 125.28, 129.24, 129.55, 129.91(2C), 130.60, 132.27(2C), 132.93, 137.30, 138.97, 148.64, 149.28, 153.35, 155.31, 160.05, 166.67. MS *m*/*z* (%): 549.92 (M + 2, 24.86), 547.22 (M^+^, 28.38), 398.33 (100). Anal. calcd for C_26_H_19_BrN_4_O_3_S (547.43): C, 57.05; H, 3.50; N 10.23; found: C, 57.29; H, 3.62; N, 10.51.

Ethyl chloroacetate (10 mmol, 1.22 g) was added to a well-stirred suspension of 1,3,4-oxadiazole-2-thiol 9 (10 mmol, 3.84 g) and anhydrous potassium carbonate (10 mmol, 1.38 g) in dry acetone (20 mL). The reaction mixture was heated to reflux for 5 h. Compound 6 was produced by filtering out, drying and crystallizing the produced precipitate from ethanol.

White powder, yield 87%, m.p. 310–312 °C. IR (KBr, cm^−1^): 3077 (CH aromatic), 2983 (CH aliphatic), 1739 (C=O), 1588 (C=N). ^1^H NMR (400 MHz, DMSO-*d*_6_), *δ* ppm: 1.20 (t, 3H, CH_3_), 4.17–4.22 (q, 2H, CH_2_), 4.41 (s, 2H, CH_2_), 7.79–7.83 (m, 3H, Ar-H), 7.93 (t, 1H, Ar-H), 8.21 (d, 1H, *J* = 8.4 Hz, Ar-H), 8.31 (d, 2H, *J* = 8.8 Hz, Ar-H), 8.59 (s, 1H, Ar-H), 9.00 (d, 1H, *J* = 8.4 Hz, Ar-H). ^13^C NMR (100 MHz, DMSO-*d*_6_), *δ* ppm: 14.14, 32.14, 62.29, 118.74, 122.04, 124.58, 125.55, 126.22, 129.01, 129.66(2C), 130.38, 130.68, 132.04(2C), 135.47, 141.22, 148.62, 155.22, 166.38, 167.66. MS *m*/*z* (%): 472.95 (M + 2, 23.61), 470.52 (M^+^, 25.19), 321.42(100). Anal. calcd for C_21_H_16_BrN_3_O_3_S (470.34): C, 53.63; H, 3.43; N 8.93; found: C, 53.88; H, 3.61; N, 9.12.

A suspension of 1,3,4-oxadiazole-2-thiol 9 (10 mmol, 3.84 g) and an appropriate secondary amine (10 mmol) was heated under reflux in ethanol (30 mL) with 36% formaldehyde (20 mmol) for 4–6 h. After cooling to room temperature, the resultant solid was crystallized from ethanol to get compound 17a–e.

Buff powder, yield 82%, m.p. 210–212 °C. IR (KBr, cm^−1^): 3059 (CH aromatic), 2974 (CH aliphatic), 1587 (C=N), 1267 (C=S). ^1^H NMR (400 MHz, DMSO-*d*_6_), *δ* ppm: 2.82–2.86 (m, 4H, CH_2_–N–CH_2_), 3.60–3.62 (m, 4H, CH_2_–O–CH_2_), 5.18 (s, 2H, CH_2_), 7.78–7.81 (m, 3H, Ar-H), 7.91 (t, 1H, Ar-H), 8.20 (d, 1H, *J* = 8.0 Hz. Ar-H), 8.28 (d, 2H, *J* = 8.8 Hz, Ar-H), 8.45 (s, 1H, Ar-H), 8.95 (d, 1H, *J* = 8.0 Hz, Ar-H), ^13^C NMR (100 MHz, DMSO-*d*_6_), *δ* ppm: 53.95(2C), 66.38(2C), 79.88, 122.55, 123.38, 124.87, 125.93, 126.61, 127.63, 128.61(2C), 129.93, 130.68, 131.75(2C), 137.82, 143.88, 155.15, 157.82, 179.34. MS *m*/*z* (%): 485.95 (M + 2, 17.04), 483.52 (M^+^, 14.99), 437.94 (100). Anal. calcd for C_22_H_19_BrN_4_O_2_S (483.38): C, 54.66; H, 3.96; N 11.59; found: C, 54.79; H, 4.12; N, 11.78.

Yellow powder, yield 77%, m.p. 192–194 °C, IR (KBr, cm^−1^): 3014 (CH aromatic), 2933 (CH aliphatic), 1587 (C=N), 1246 (C=S). ^1^H NMR (400 MHz, DMSO-*d*_6_), *δ* ppm: 1.35–1.64 (m, 6H, piperidine), 2.73–3.01 (m, 4H, piperidine), 5.17 (s, 2H, CH_2_), 7.76–7.88 (m, 3H, Ar-H), 7.87 (t, 1H, Ar-H), 8.16 (d, 1H, *J* = 8.0 Hz. Ar-H), 8.26 (d, 2H, *J* = 8.4 Hz, Ar-H), 8.36 (s, 1H, Ar-H), 9.15 (d, 1H, *J* = 8.0 Hz, Ar-H). ^13^C NMR (100 MHz, DMSO-*d*_6_), *δ* ppm: 22.23, 23.31(2C), 44.68(2C), 83.48, 116.21, 122.53, 124.69, 126.24, 126.60, 128.27, 129.25(2C), 130.93, 131.27, 132.25(2C), 137.60, 149.27, 155.00, 155.97, 181.05. MS *m*/*z* (%): 483.94 (M + 2, 36.44), 481.27 (M^+^, 36.79), 467.04 (100). Anal. calcd for C_23_H_21_BrN_4_OS (481.41): C, 57.38; H, 4.40; N 11.64; found: C, 57.45; H, 4.63; N, 11.90.

Yellow powder, yield 74%, m.p. 219–221 °C. IR (KBr, cm^−1^): 3423 (NH), 3064 (CH aromatic), 2931 (CH aliphatic), 1593 (C=N), 1249 (C=S). ^1^H NMR (400 MHz, DMSO-*d*_6_), *δ* ppm: 1.04 (s, 1H, NH, D_2_O exchangeable), 2.61 (s, 4H, piperazine), 2.91–3.07 (s, 4H, piperazine), 5.14 (s, 2H, CH_2_), 7.73–7.78 (m, 3H, Ar-H), 7.86 (t, 1H, Ar-H), 8.14 (d, 1H, *J* = 8.0 Hz, Ar-H), 8.26 (d, 2H, *J* = 7.6 Hz, Ar-H), 8.32 (s, 1H, Ar-H), 9.25 (d, 1H, *J* = 8.0 Hz, Ar-H). MS *m*/*z* (%): 484.08 (M + 2, 24.53), 481.98 (M^+^, 26.48), 284.45 (100). Anal. calcd for C_22_H_20_BrN_5_OS (482.40): C, 54.78; H, 4.18; N 14.52; found: C, 54.97; H, 4.40; N, 14.76.

Yellow powder, yield 80%, m.p. 156–158 °C. IR (KBr, cm^−1^): 3055 (CH aromatic), 2995 (CH aliphatic), 1589 (C=N), 1265 (C=S). ^1^H NMR (400 MHz, DMSO-*d*_6_), *δ* ppm: 2.83 (s, 3H, CH_3_), 3.18 (s, 4H, piperazine), 3.96 (s, 4H, piperazine), 5.23 (s, 2H, CH_2_), 7.65 (t, 1H, Ar-H), 7.71 (d, 2H, *J* = 8.4 Hz, Ar-H), 7.77 (t, 1H, Ar-H), 7.95 (d, 1H, *J* = 8.0 Hz, Ar-H), 8.07–8.11 (m, 3H, Ar-H), 8.85 (s, 1H, Ar-H). ^13^C NMR (100 MHz, DMSO-*d*_6_), *δ* ppm: 18.67, 53.39(2C), 56.43(2C), 69.34, 121.19, 121.87, 123.60, 125.24, 125.56, 128.30, 128.55, 129.42(2C), 130.23, 130.84, 132.18(2C), 137.21, 154.62, 154.72, 180.76. MS *m*/*z* (%): 498.15 (M + 2, 19.76), 496.72 (M^+^, 20.59), 323.33 (100). Anal. calcd for C_23_H_22_BrN_5_OS (496.43): C, 55.65; H, 4.47; N 14.11; found: C, 55.81; H, 4.63; N, 13.98.

Buff powder, yield 77%, m.p. 266–286 °C. IR (KBr, cm^−1^): 3055 (CH aromatic), 2997 (CH aliphatic), 1573 (C=N), 1238 (C=S). ^1^H NMR (400 MHz, DMSO-*d*_6_), *δ* ppm: 5.53 (s, 2H, CH_2_), 6.80 (t, 2H, Ar-H), 7.05 (d, 4H, *J* = 8 Hz, Ar-H), 7.21 (t, 4H, Ar-H), 7.71–7.76 (m, 3H, Ar-H), 7.87 (t, 1H, Ar-H), 8.13 (d, 1H, *J* = 8.4 Hz, Ar-H), 8.19 (d, 2H, *J* = 8.4 Hz, Ar-H), 8.33 (s, 1H, Ar-H), 8.74 (d, 1H, *J* = 8.4 Hz, Ar-H). MS *m*/*z* (%): 567.00 (M + 2, 15.43), 565.25 (M^+^, 17.63), 322.87 (100). Anal. calcd for C_30_H_21_BrN_4_OS (565.49): C, 63.72; H, 3.74; N, 9.91; found: C, 63.54; H, 3.89; N, 10.13.

### 4.2. Biological evaluation

See Appendix B.†

### 4.3. *In silico* studies

#### 4.3.1. Physicochemical, ADMET, and pharmacokinetic properties prediction

The Swiss Institute of Bioinformatics offers a free online tool called Swiss ADME that helps scientists predict how well a chemical compound will be absorbed, distributed, metabolized, and excreted in the body. To use this tool, scientists input the chemical structure of the compound in a specific format called SMILES. The tool then analyzes the structure and provides information about the compound's potential behavior in the body, which is important for determining its suitability as a drug.^71^ In addition to predicting drug-like properties, the researchers also assessed the potential toxicity of the compounds using an online tool called pkCS.^72^

## Conflicts of interest

The authors declare that there are no potential conflicts of interest.

## Supplementary Material

RA-014-D4RA06712F-s001
