# Supplementary material for: Design and synthesis of novel 2-(2-(4-bromophenyl)quinolin-4-yl)-1,3,4-oxadiazole derivatives as anticancer and antimicrobial candidates: in vitro and in silico studies
Source: RSC Adv. 2024 Oct 25;14(46):34005–26. doi: 10.1039/d4ra06712f (PMC11505673; doi:10.1039/d4ra06712f)
Supplement: RA-014-D4RA06712F-s001 [file RA-014-D4RA06712F-s001.pdf]

Supplementary Information (SI) for RSC Advances.
This journal is © The Royal Society of Chemistry 2024

Design and Synthesis of Novel 2-(2-(4-bromophenyl)quinolin-4-yl)-1,3,4-oxadiazole Derivatives as Anticancer and Antimicrobial candidates: *In vitro* and *In silico* studies

Noha Ryad [1], Ayman Abo Elmaaty [2], Samy Selim [3,*], Mohammed S. Almuhayawi [4], Soad K. Al Jaouni [5], Mohamed S. Abdel-Aziz [6], Arwa Sultan Alqahtani [7], Islam Zaki [8, *] and Lina M. A. Abdel Ghany [9]

[1] Pharmaceutical Organic Chemistry Department, College of Pharmaceutical Sciences and Drug Manufacturing, Misr University for Science and Technology, 6th of October City, Giza, P.O. Box 77, Egypt; [2] Medicinal Chemistry Department, Faculty of Pharmacy, Port Said University, Port Said 42526, Egypt; [3] Department of Clinical Laboratory Sciences, College of Applied Medical Sciences, Jouf University, Sakaka 72388, Saudi Arabia; [4] Department of Clinical Microbiology and Immunology, Faculty of Medicine, King Abdulaziz University, Jeddah 21589, Saudi Arabia; [5] Department of Hematology/Oncology, Yousef Abdulatif Jameel Scientific Chair of Prophetic Medicine Application, Faculty of Medicine, King Abdulaziz University, Jeddah 21589, Saudi Arabia; [6] Microbial Chemistry Department, Biotechnology Research Institute, National Research Centre, Cairo, Egypt; [7] Department of Chemistry, College of Science, Imam Mohammad Ibn Saud Islamic University(IMSIU), P.O. Box, 90950, Riyadh 11623, Saudi Arabia; [8] Pharmaceutical Organic Chemistry Department, Faculty of pharmacy, Port Said University, Port Said, Egypt; [9] Pharmaceutical Organic Chemistry Department, College of Pharmaceutical Sciences and Drug Manufacturing, Misr University for Science and Technology, 6th of October City, Giza, P.O. Box 77, Egypt

**** To whome correspondence should be addressed***

1. Islam Zaki, PhD. Pharmaceutical Organic Chemistry Department, Faculty of pharmacy, Port Said University, Port Said, Egypt

***E-mail address*:** eslam.zaki@pharm.psu.edu.eg **(**Zaki, I.**)**

2. Samy Selim, Department of Clinical Laboratory Sciences, College of Applied Medical Sciences, Jouf University, Sakaka 72388, Saudi Arabia

***E-mail address*:** sabdulsalam@ju.edu.sa **(**Selim, S.**)**

Appendix A

Part 1: Spectral data:

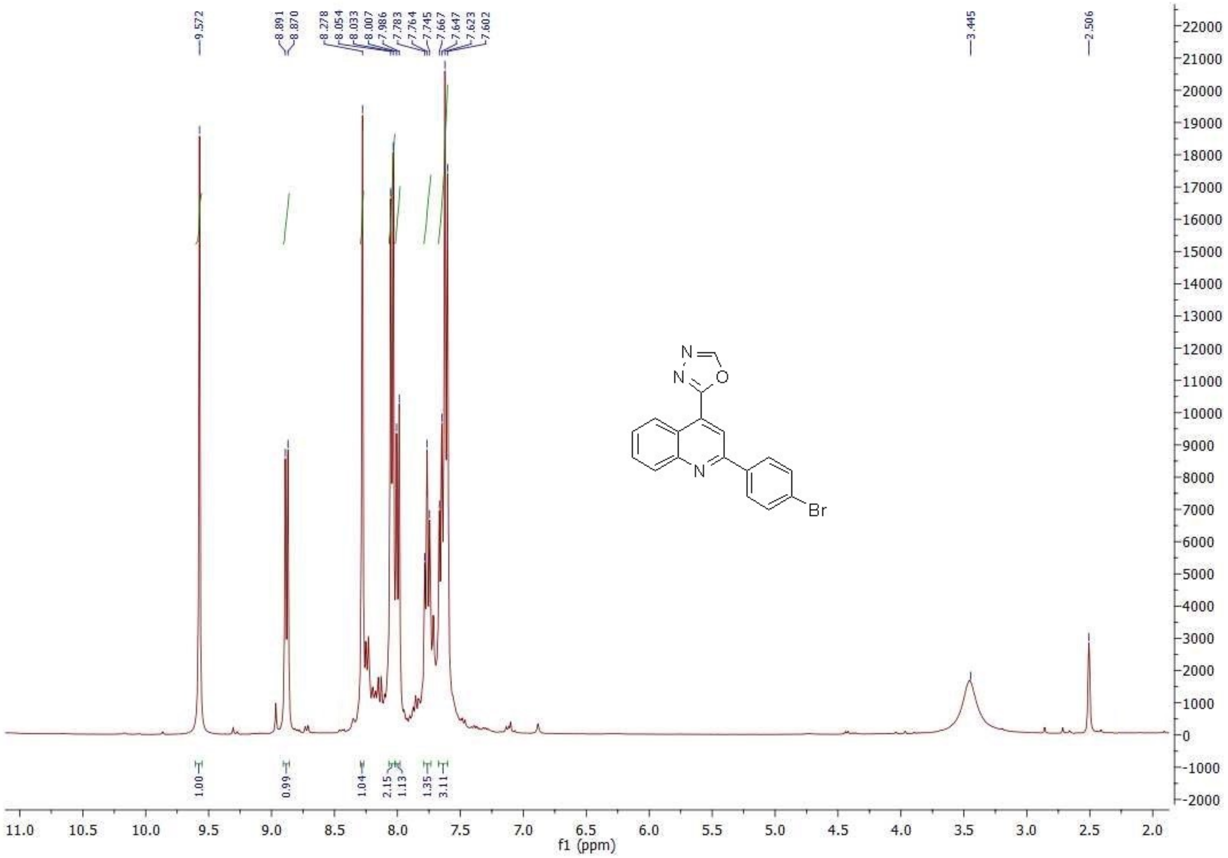

Figure S1: ^{1}HNMR spectrum of the compound **7**.

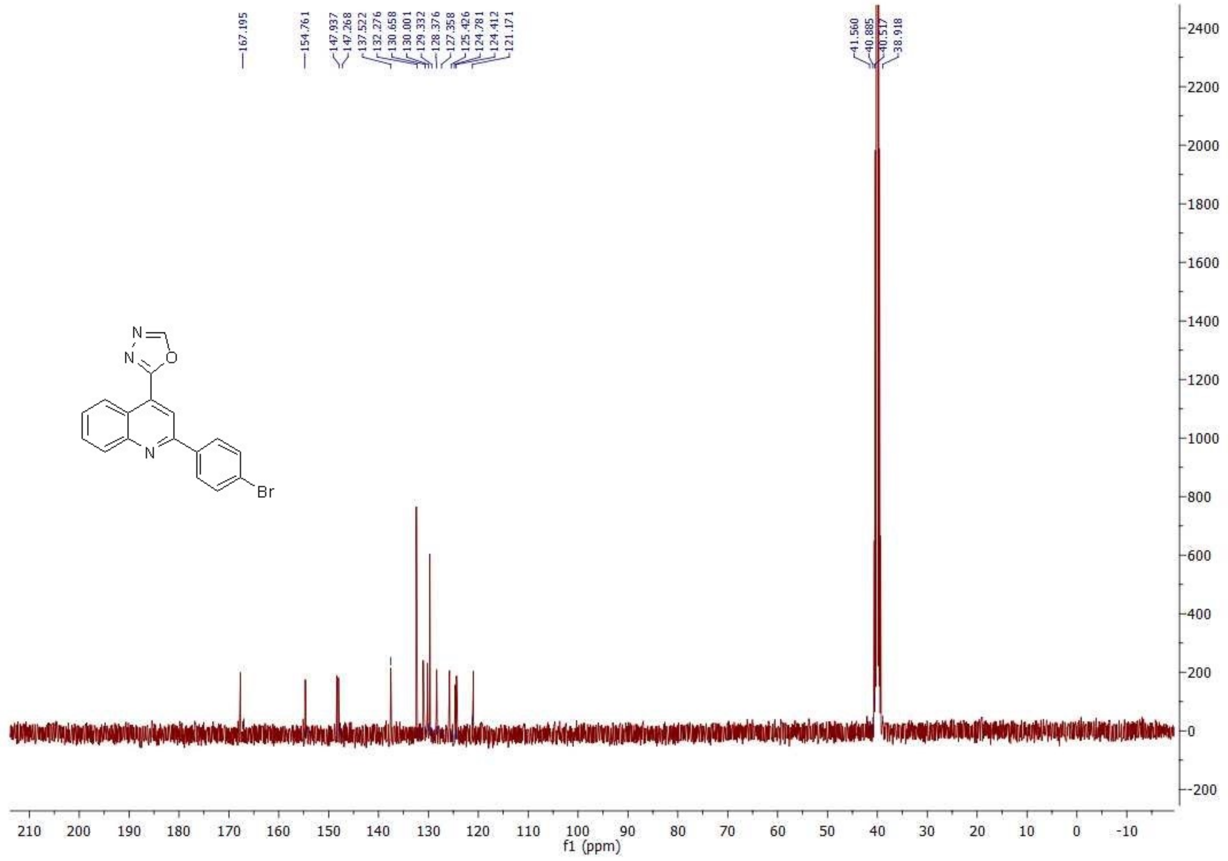

Figure S2: ^{13}CNMR spectrum of the compound **7**.

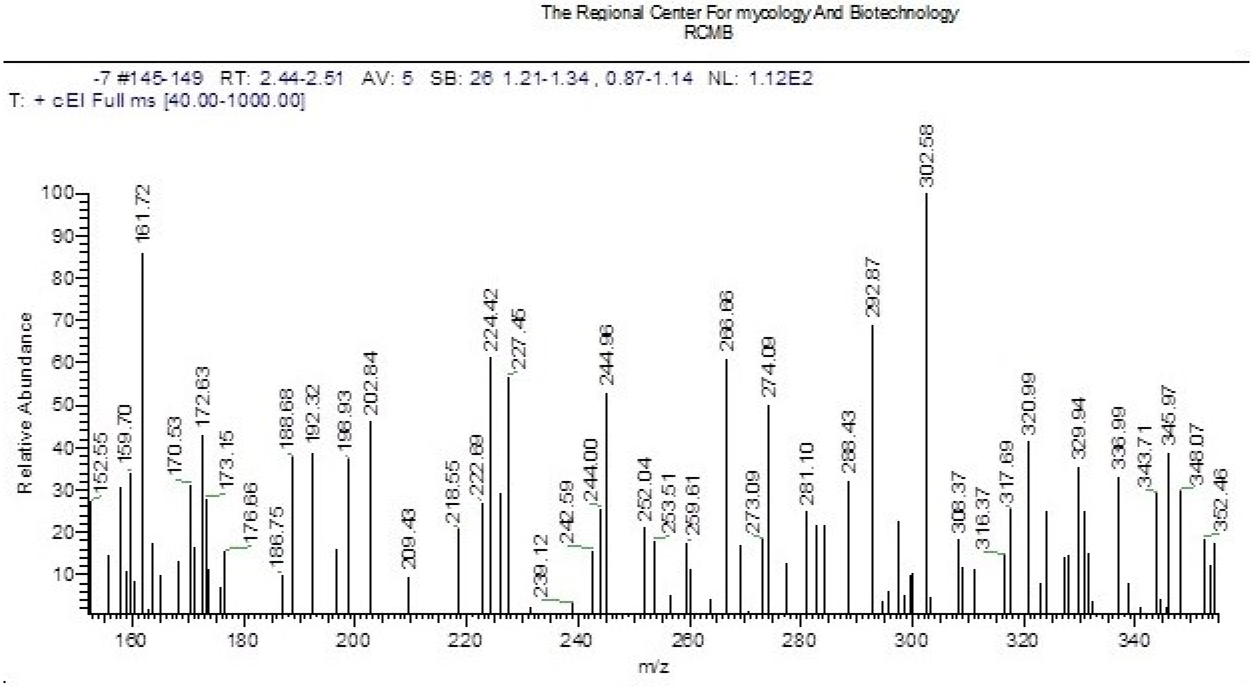

Figure S3: MS of the compound **7**.

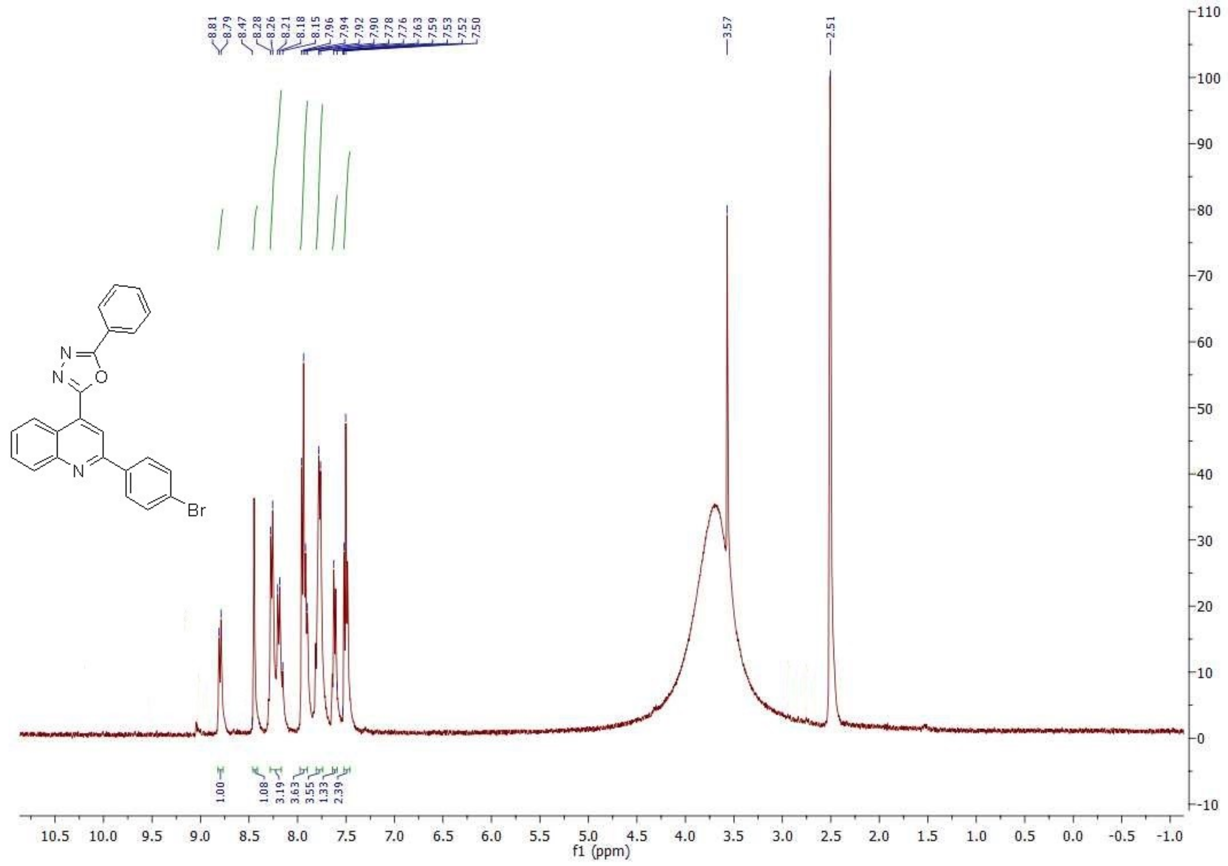

Figure S4: ^{1}HNMR spectrum of the compound **8a**.

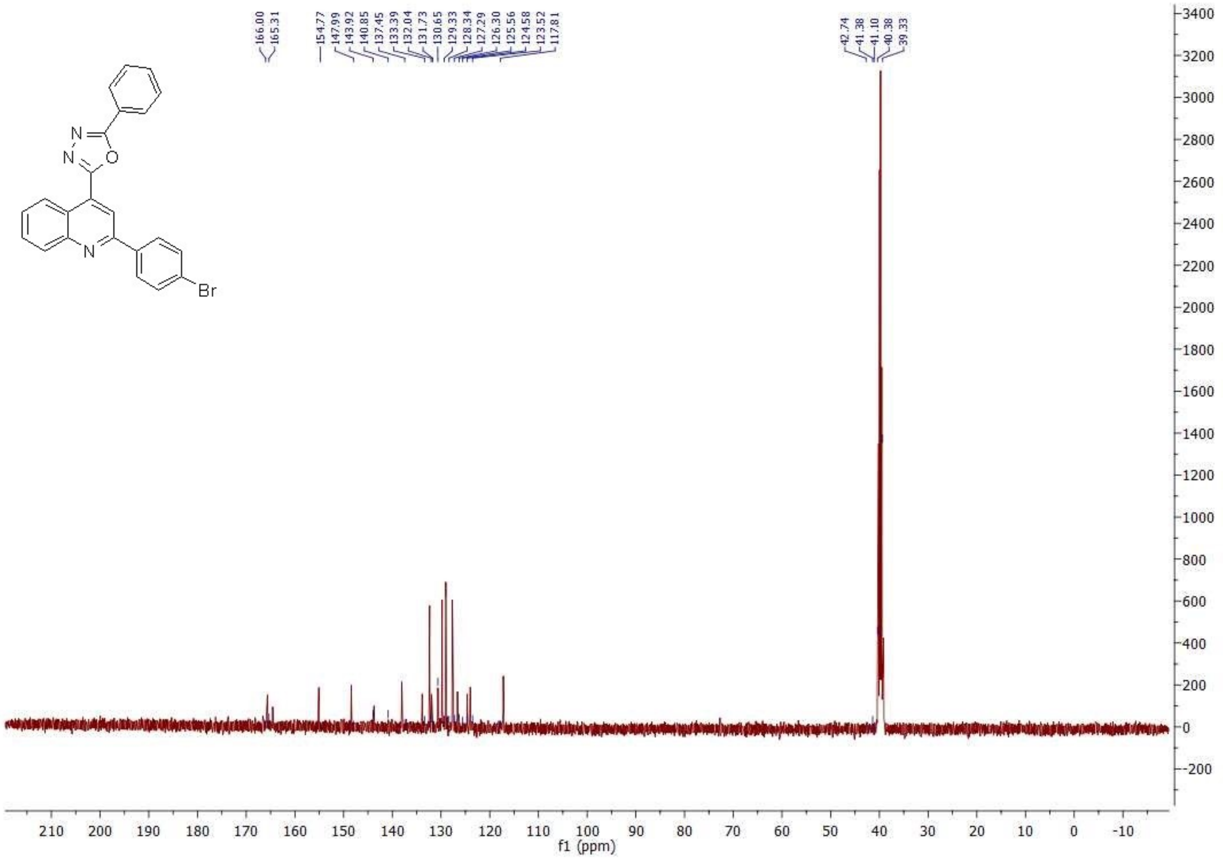

Figure S5: ^{13}CNMR spectrum of the compound **8a**.

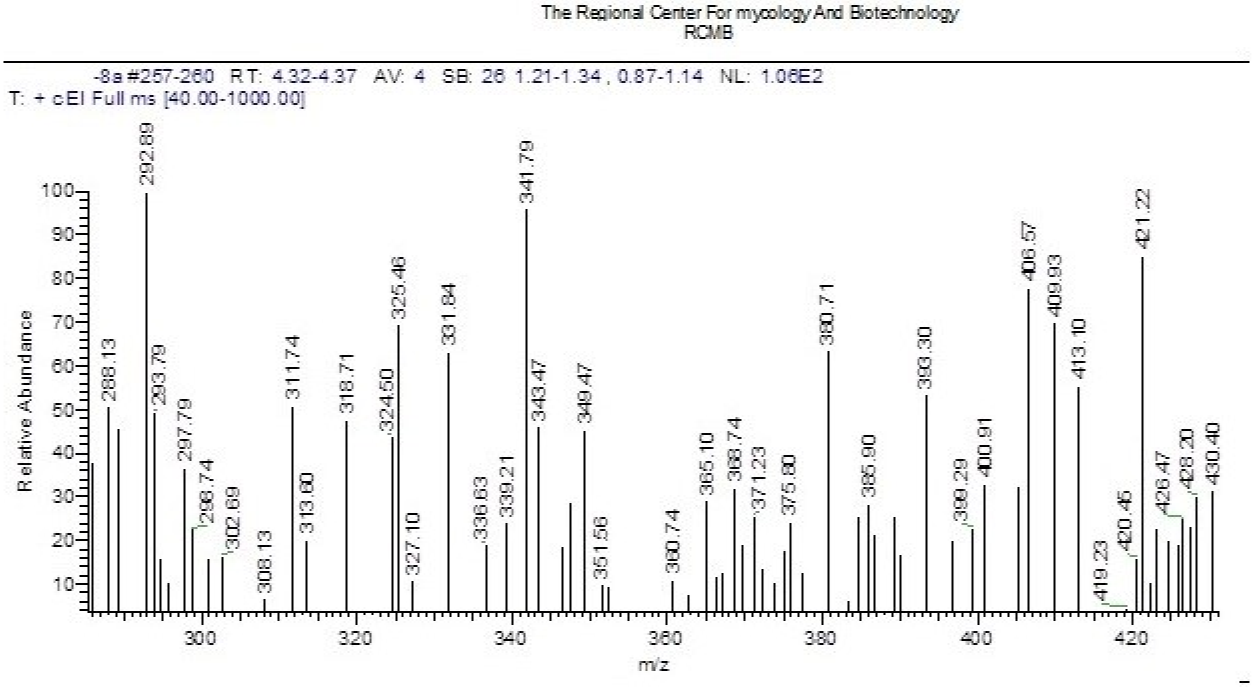

Figure S6: MS of the compound **8a**.

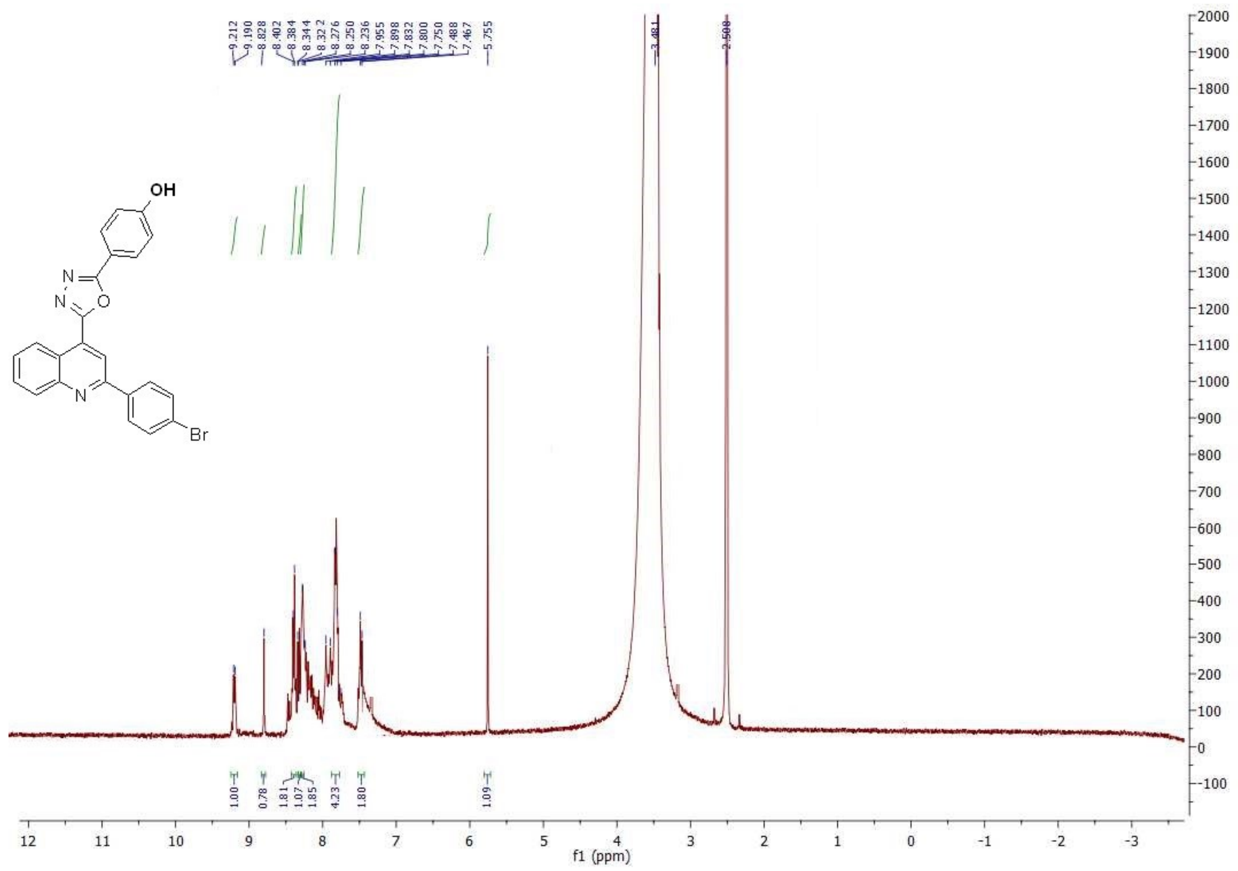

Figure S7: ^{1}HNMR spectrum of the compound **8b**.

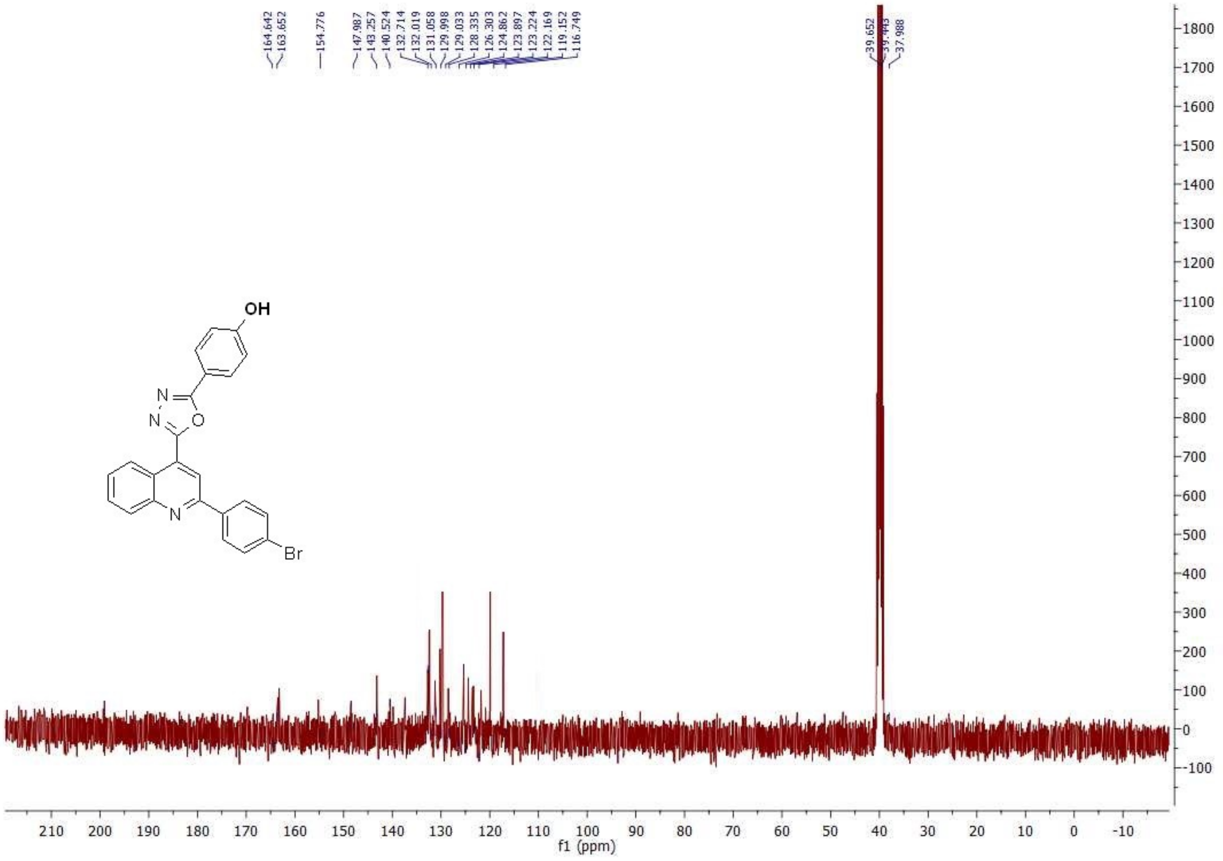

Figure S8: ^{13}CNMR spectrum of the compound **8b**.

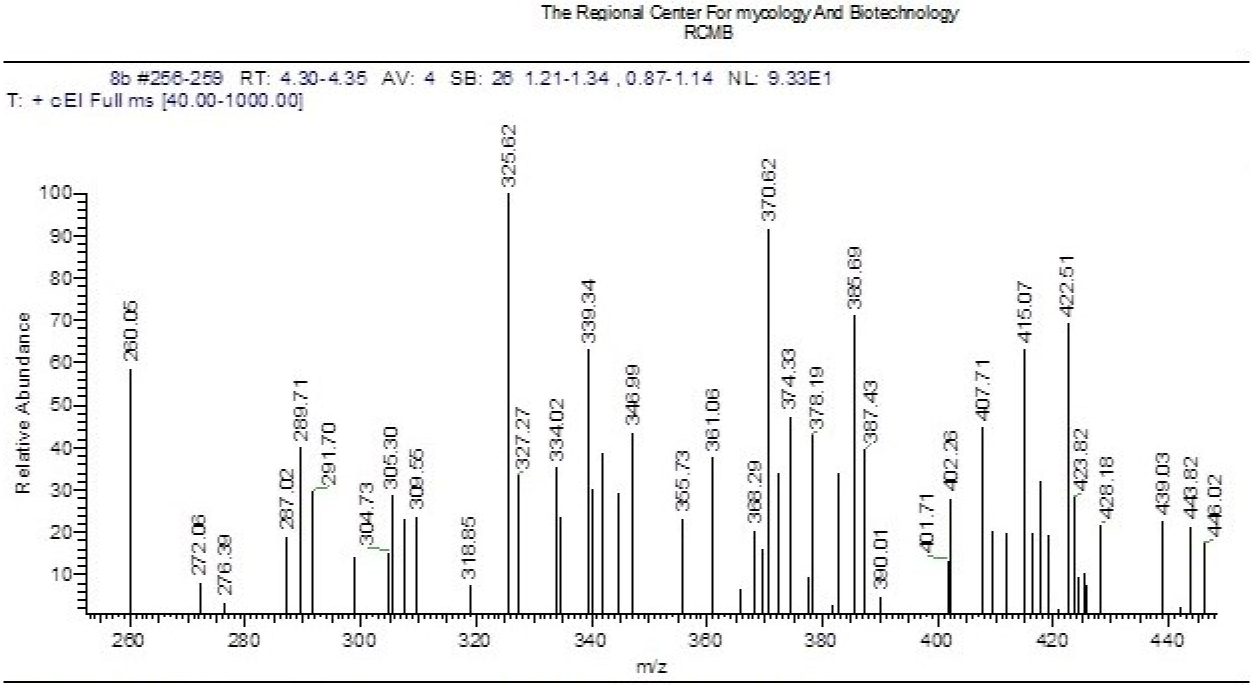

Figure S9: MS of the compound **8b**.

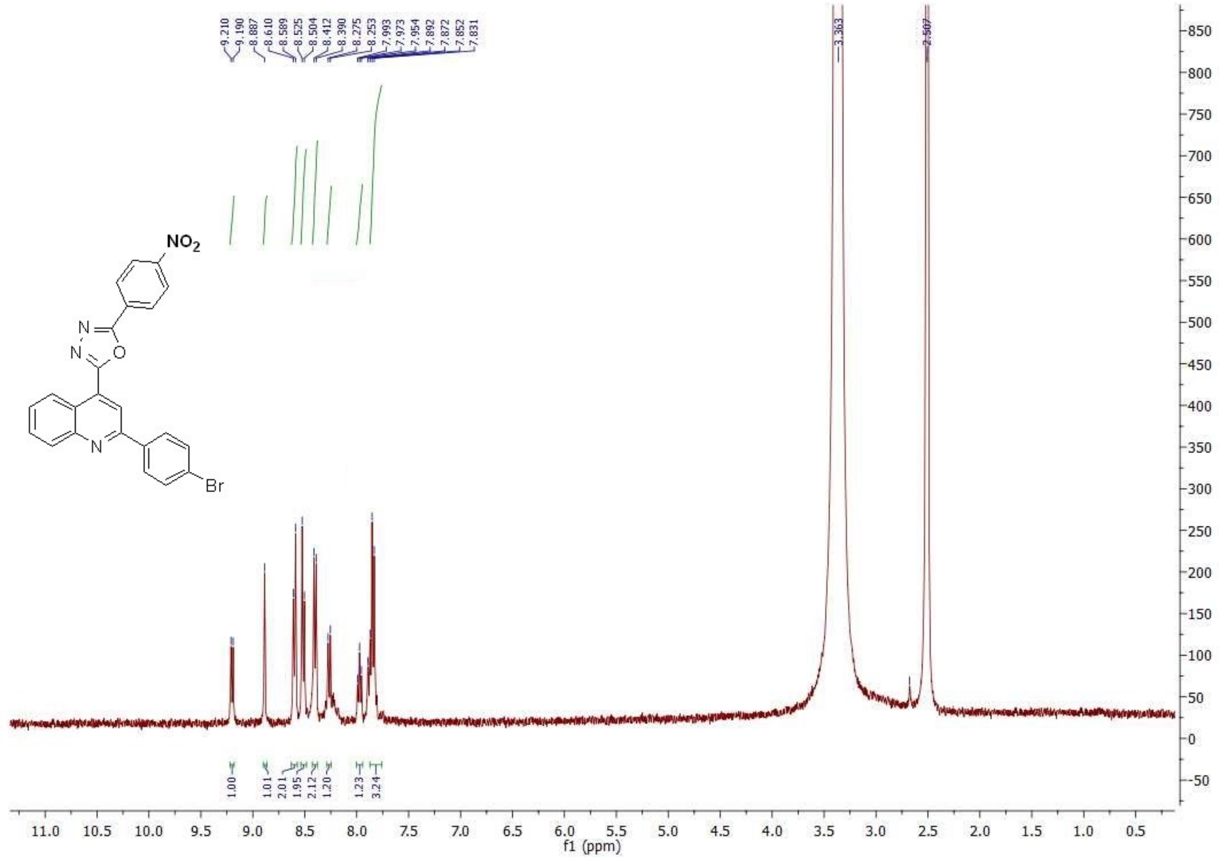

Figure S10: ^{1}HNMR spectrum of the compound **8c**.

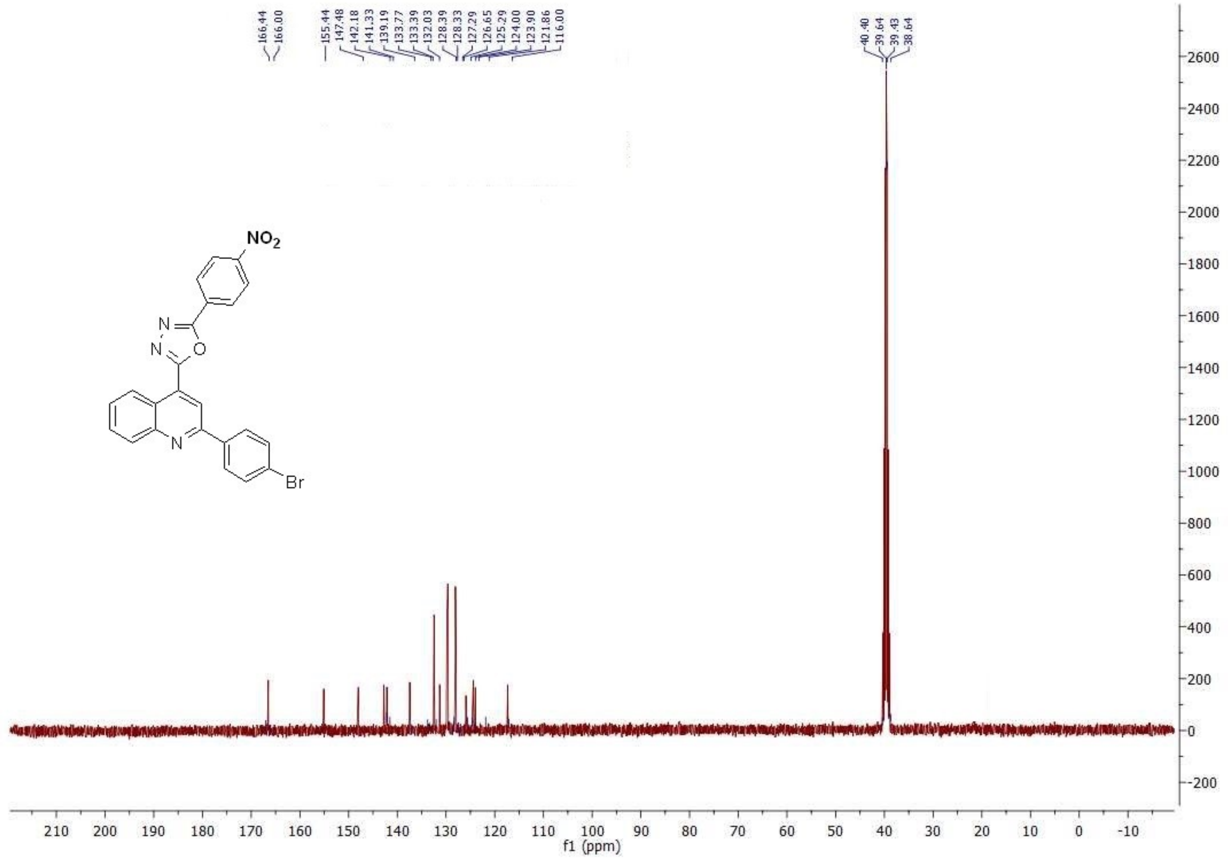

Figure S11: ^{13}CNMR spectrum of the compound **8c**.

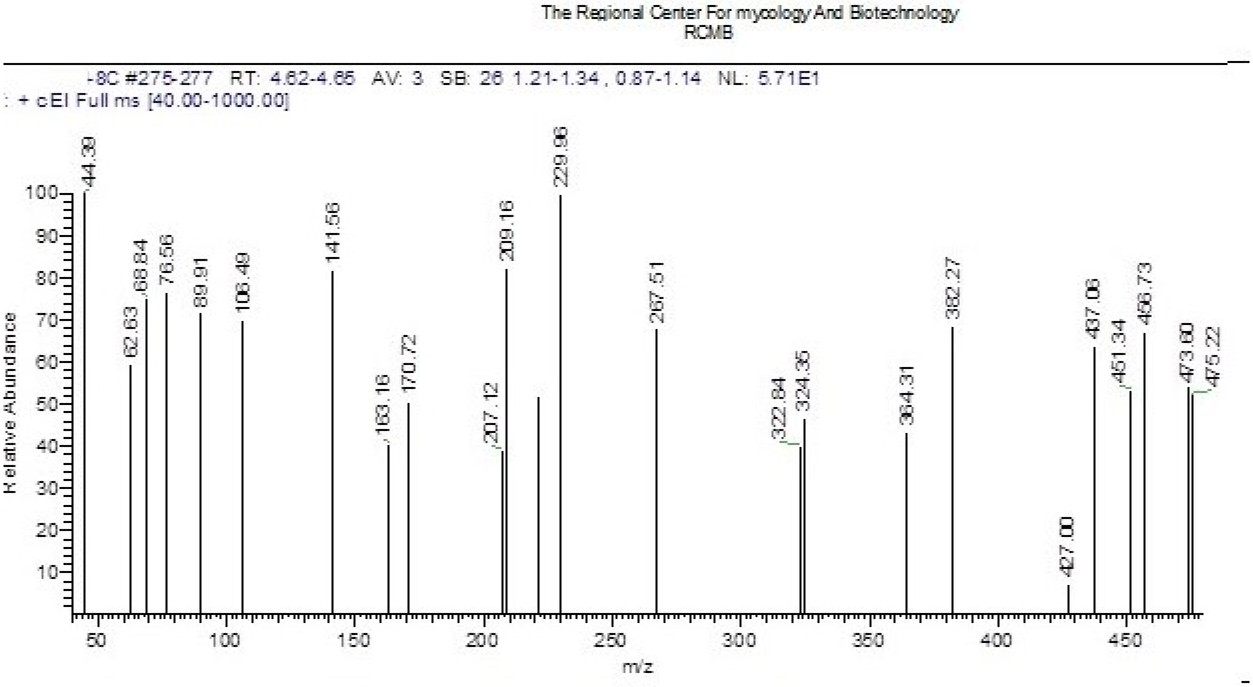

Figure S12: MS of the compound **8c**.

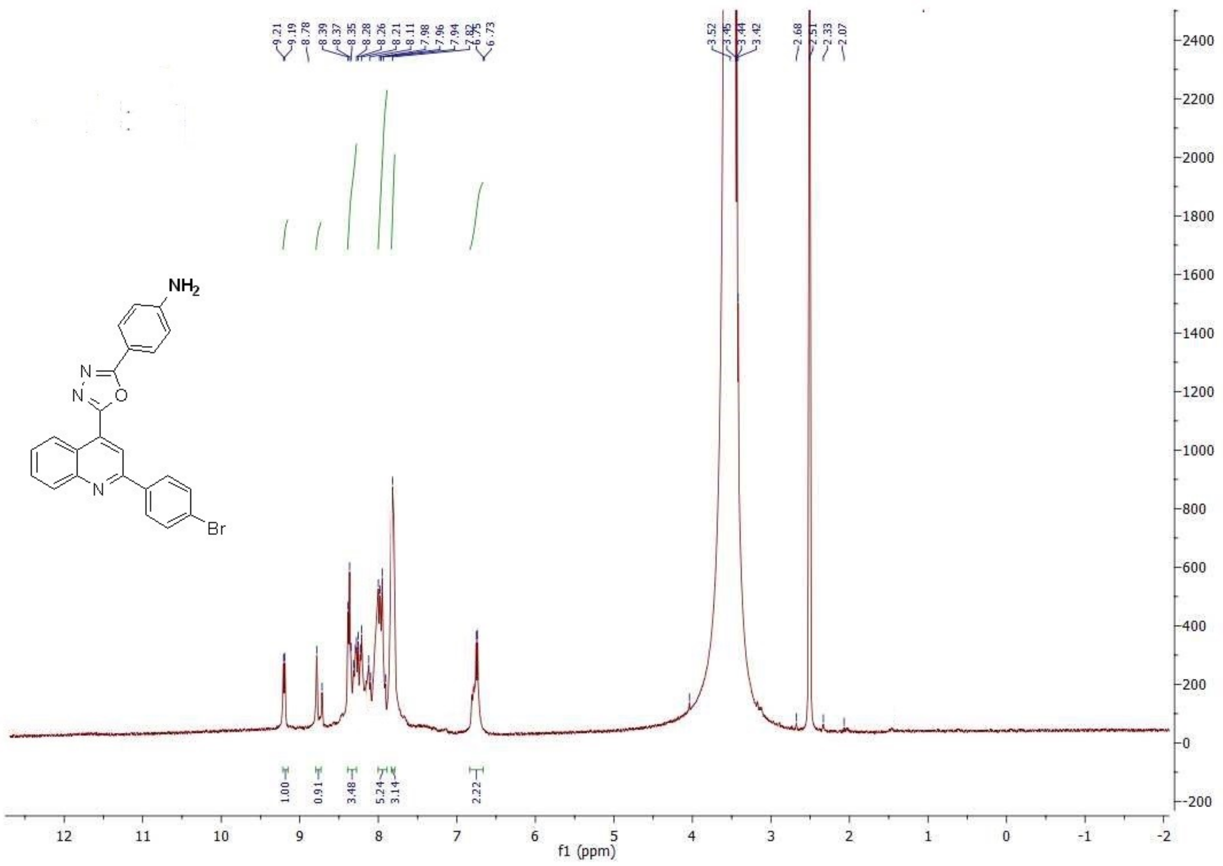

Figure S13: ^{1}HNMR spectrum of the compounds **8d**.

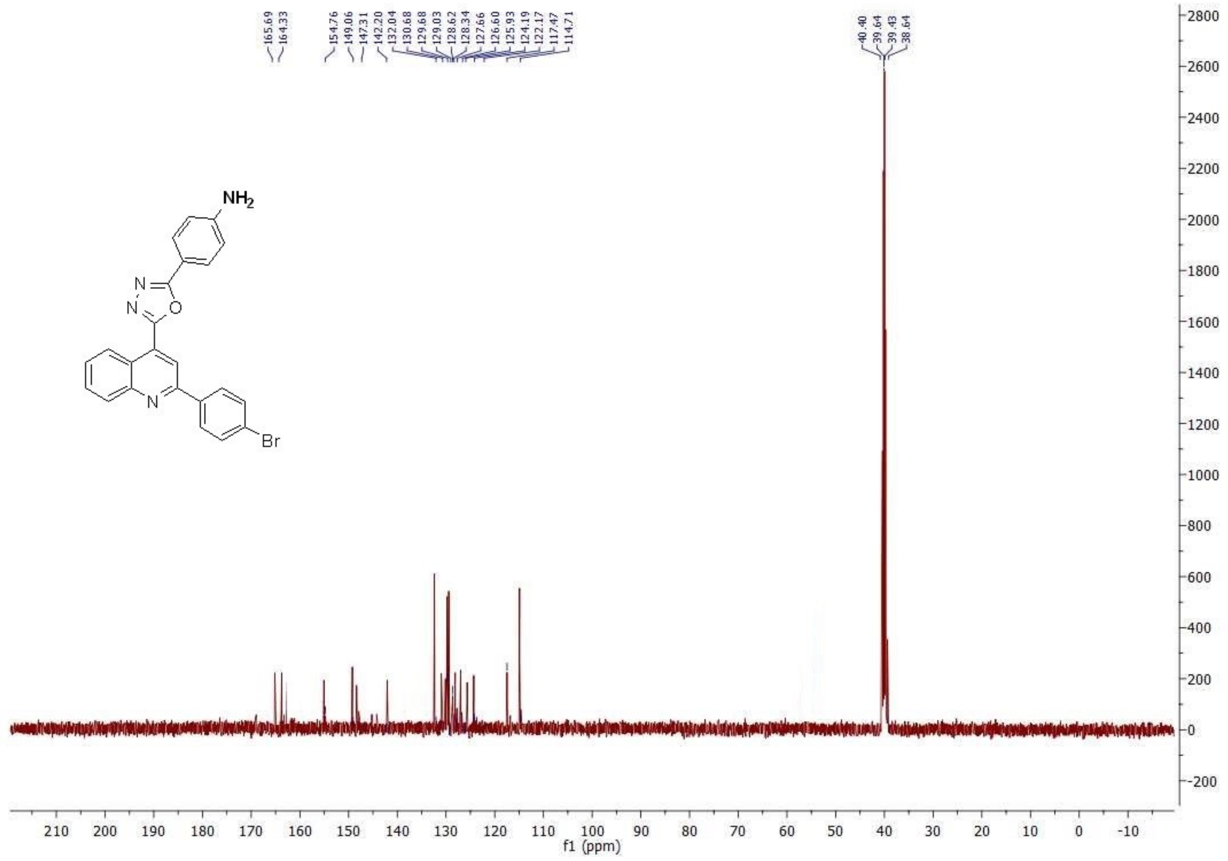

Figure S14: ^{13}CNMR spectrum of the compound **8d**.

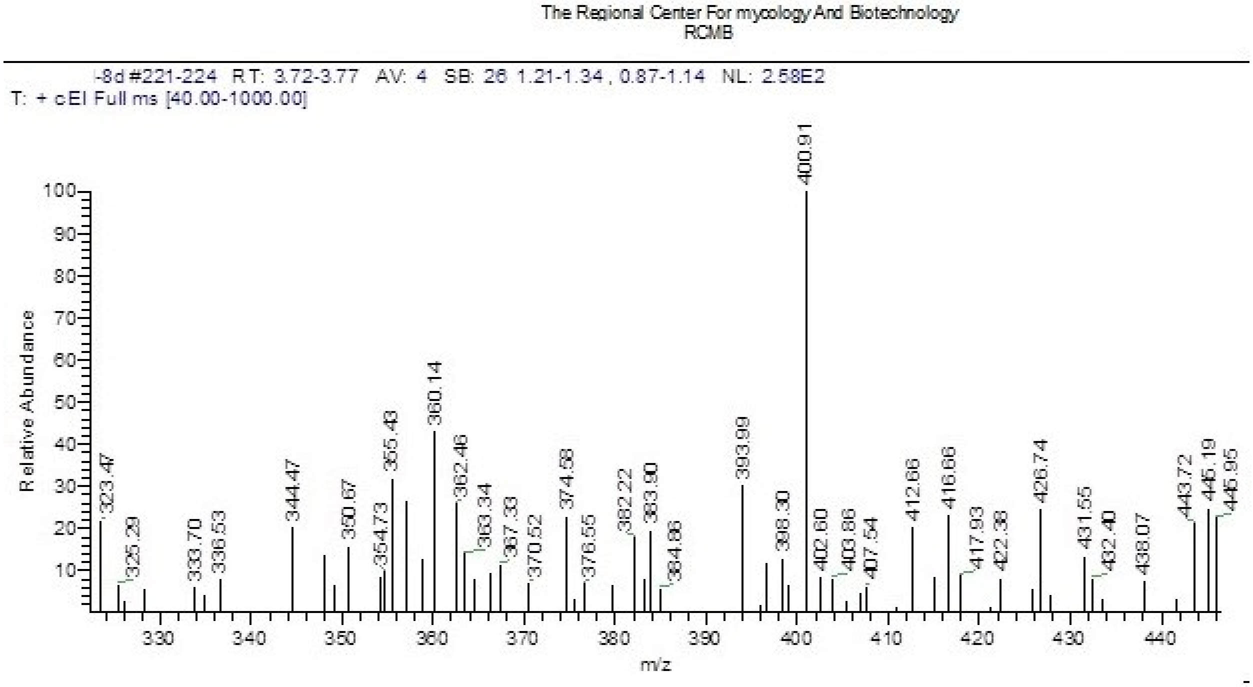

Figure S15: MS of the compound **8d**.

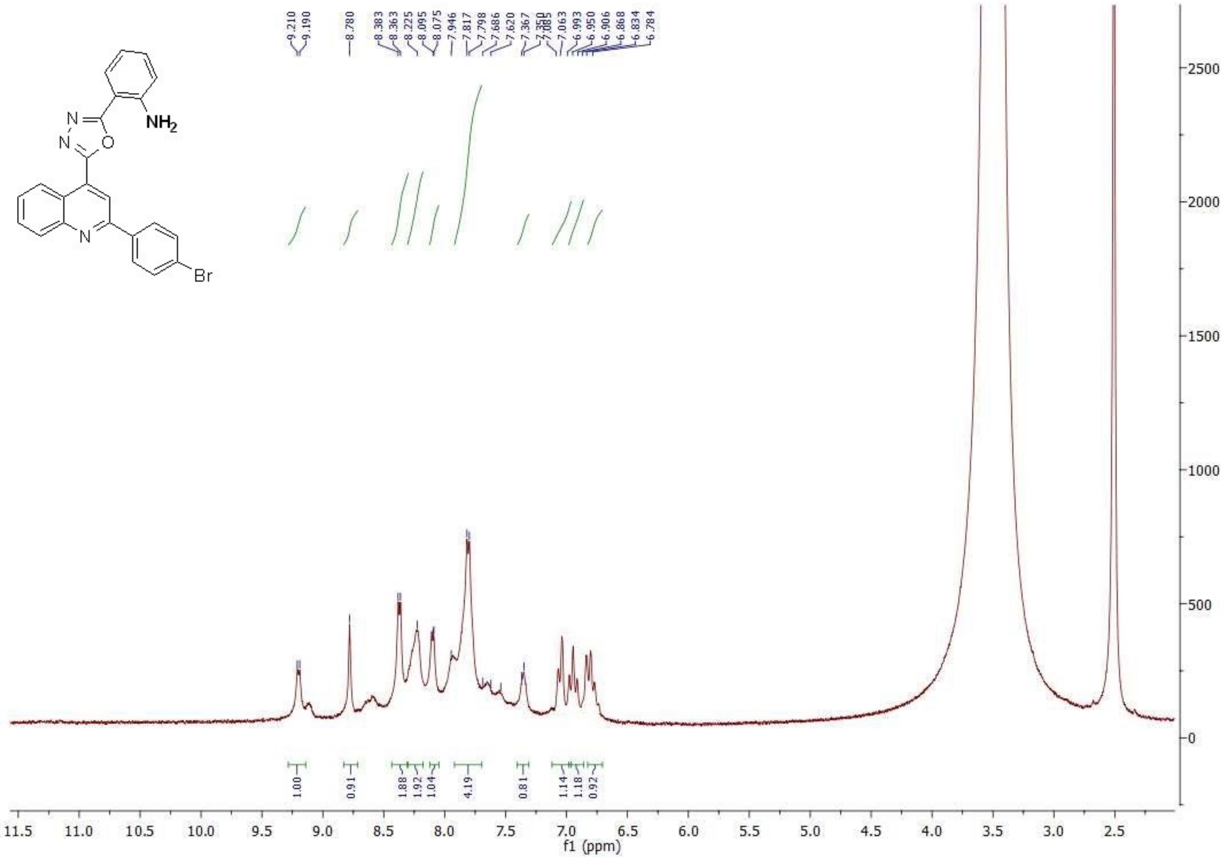

Figure S16: ^{1}HNMR spectrum of the compound **8e**.

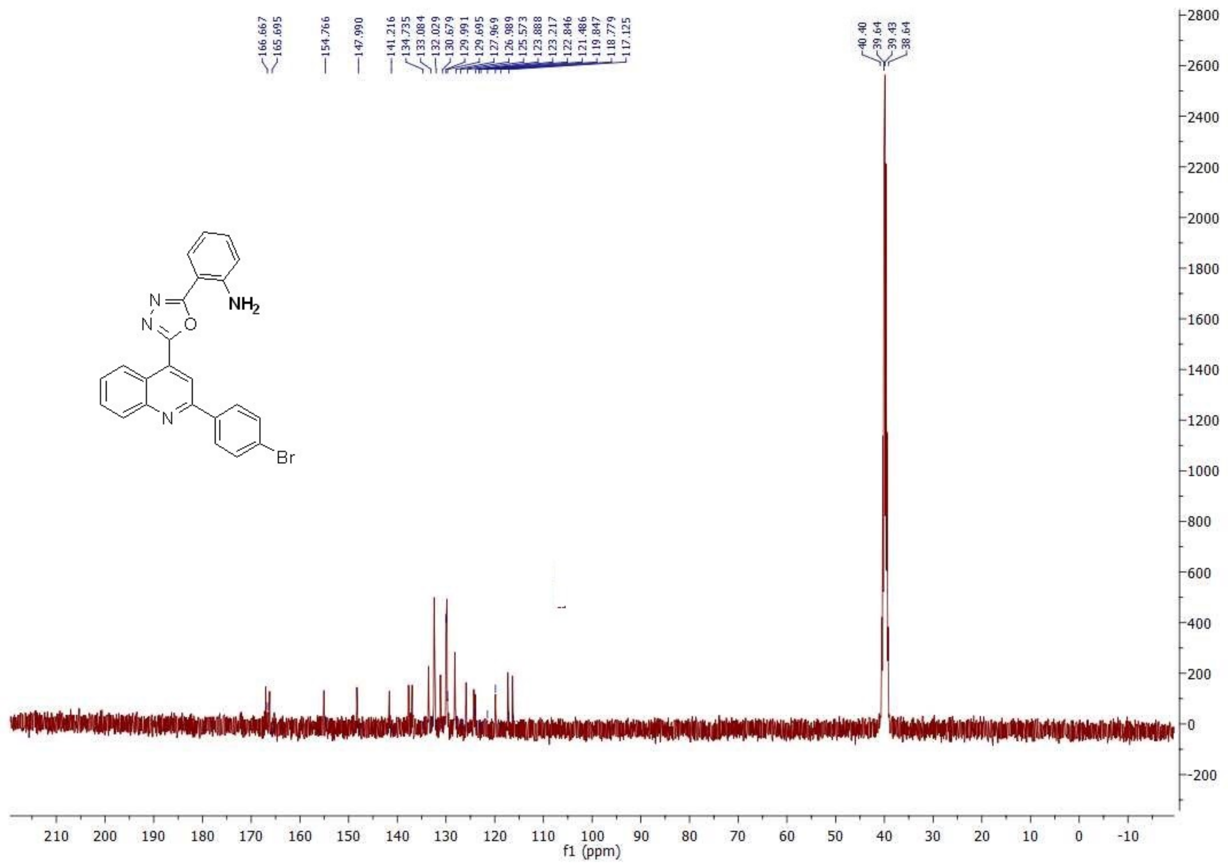

Figure S17: ^{13}CNMR spectrum of the compound **8e**.

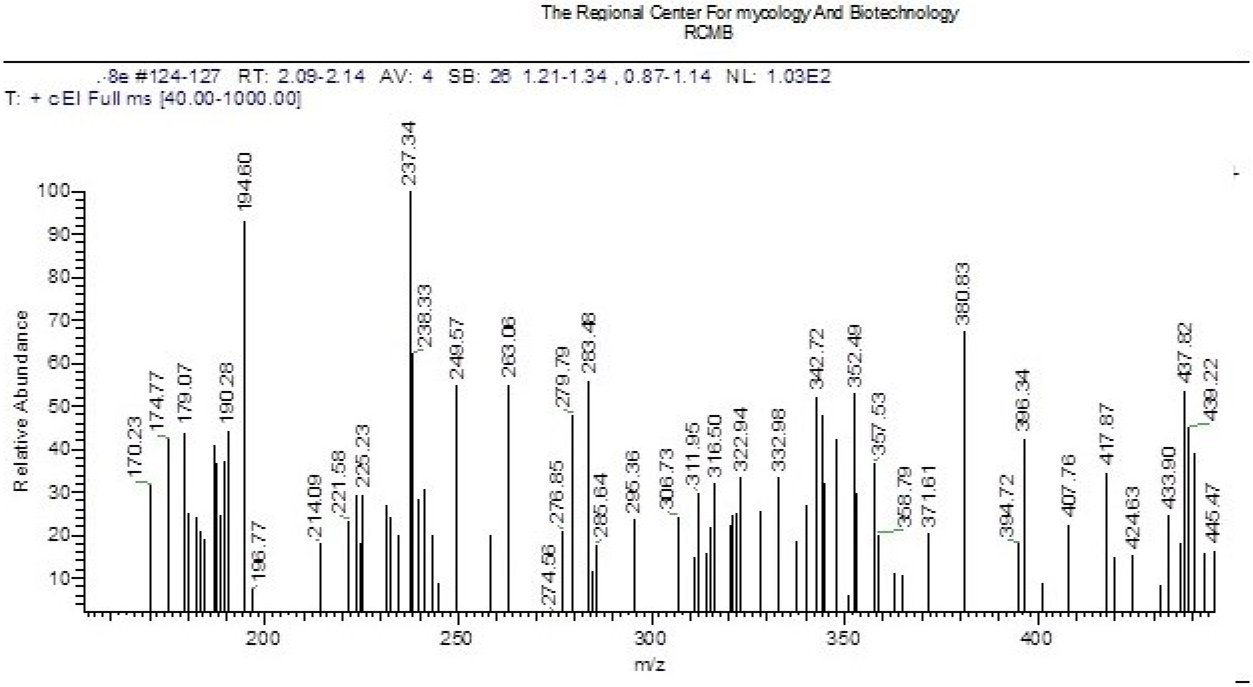

Figure S18: MS of the compound **8e**.

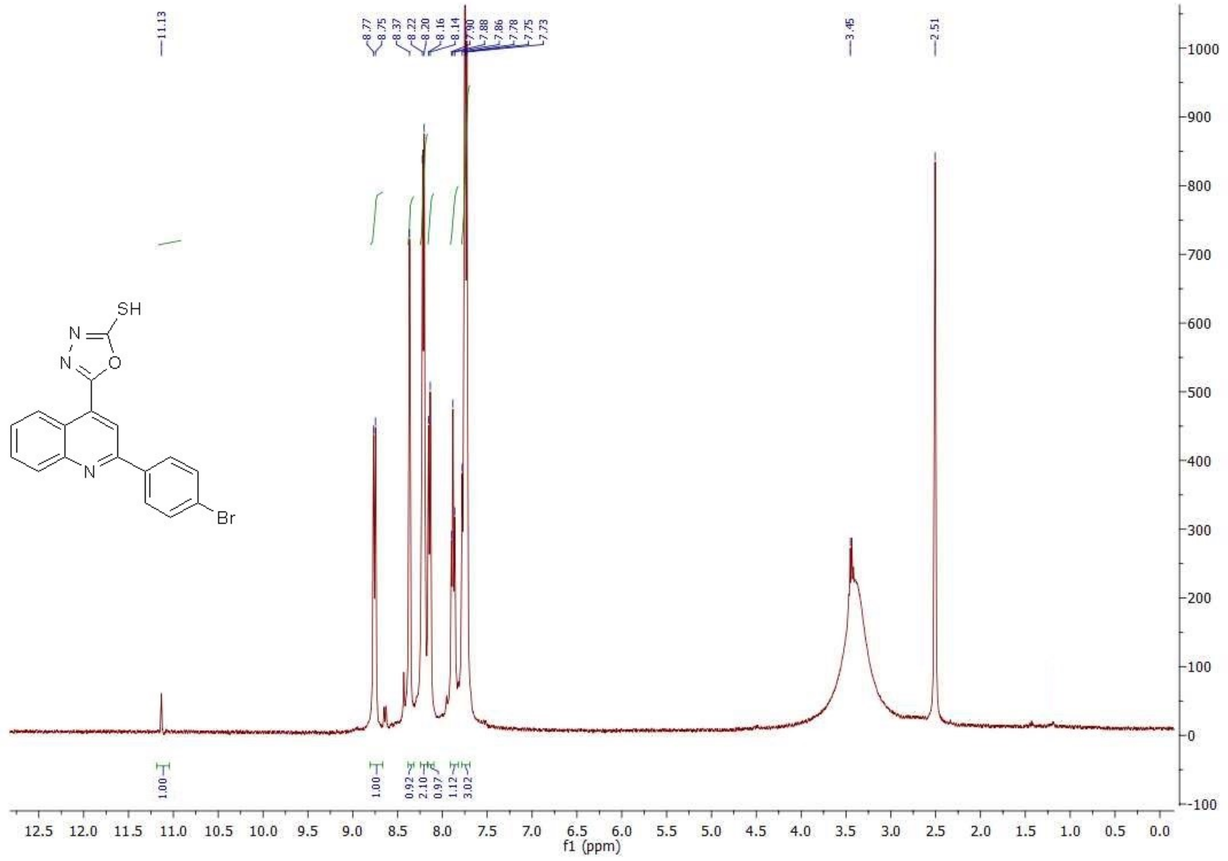

Figure S19: ^{1}HNMR spectrum of the compound **9**.

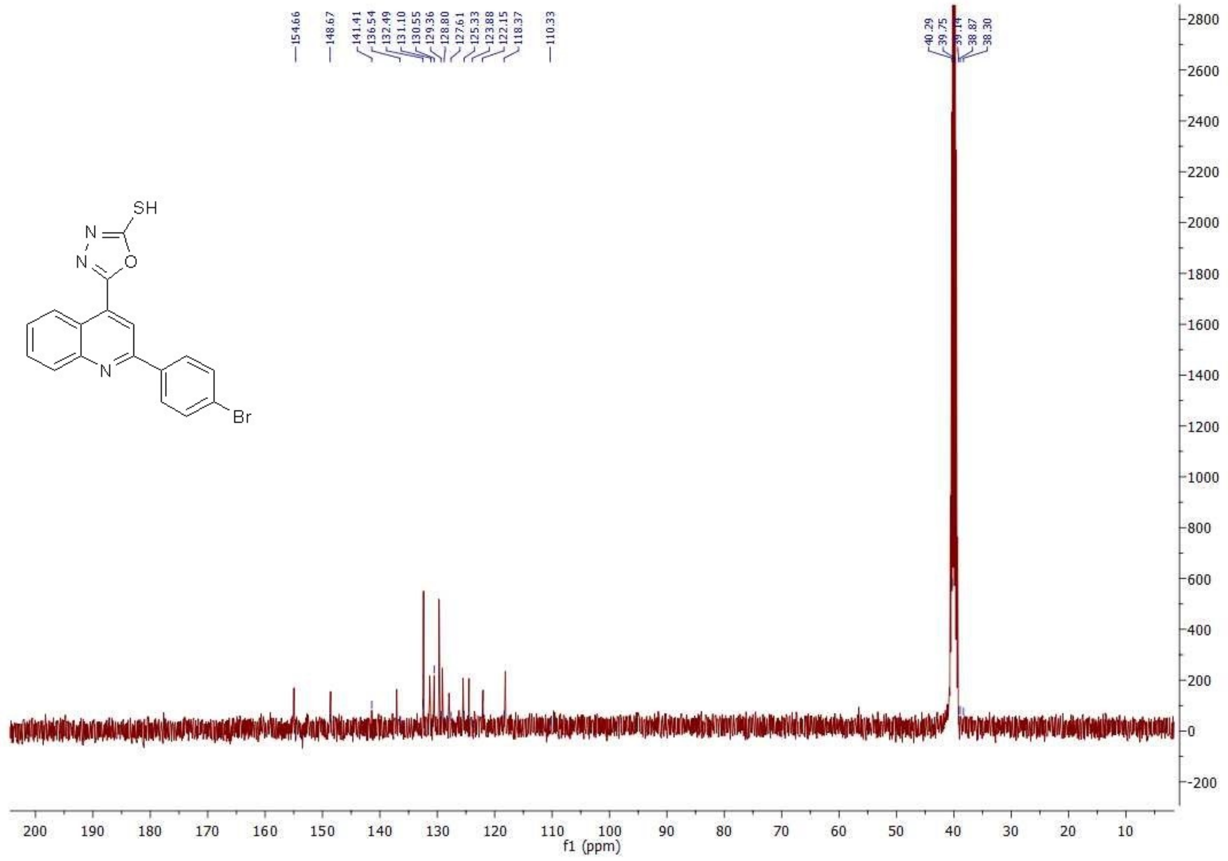

Figure S20: ^{13}CNMR spectrum of the compound **9**.

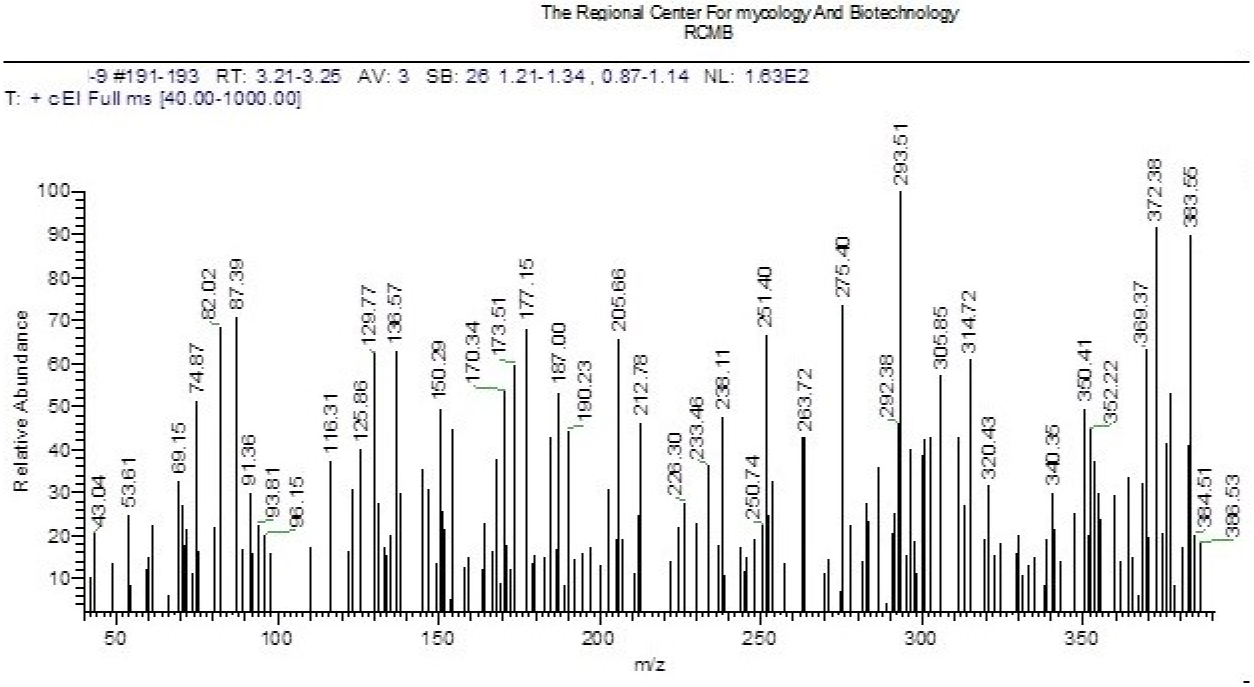

Figure S21: MS of the compound **9.**

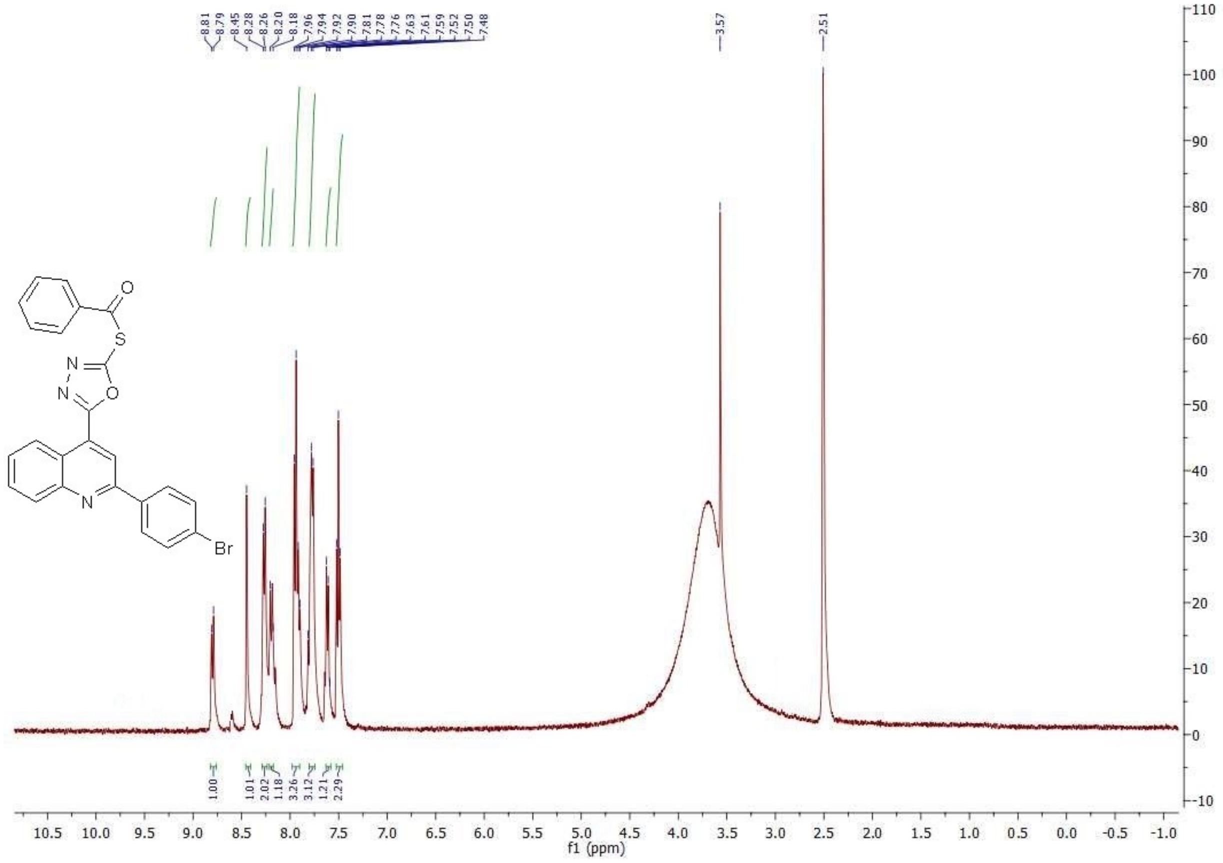

Figure S22: ^{1}HNMR spectrum of the compound **10**.

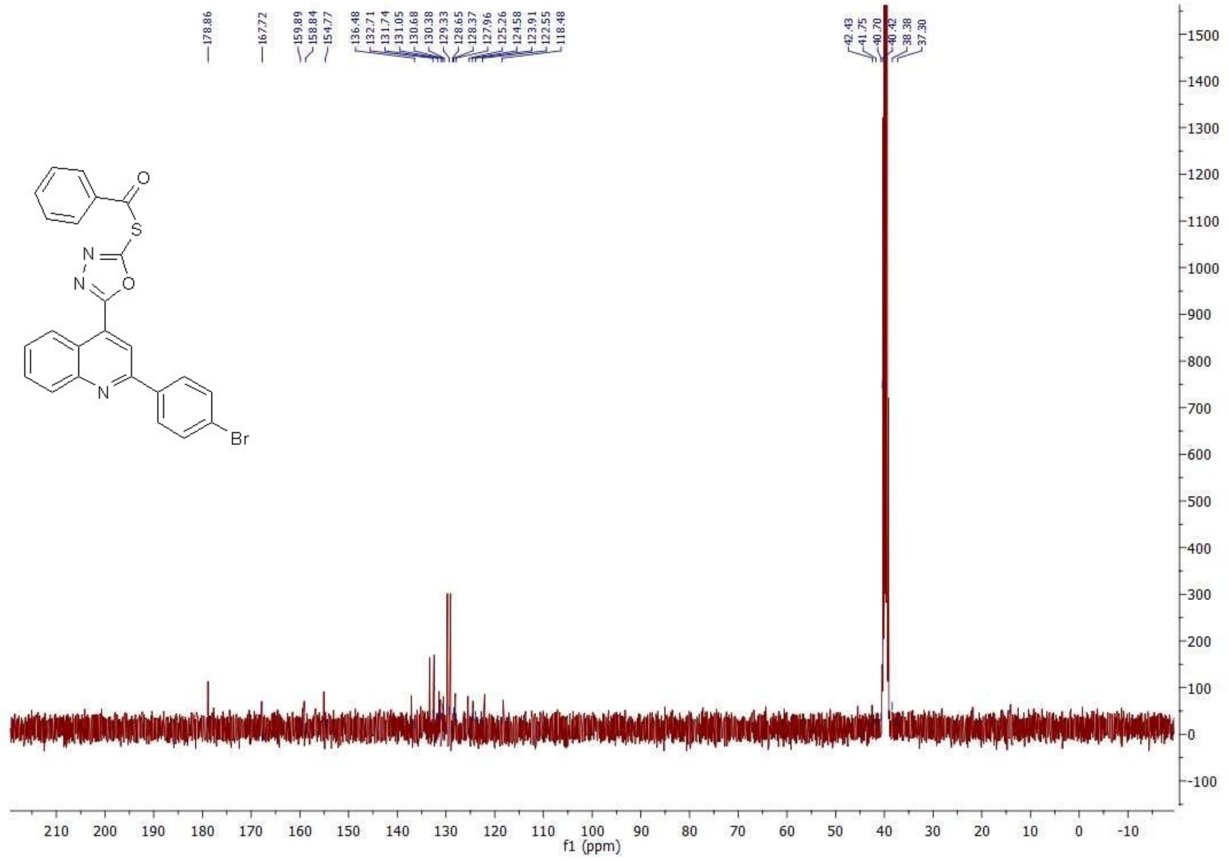

Figure S23: ^{13}CNMR spectrum of the compound **10**.

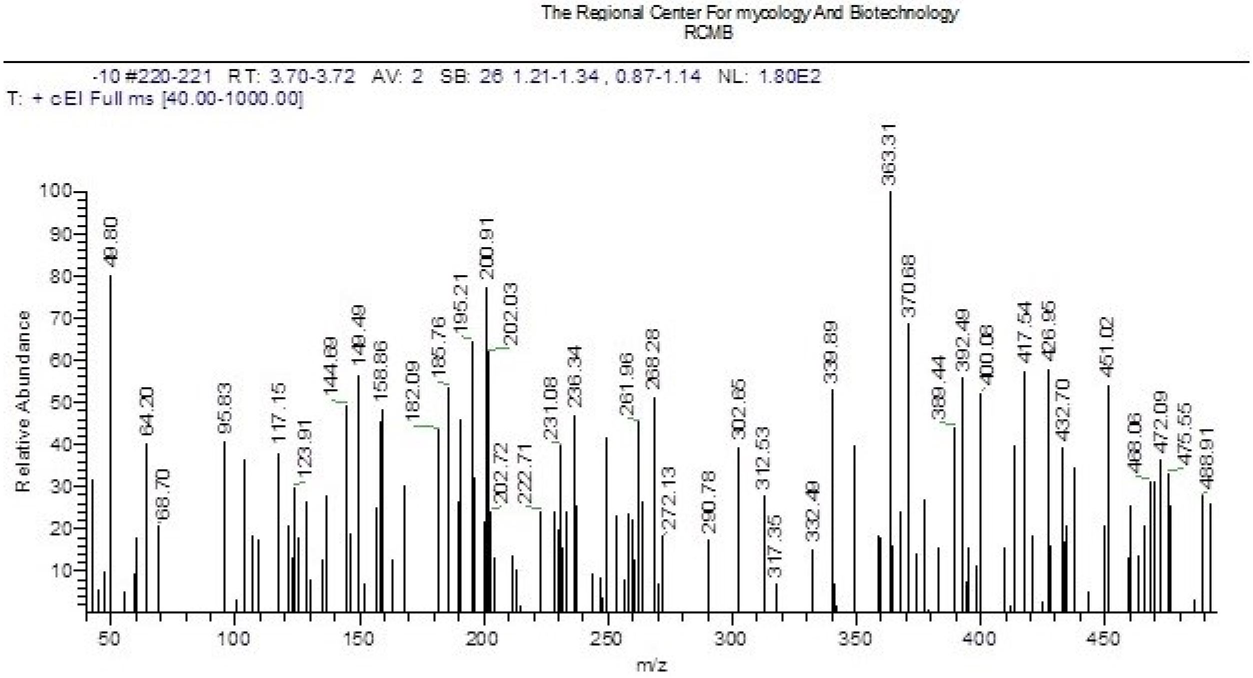

Figure S24: MS of the compound **10**.

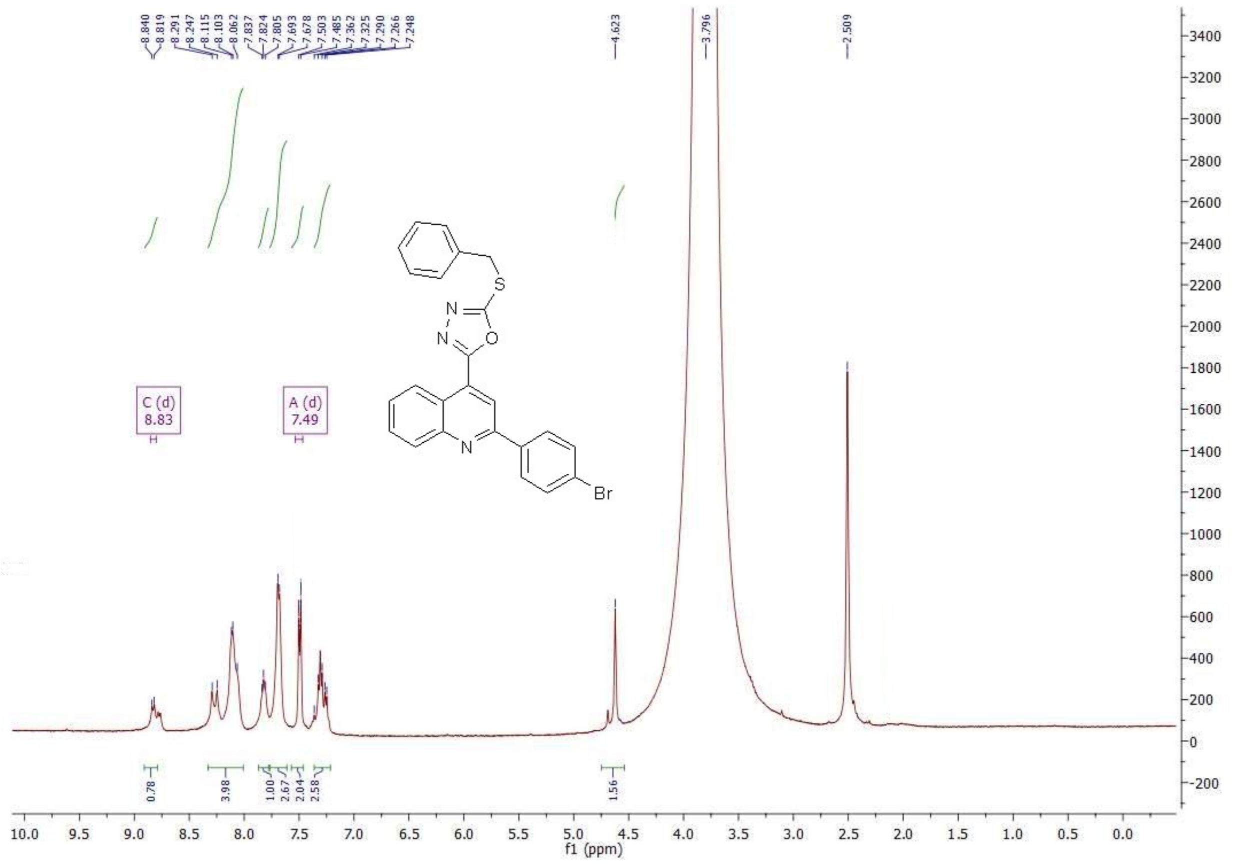

Figure S25: ^{1}HNMR spectrum of the compound **11**.

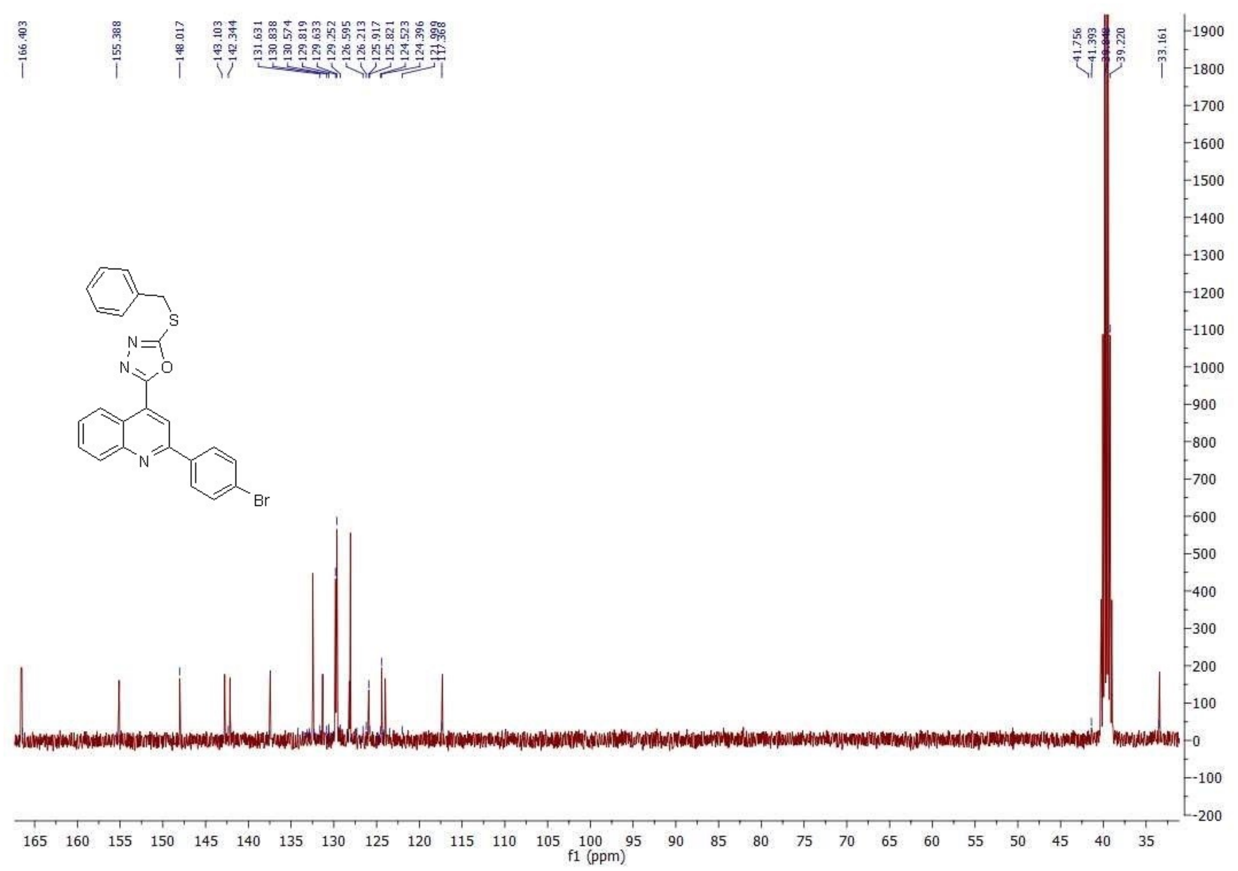

Figure S26: ^{13}CNMR spectrum of the compound **11**.

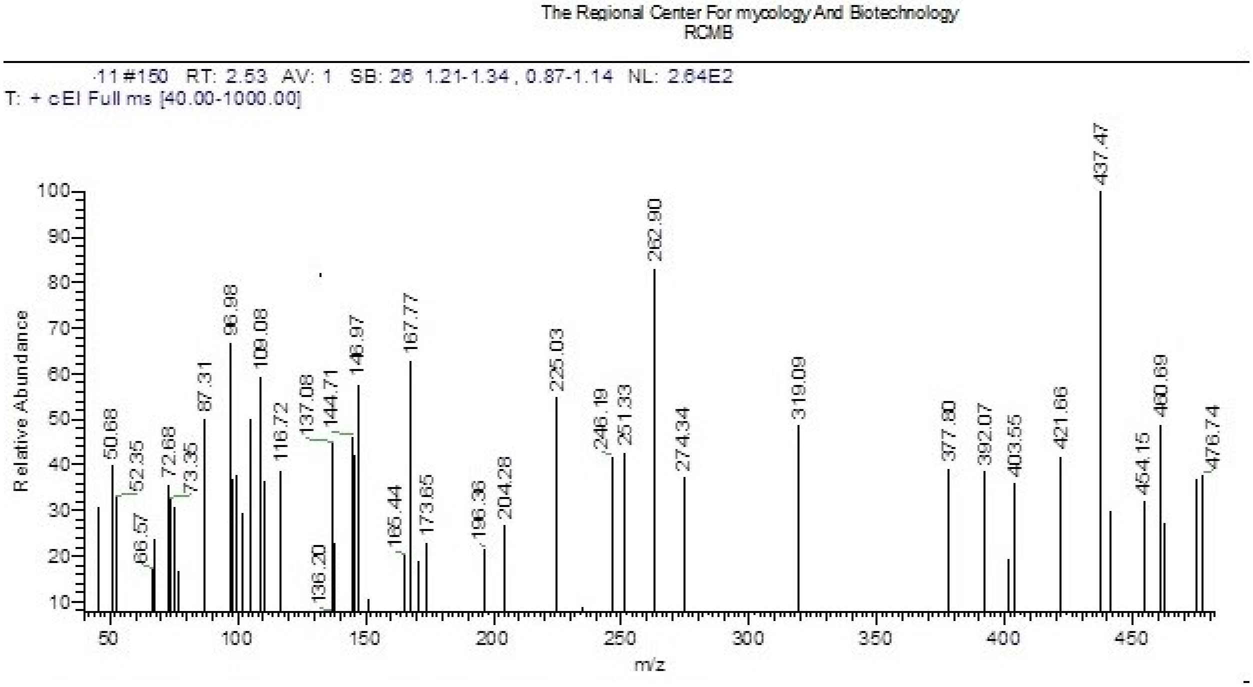

Figure S27: MS of the compound **11**.

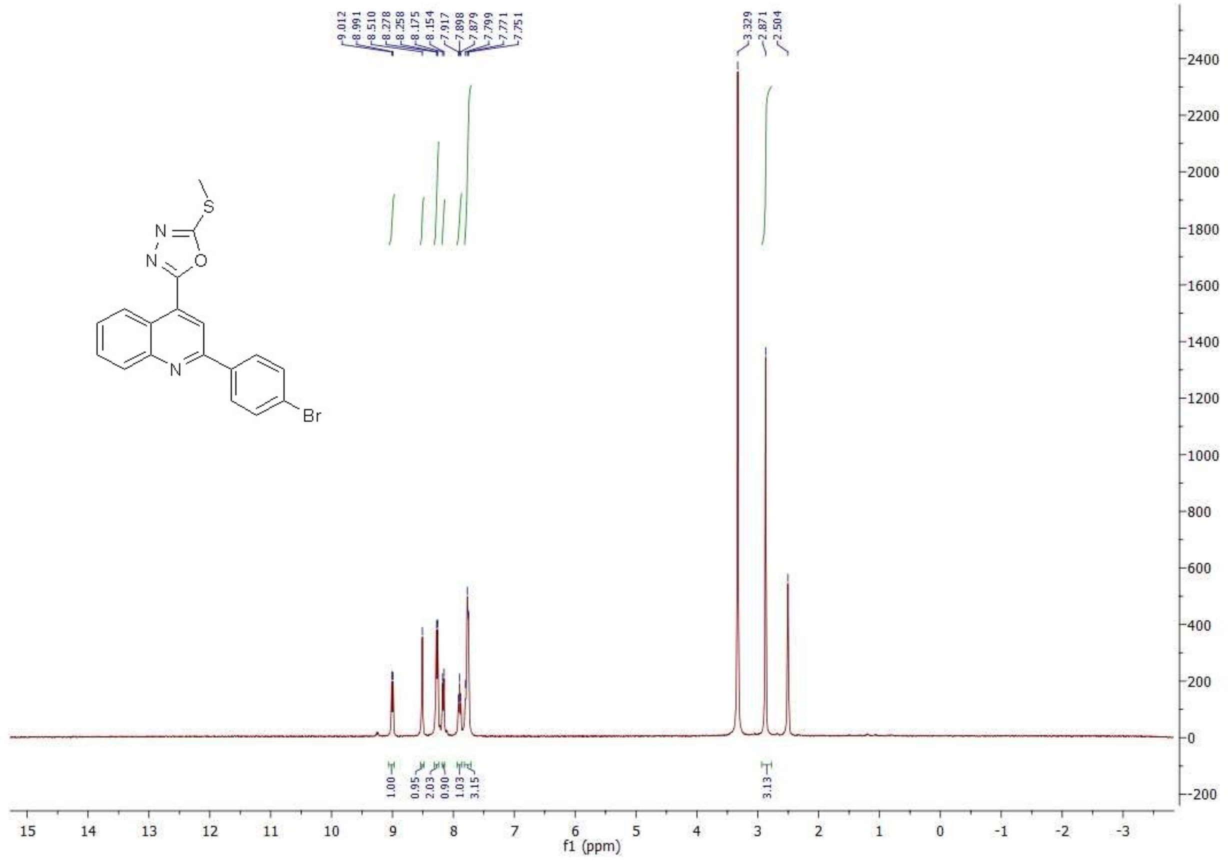

Figure S28: ^{1}HNMR spectrum of the compound **12a**.

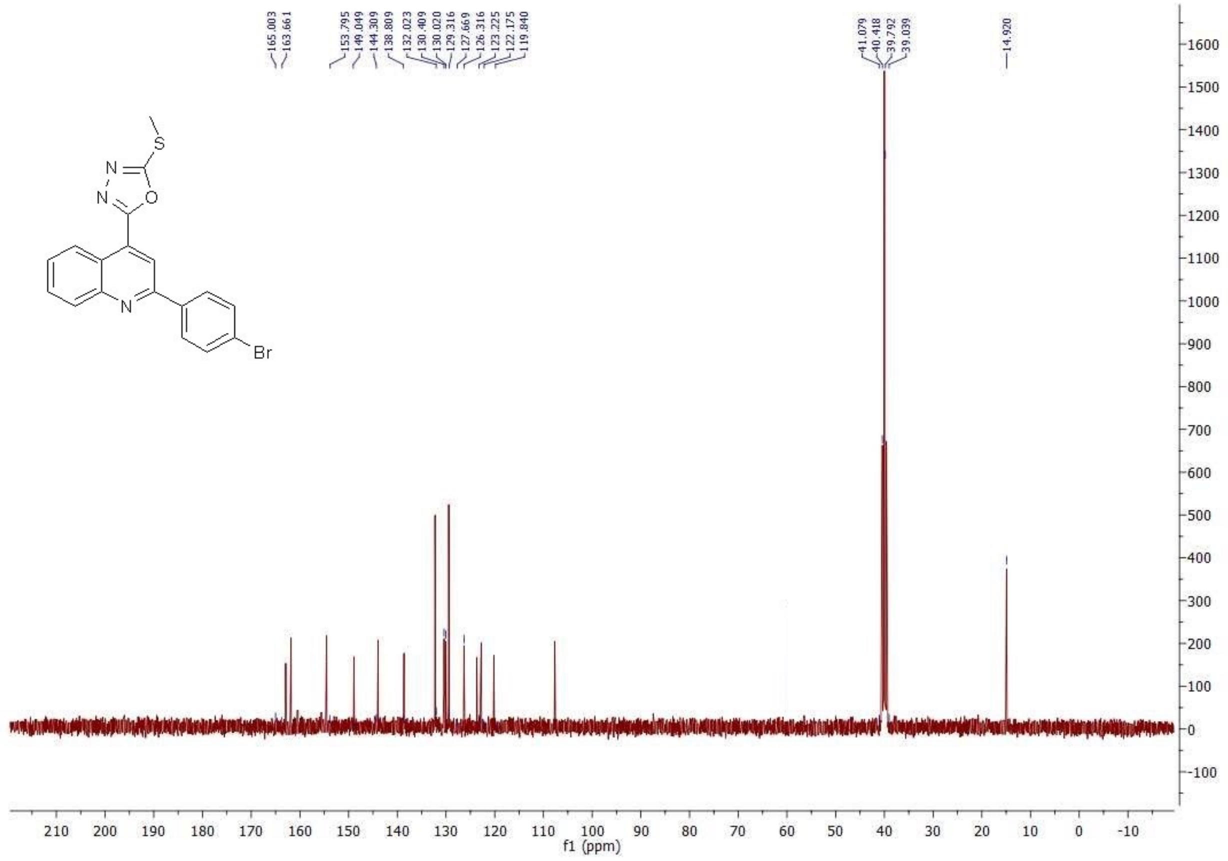

Figure S29: ^{13}CNMR spectrum of the compound **12a**.

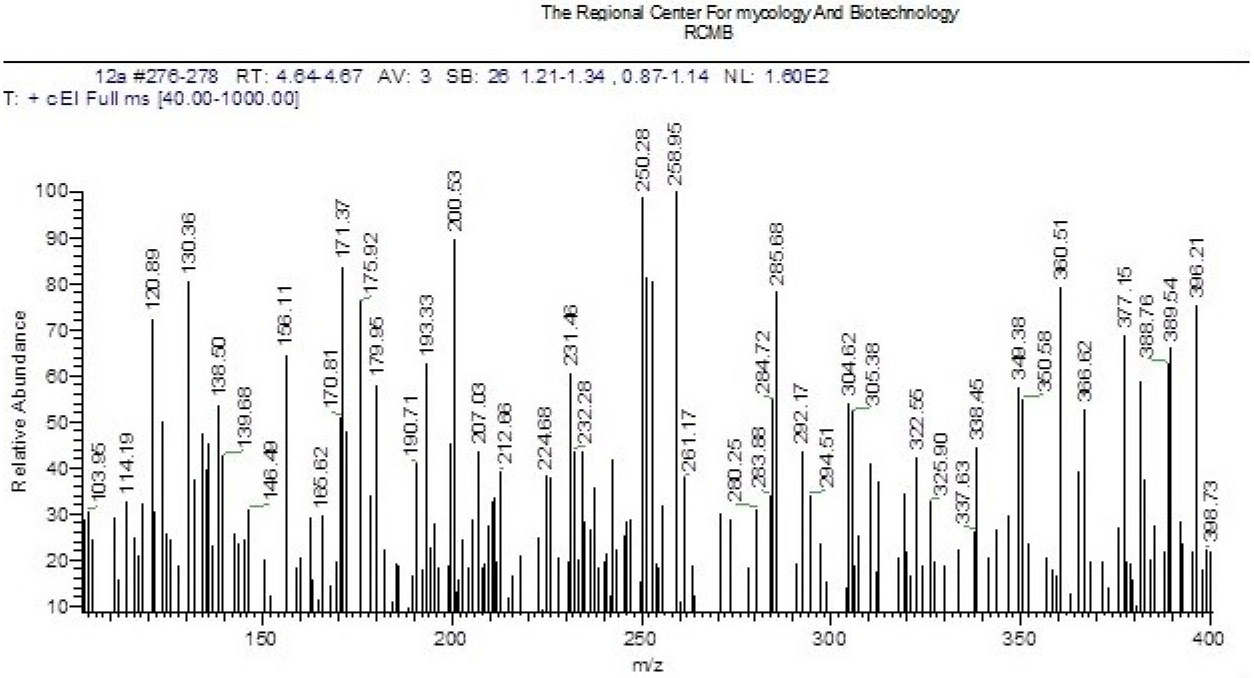

Figure S30: MS of the compound **12a**.

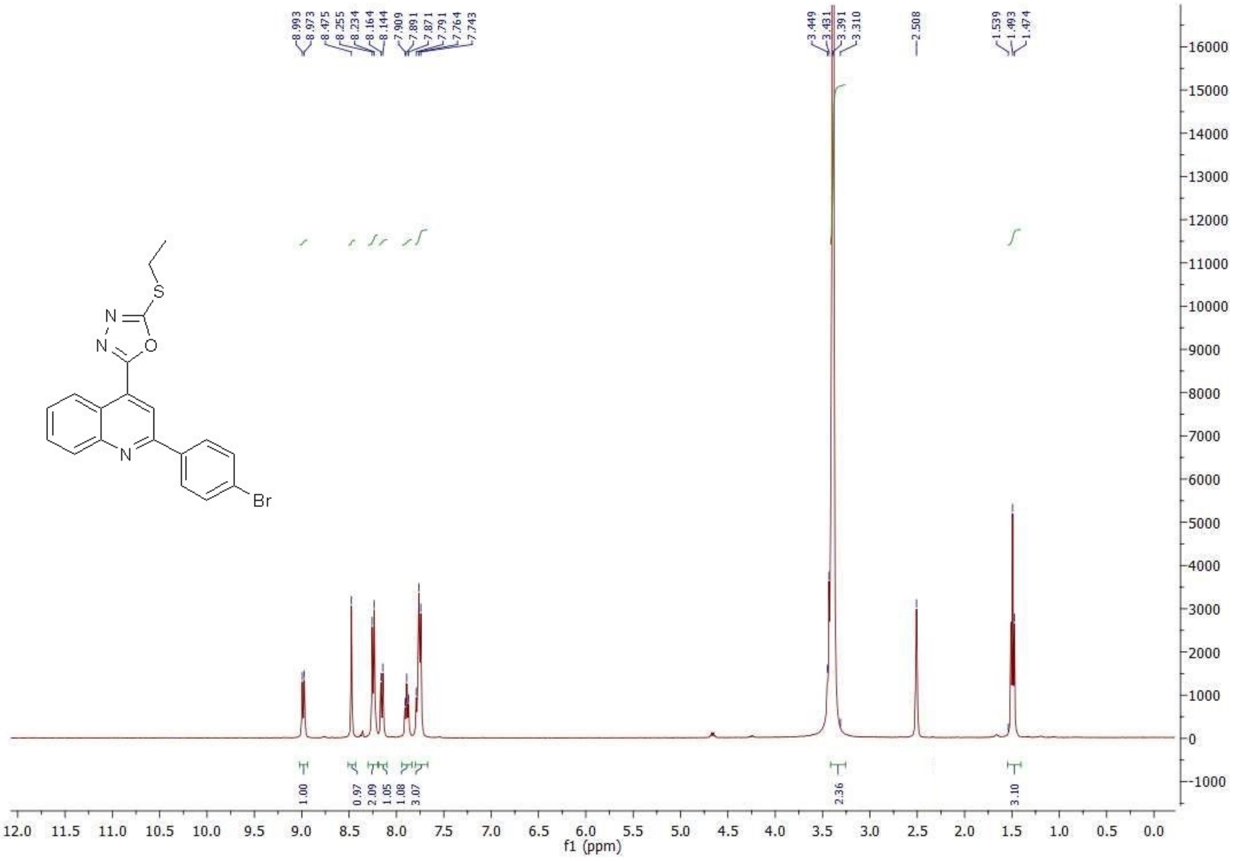

Figure S31: ^{1}HNMR spectrum of the compound **12b**.

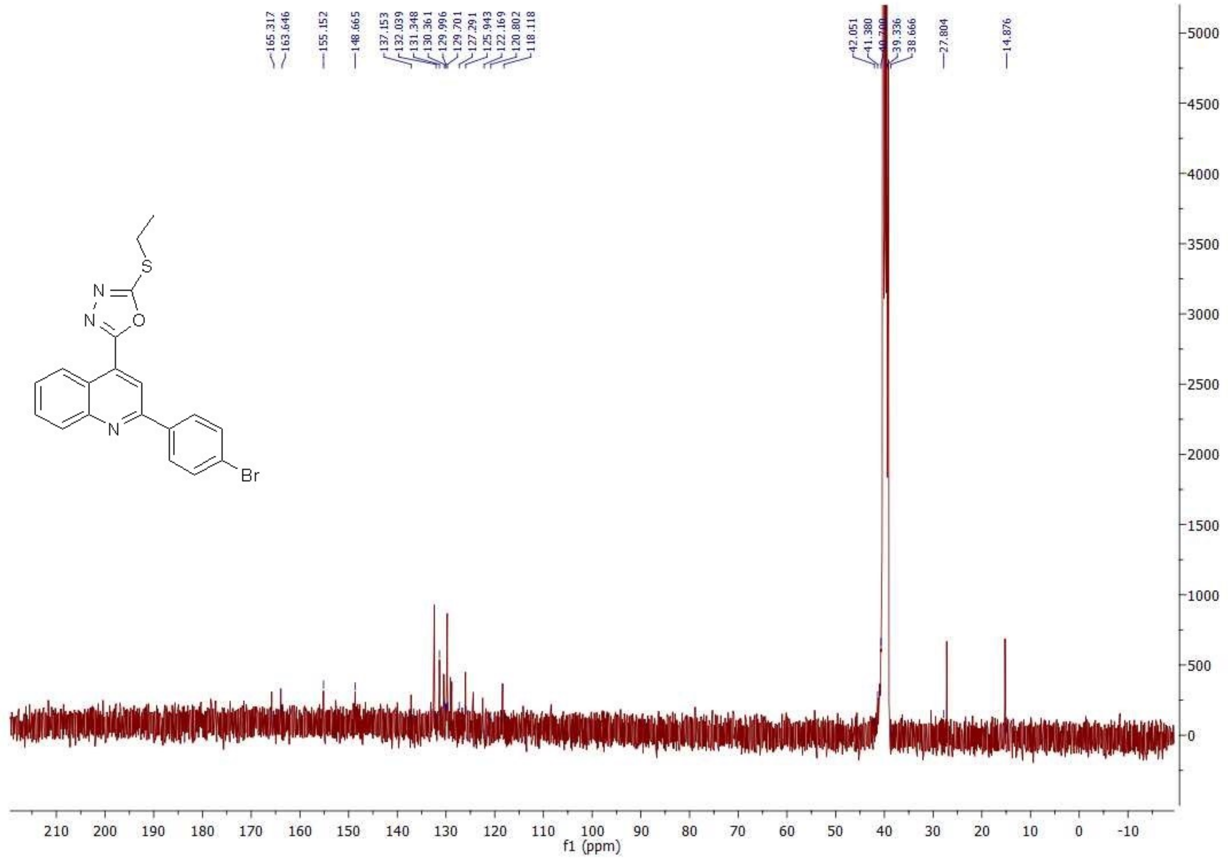

Figure S32: ^{13}CNMR spectrum of the compound **12b**.

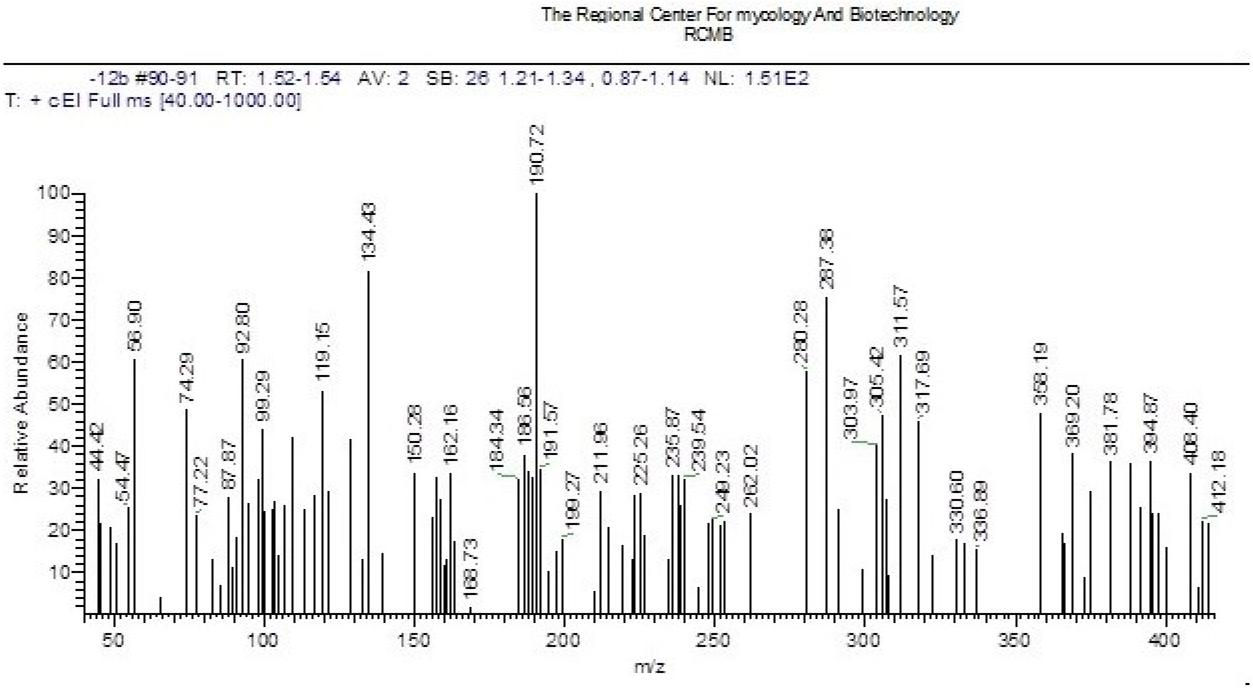

Figure S33: MS of the compound **12b**.

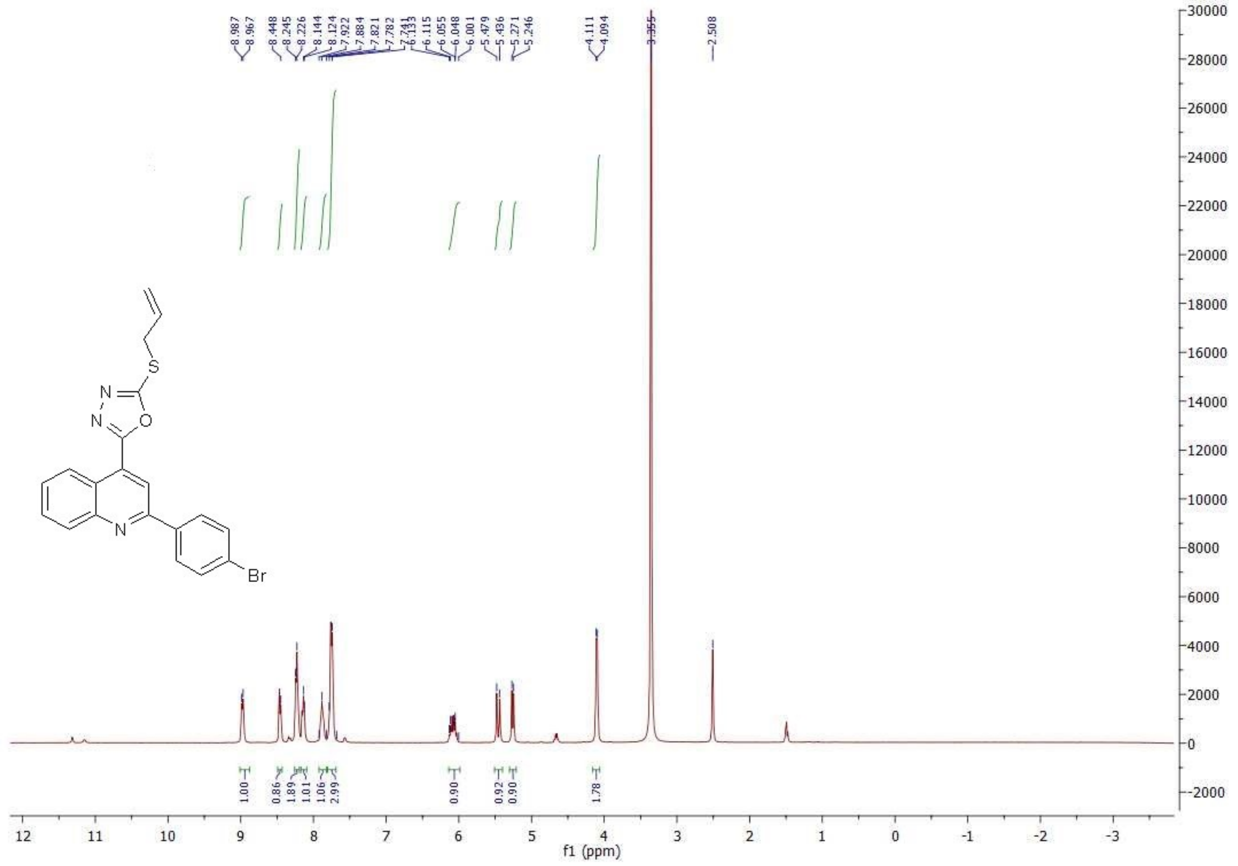

Figure S34: ¹HNMR spectrum of the compound **12c**.

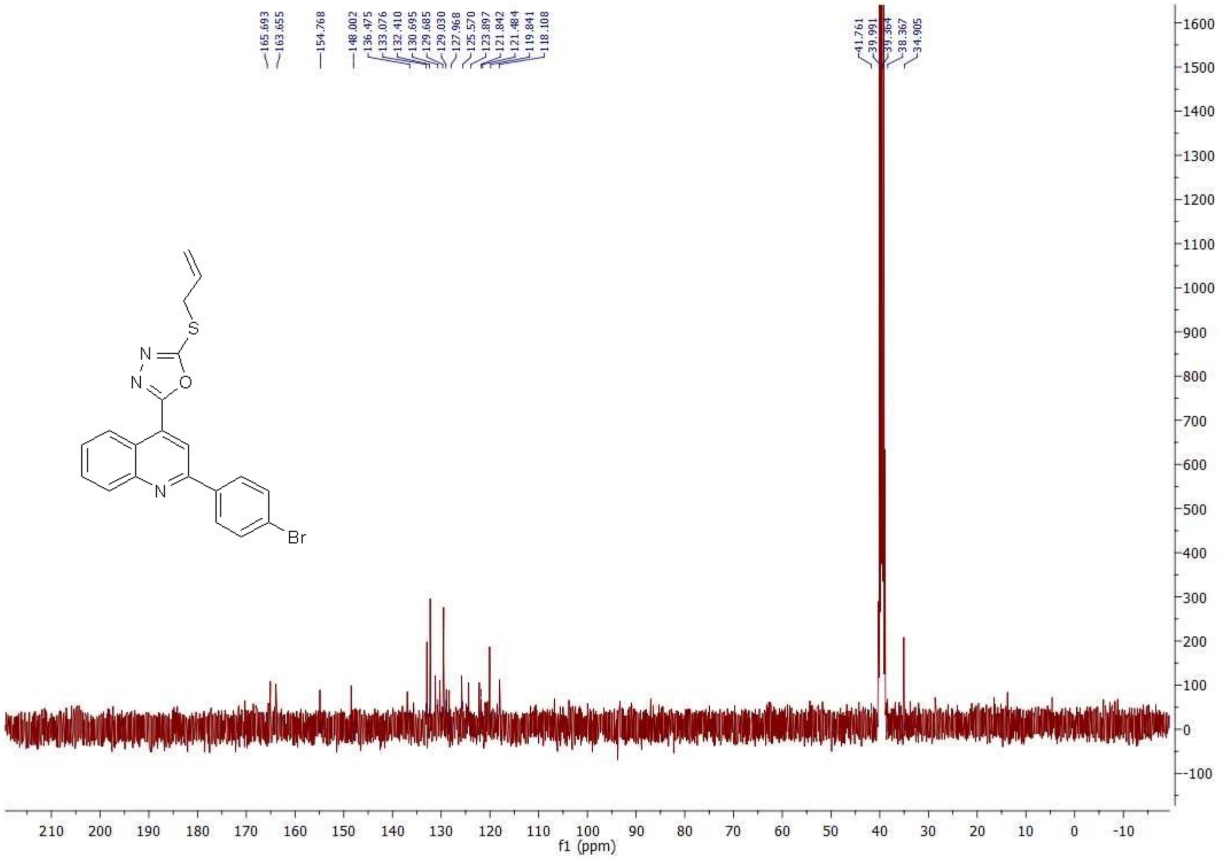

Figure S35: ^{13}CNMR spectrum of the compound **12c**.

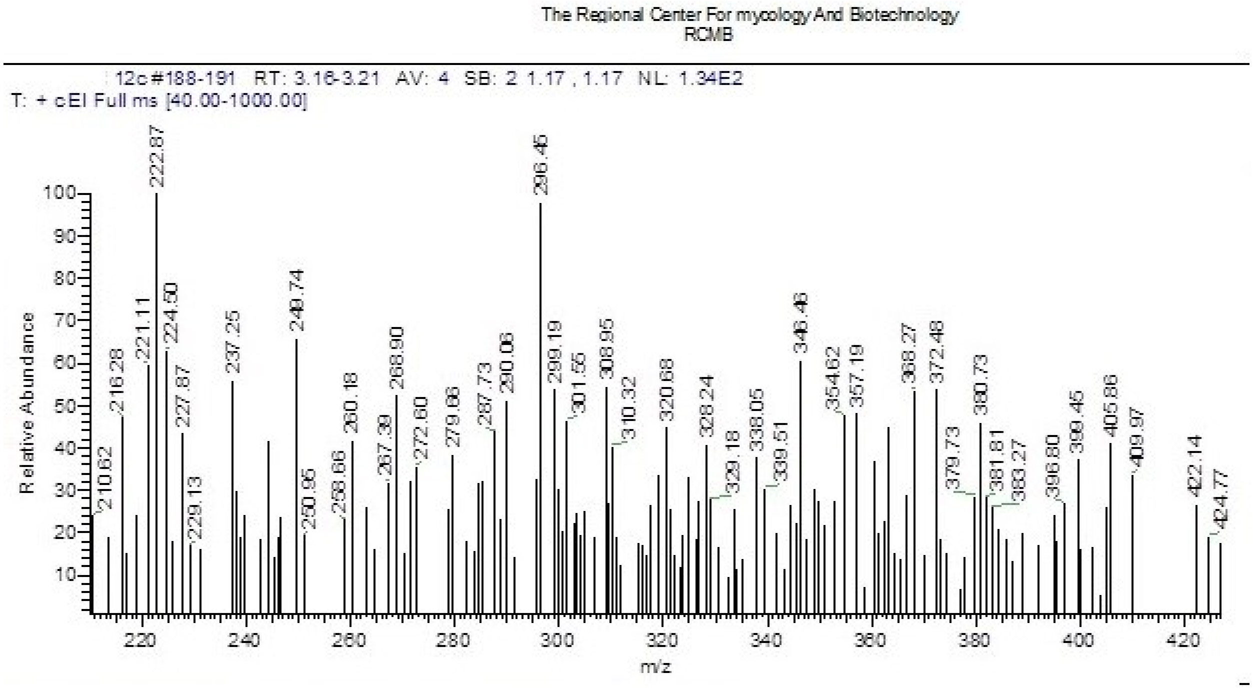

Figure S36: MS of the compound **12c**.

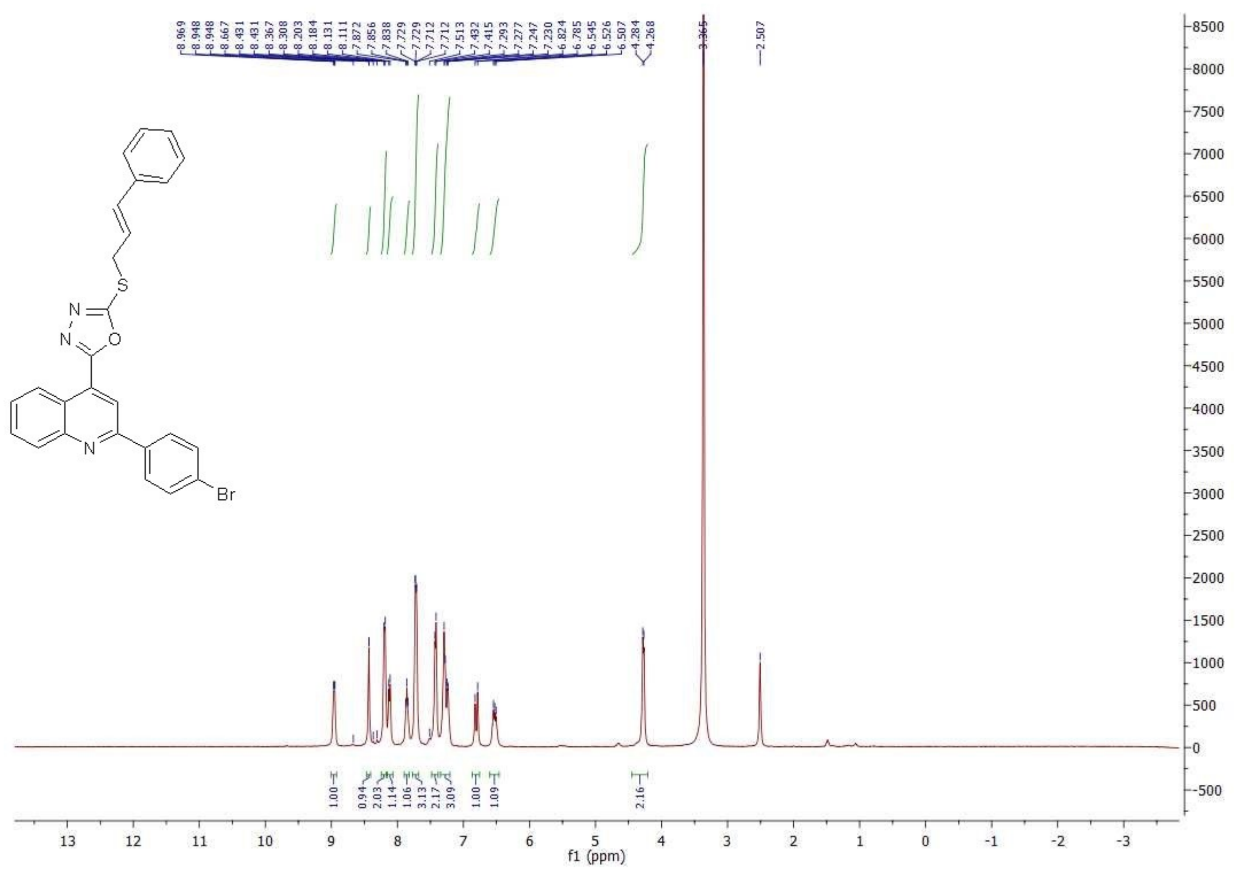

Figure S37: ^{1}HNMR spectrum of the compound **12d**.

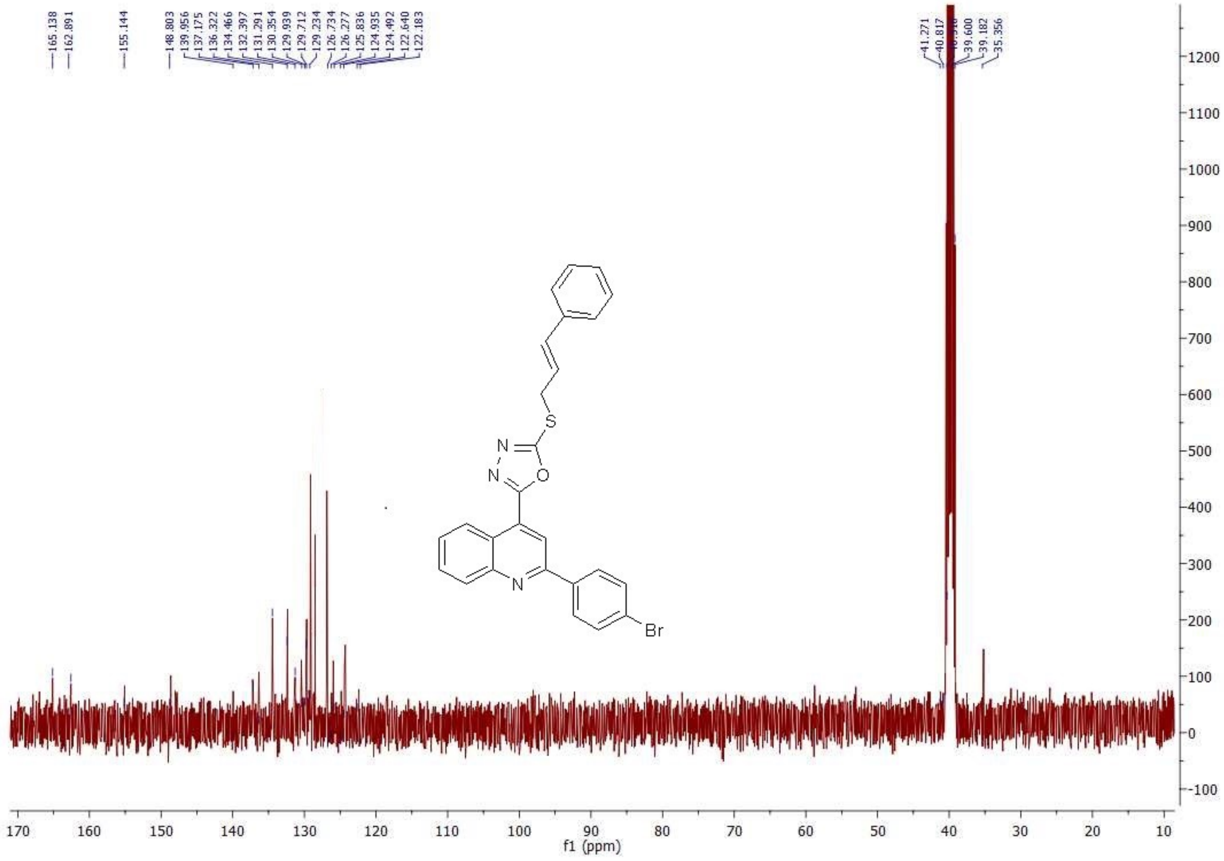

Figure S38: ^{13}CNMR spectrum of the compound **12d**.

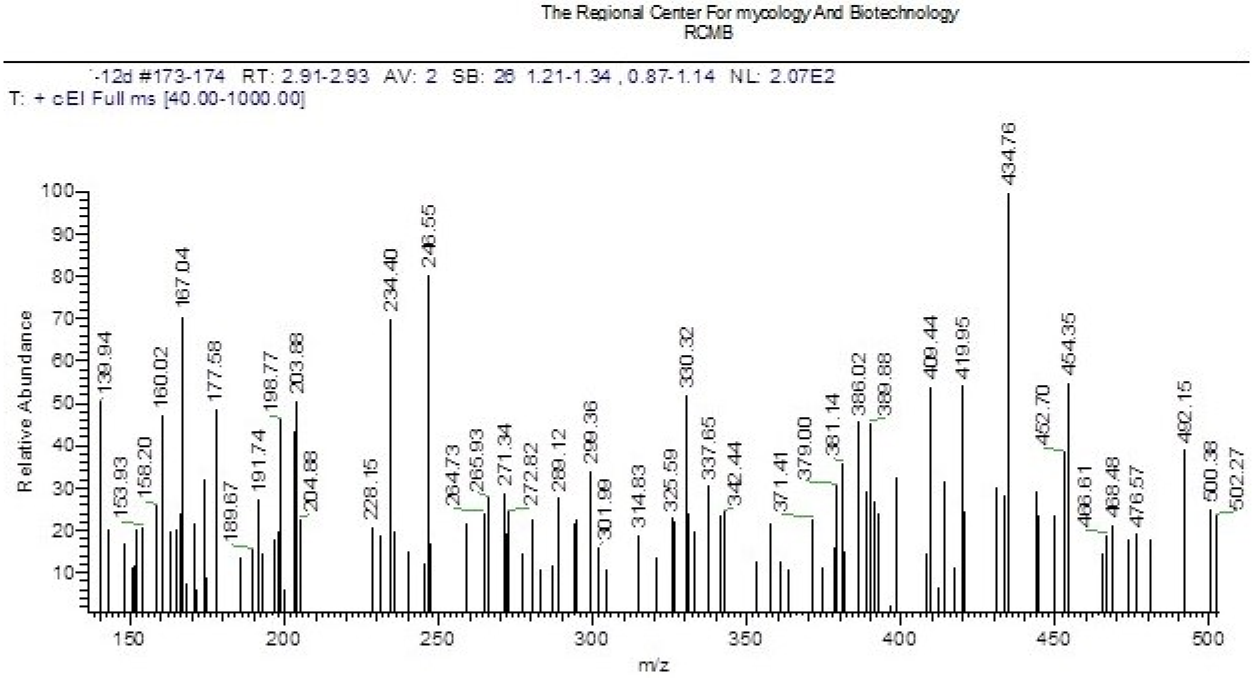

Figure S39: MS of the compound **12d**.

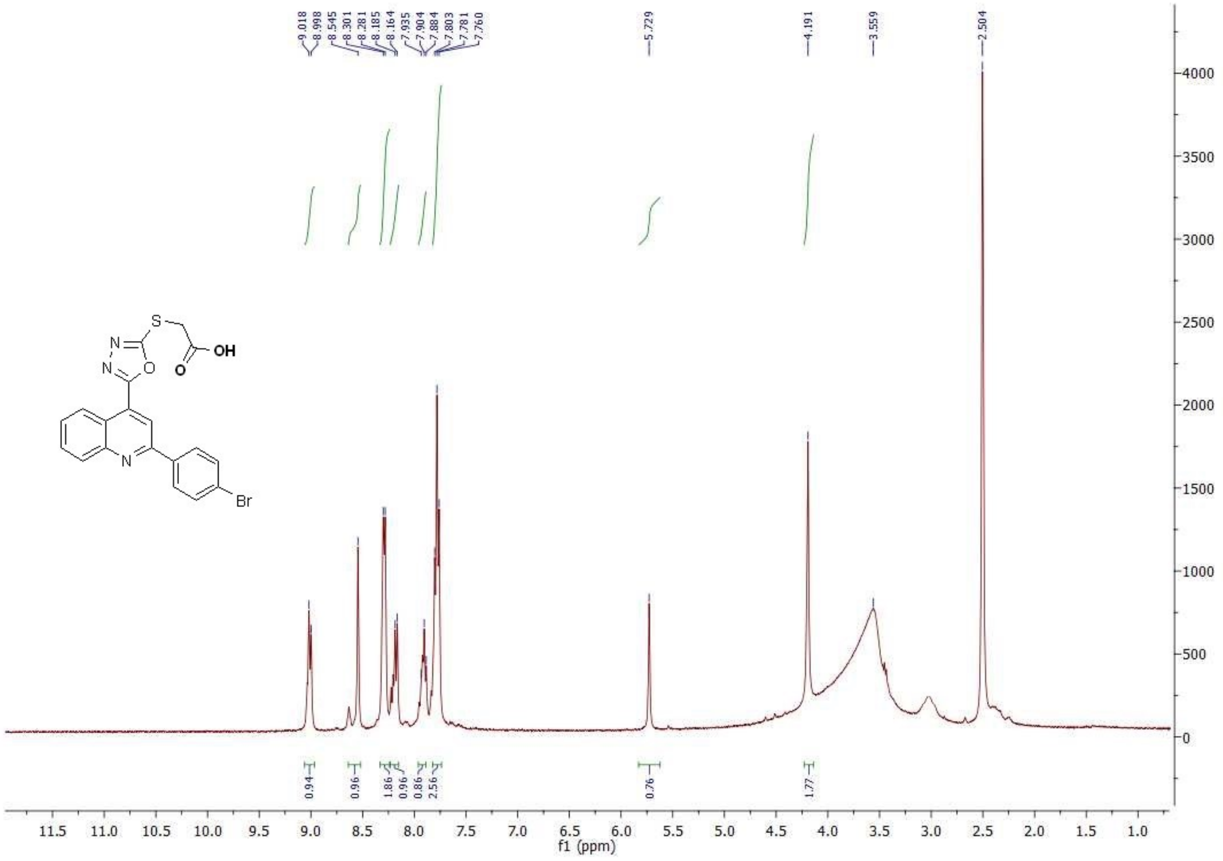

Figure S40: ^{1}HNMR spectrum of the compound **13**.

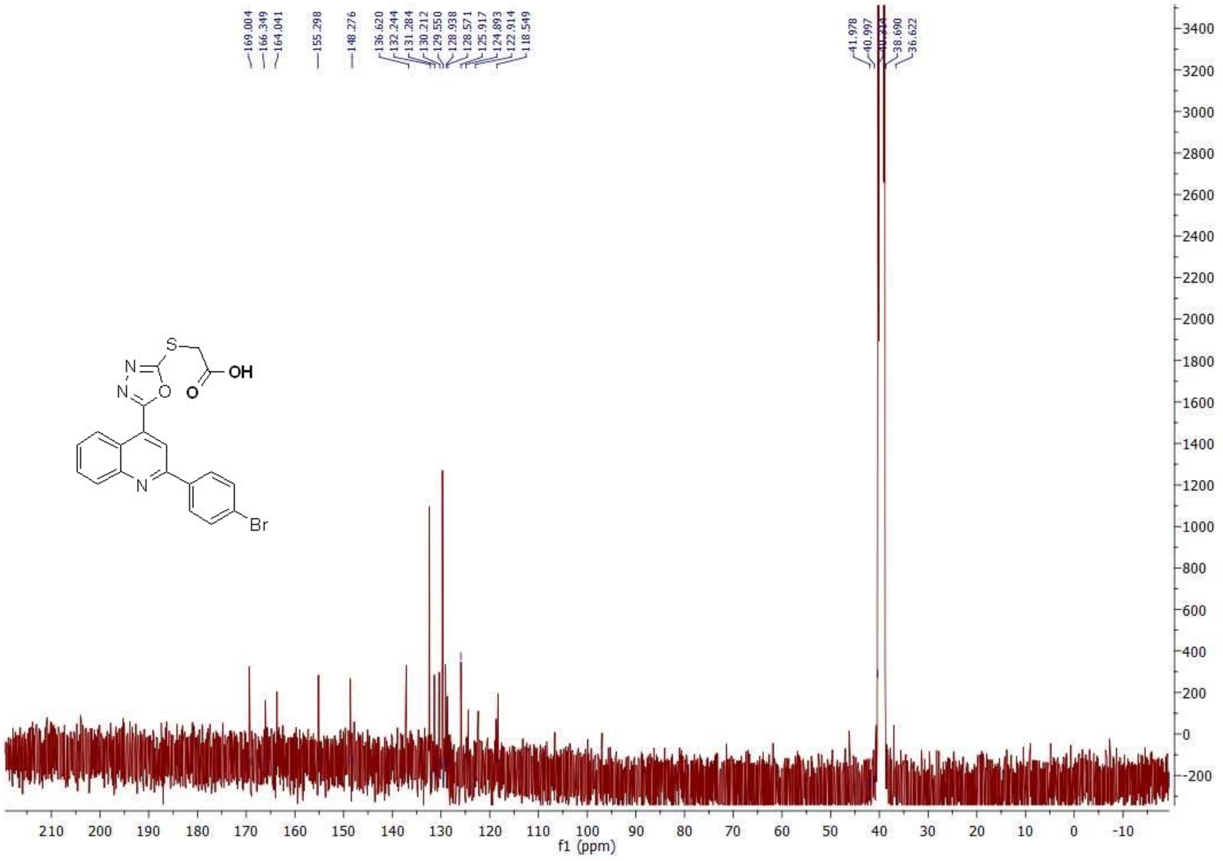

Figure S41: ^{13}CNMR spectrum of the compound **13**.

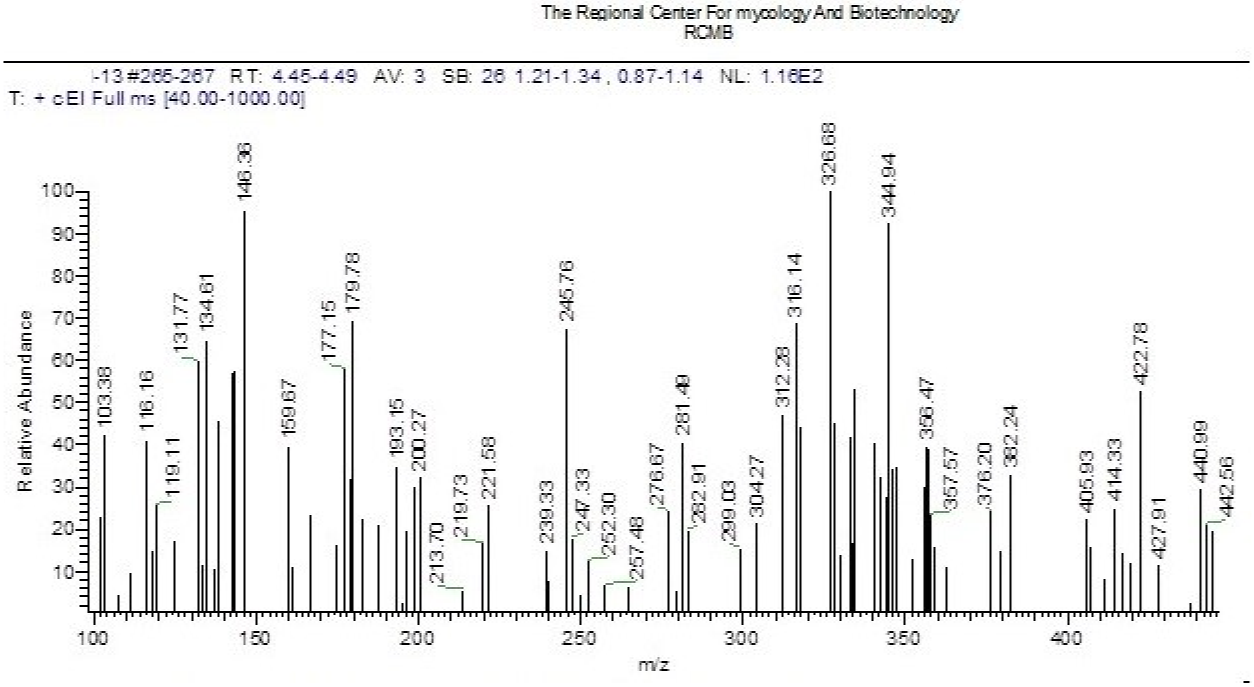

Figure S42: MS of the compound **13**.

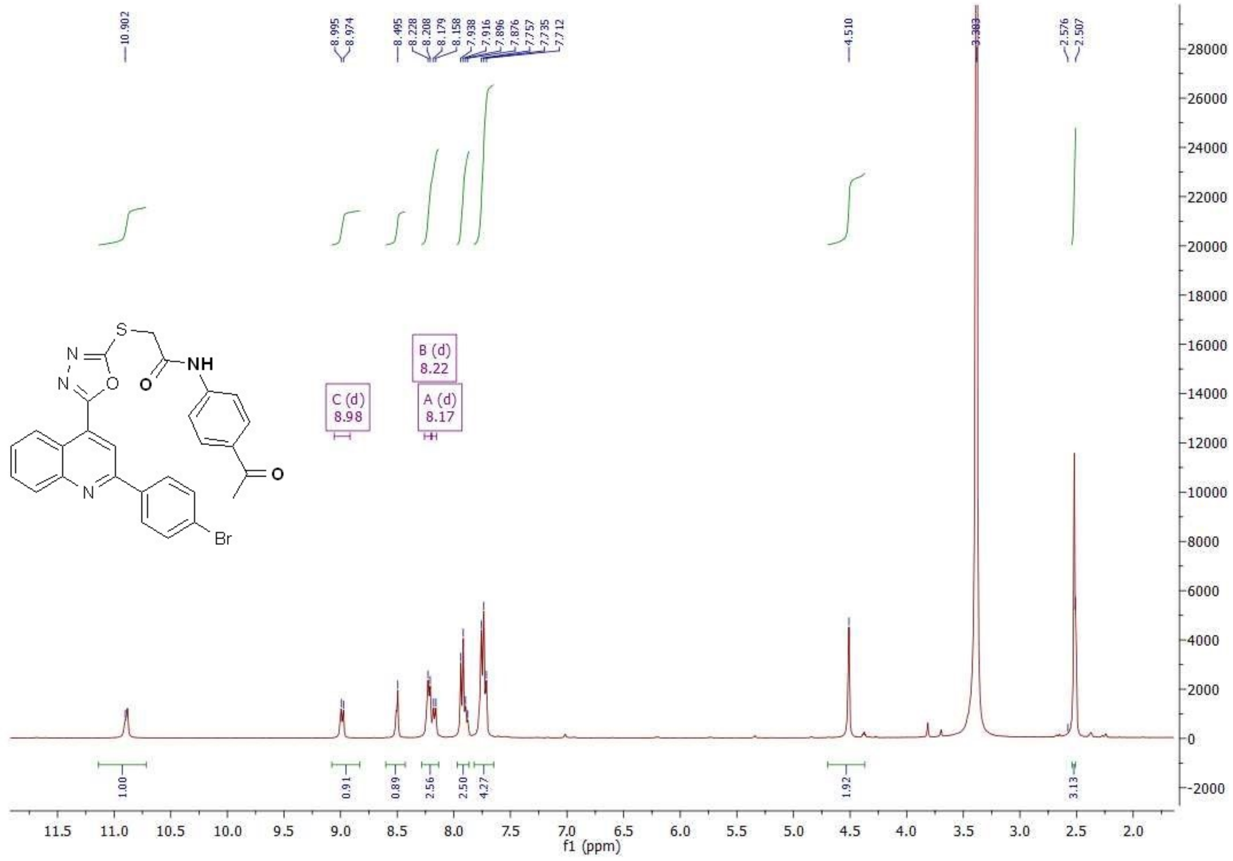

Figure S43: ^{1}HNMR spectrum of the compound **14**.

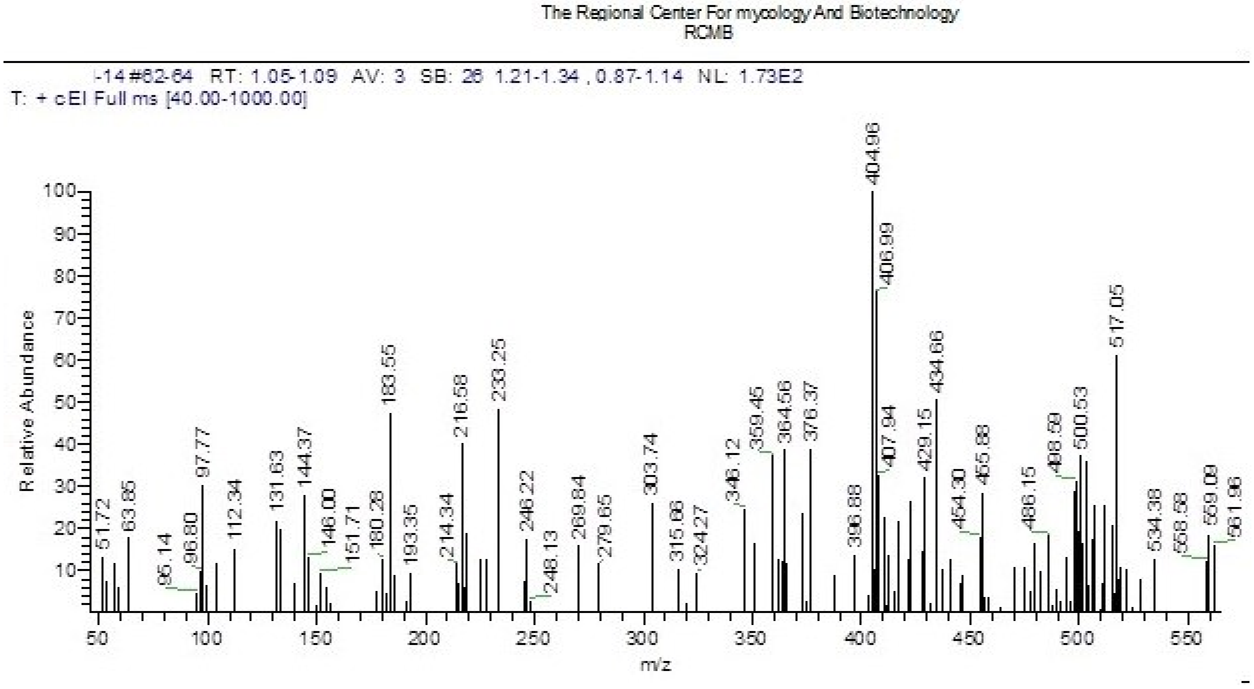

Figure S44: MS of the compound **14**.

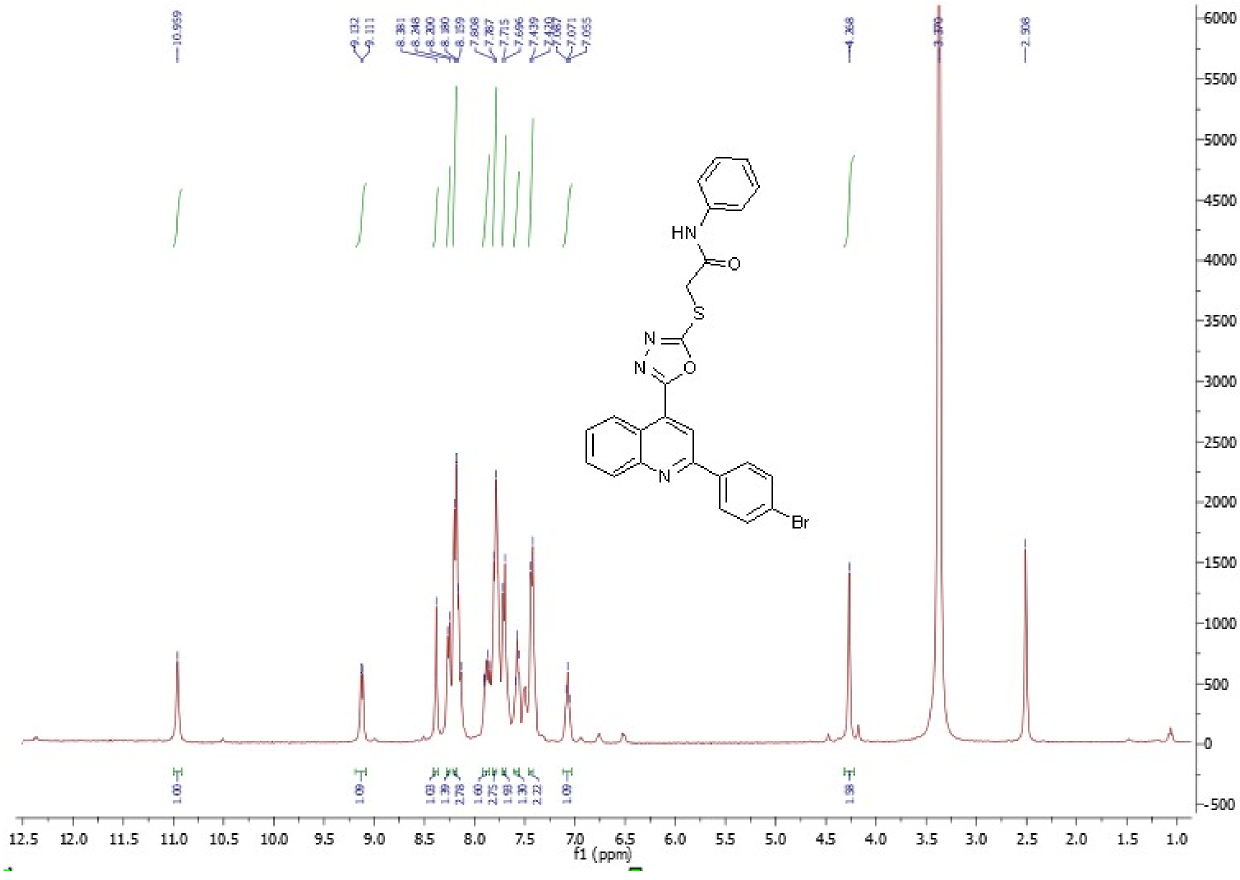

Figure S45: ^{1}HNMR spectrum of the compound **15a**.

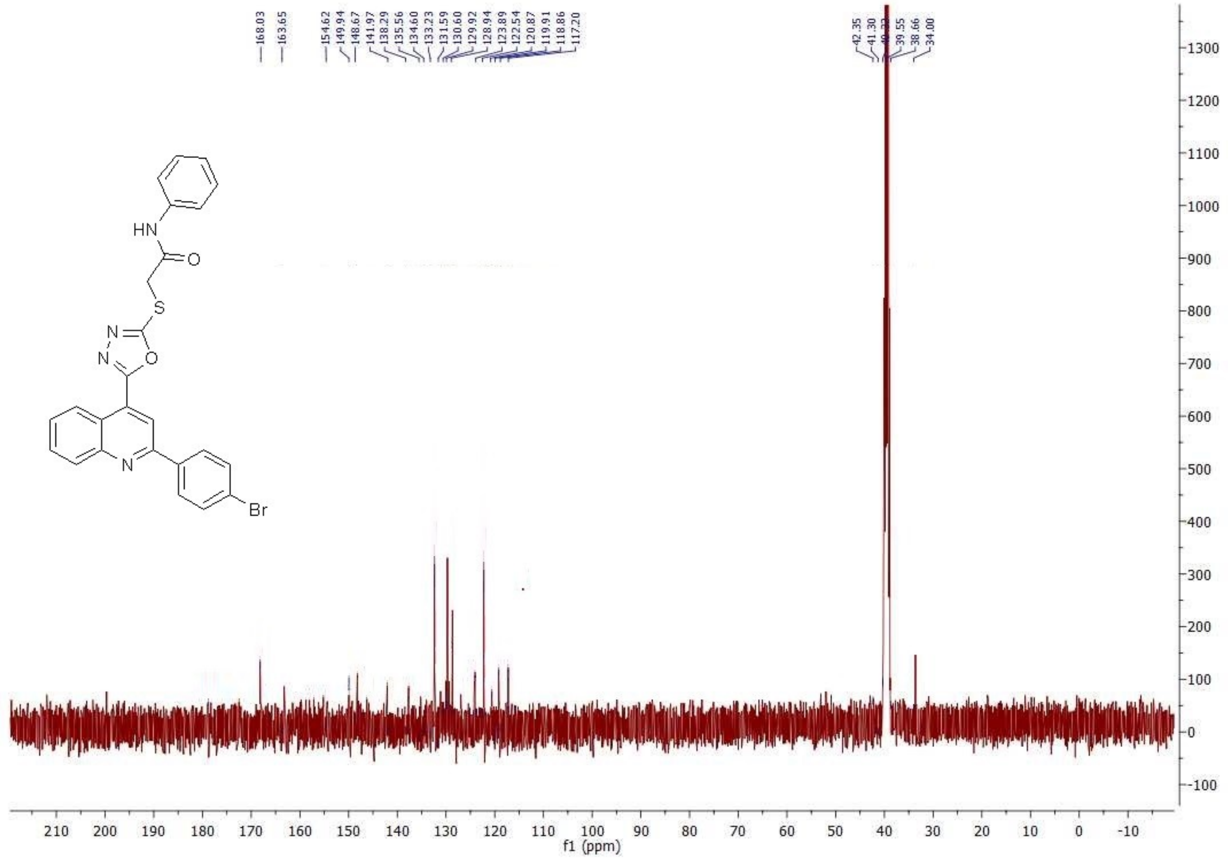

Figure S46: ^{13}CNMR spectrum of the compound **15a**.

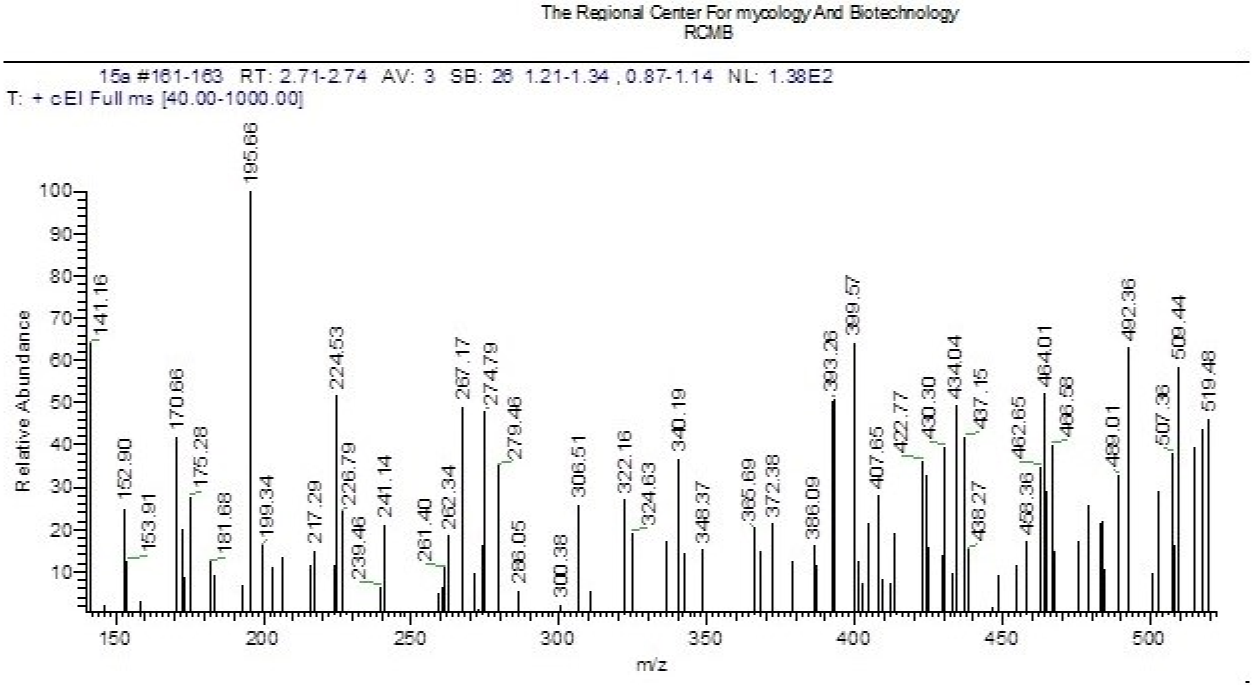

Figure S47: MS of the compound **15a**.

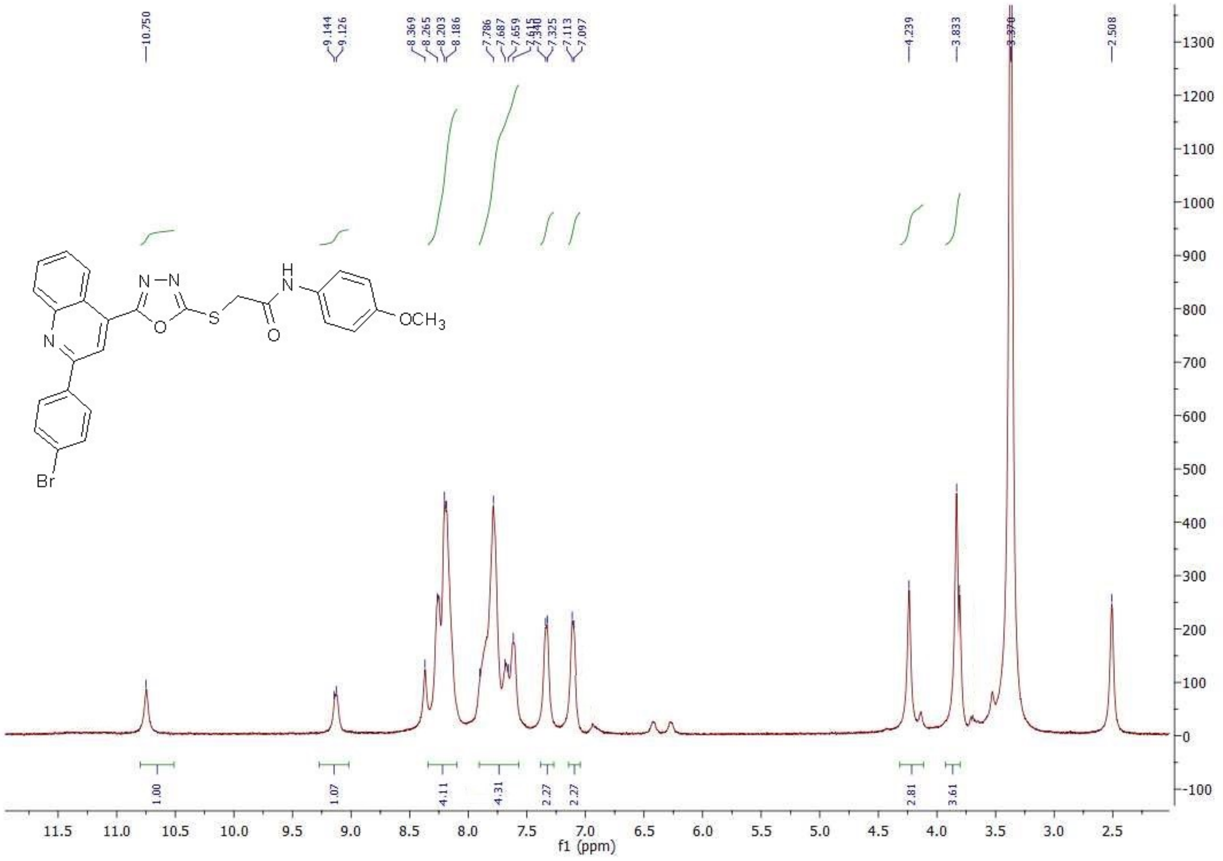

Figure S48: ^{1}HNMR spectrum of the compound **15b**.

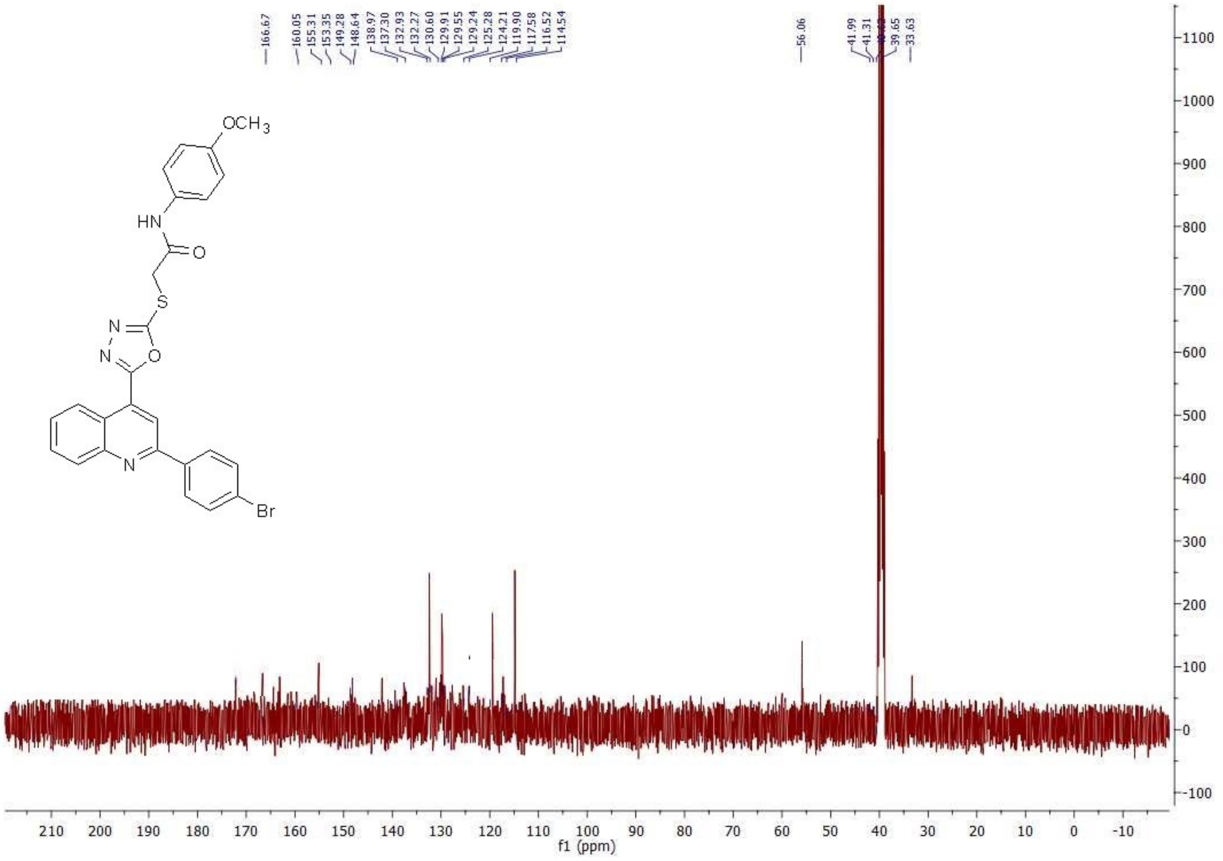

Figure S49: ^{13}CNMR spectrum of the compound **15b**.

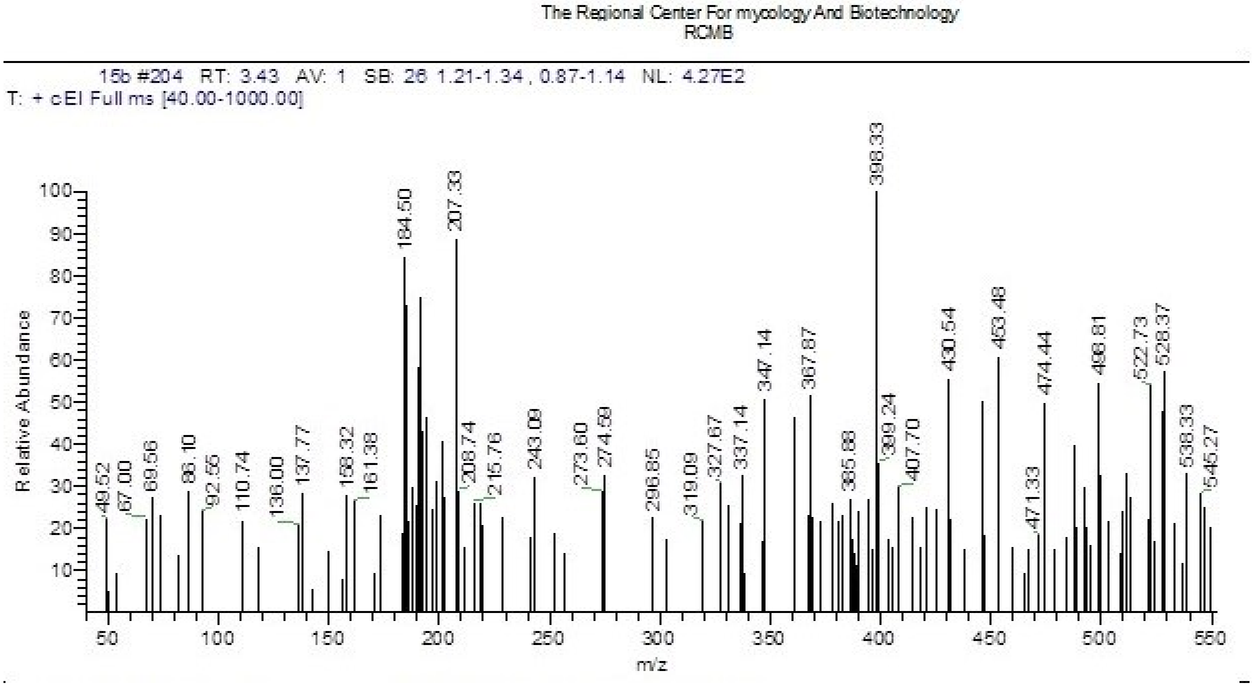

Figure S50: MS of the compound **15b**.

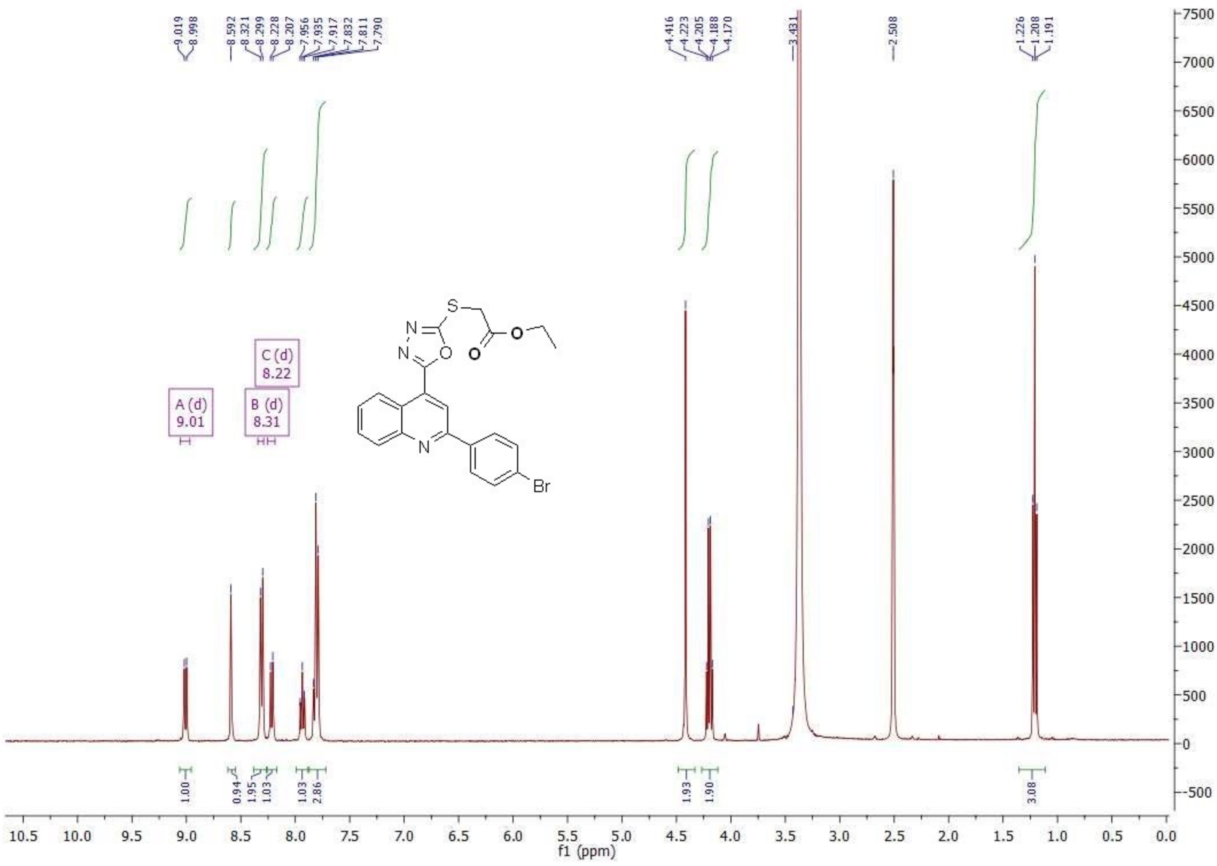

Figure S51: ^{1}HNMR spectrum of the compound **16**.

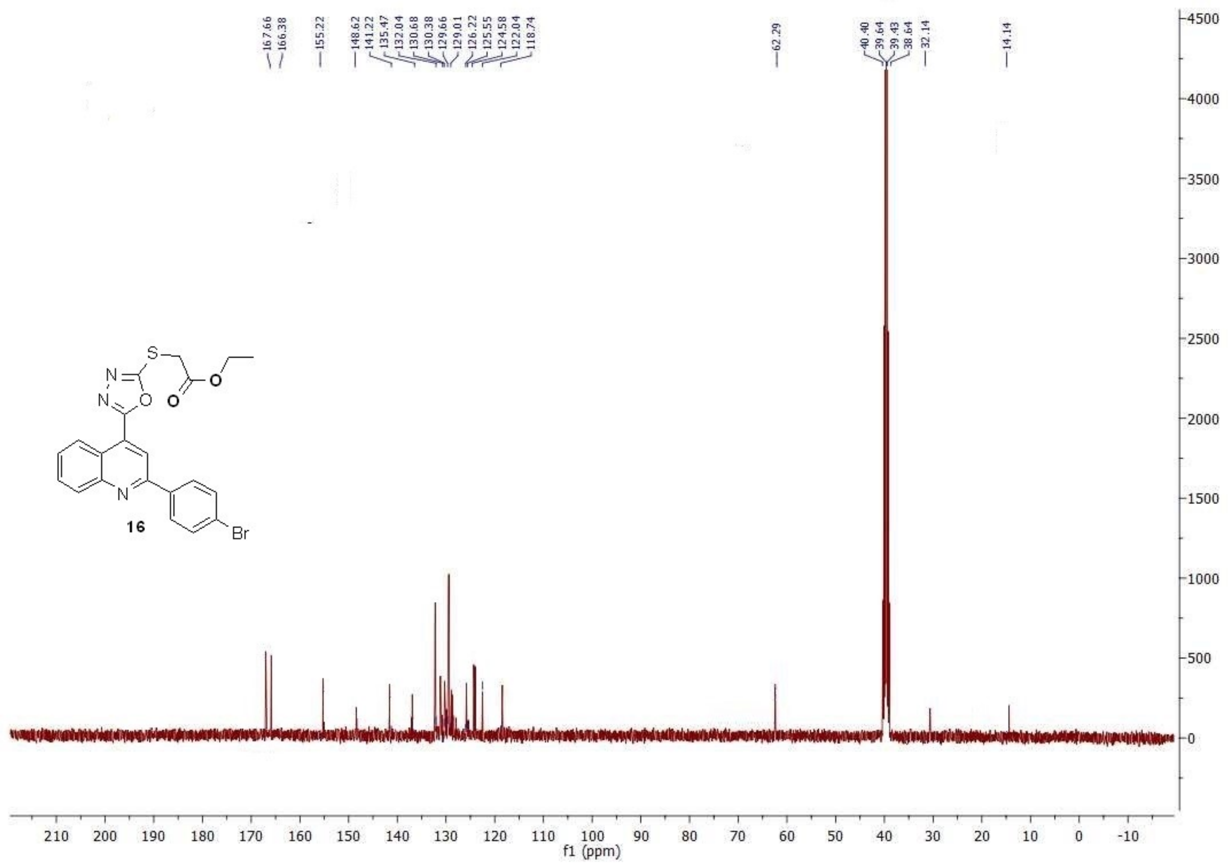

Figure S52: ^{13}CNMR spectrum of the compound **16**.

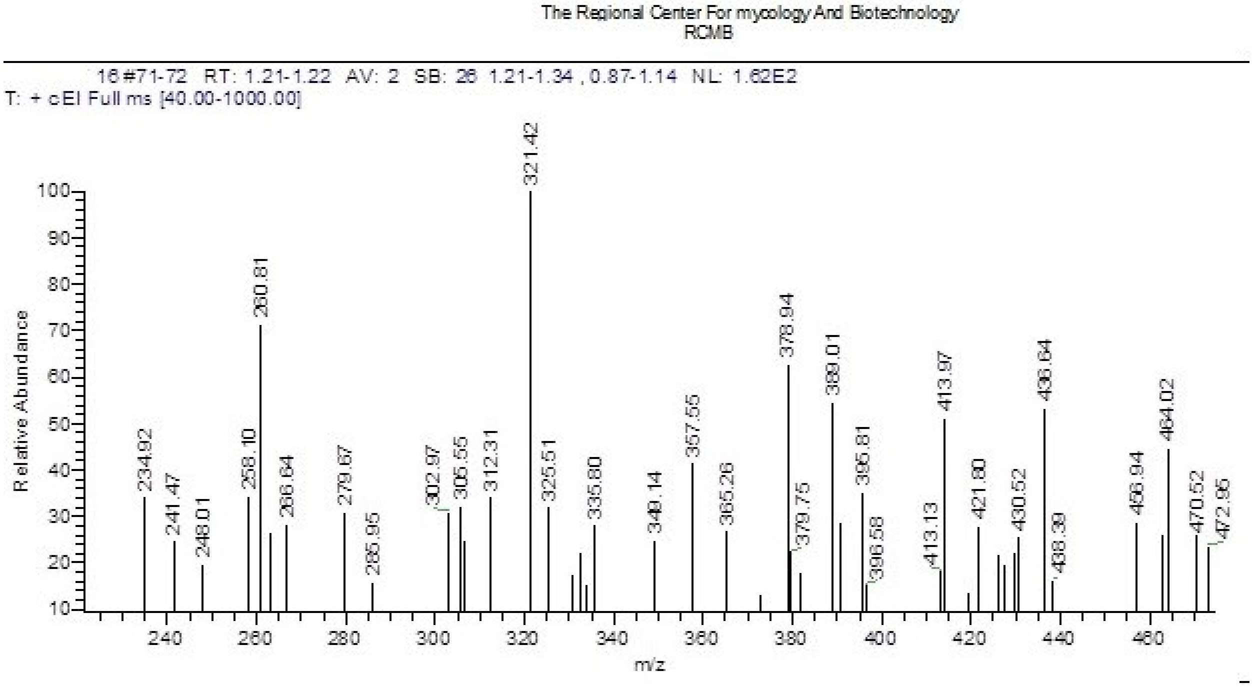

Figure S53: MS of the compound **16**.

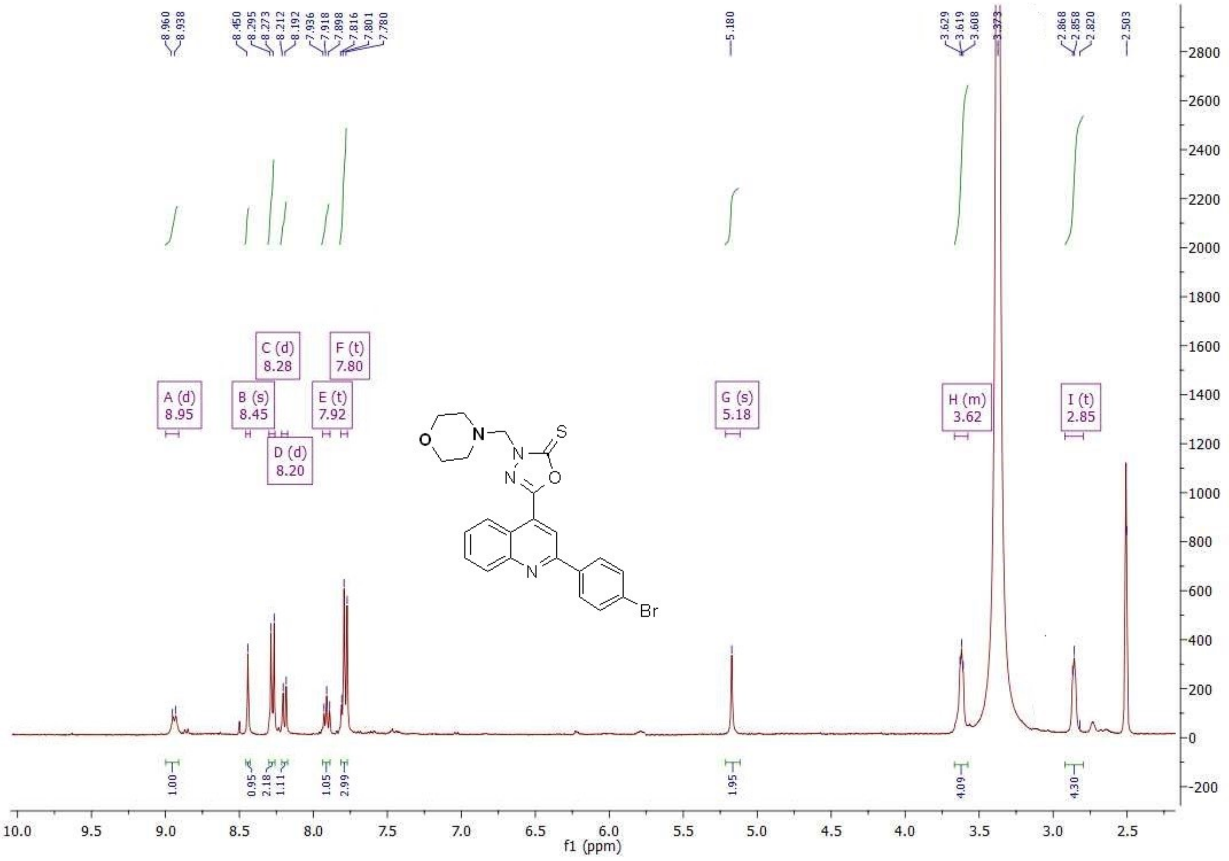

Figure S54: ^{1}HNMR spectrum of the compound **17a**.

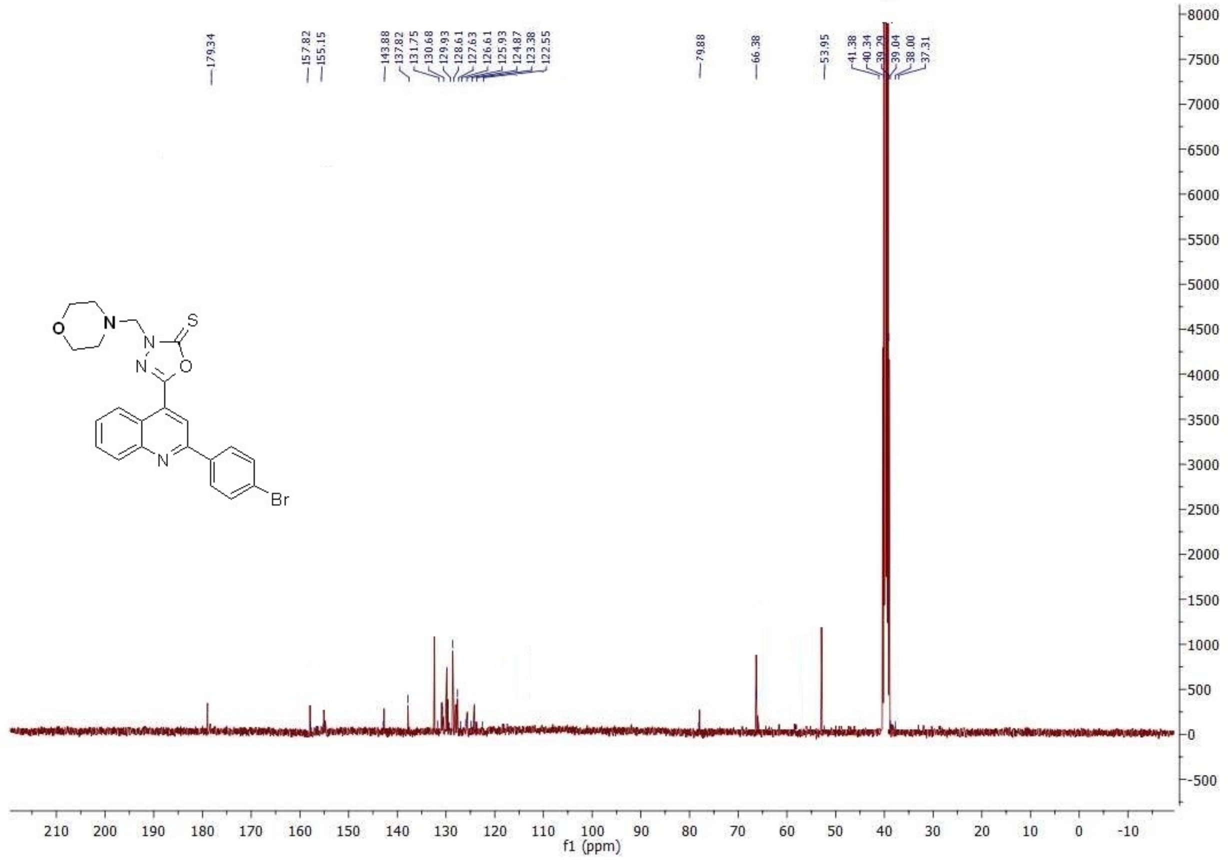

Figure S55: ^{13}CNMR spectrum of the compound **17a**.

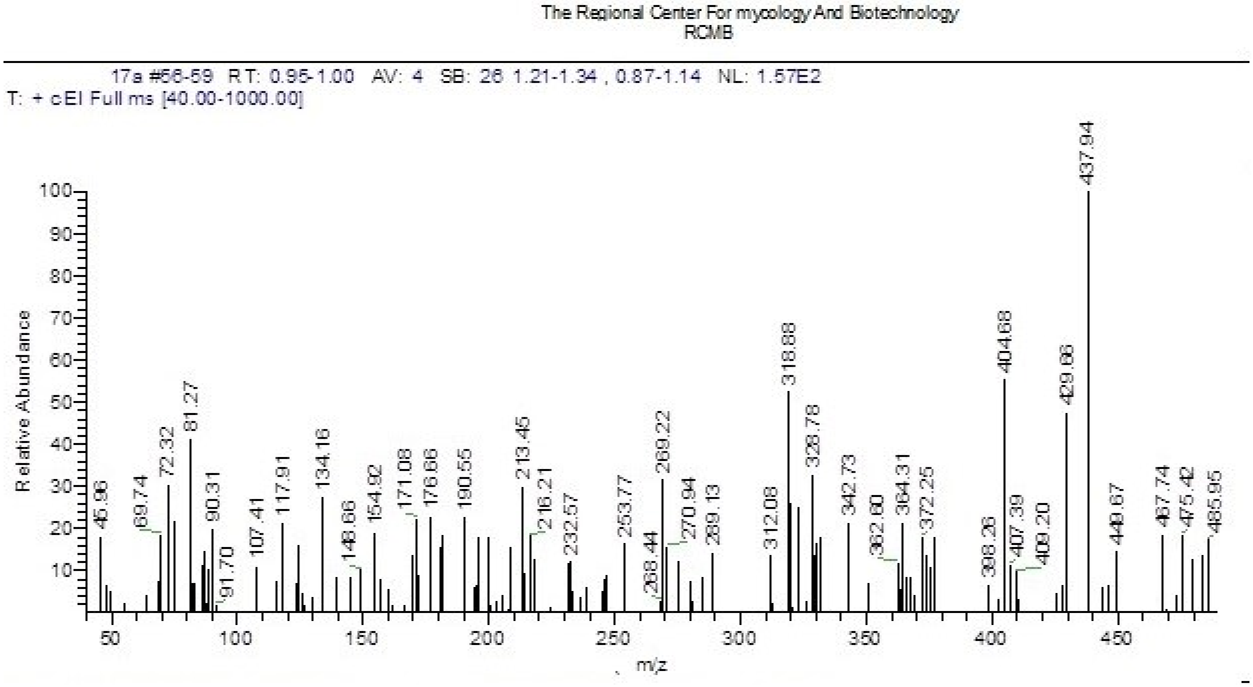

Figure S56: MS of the compound **17a**.

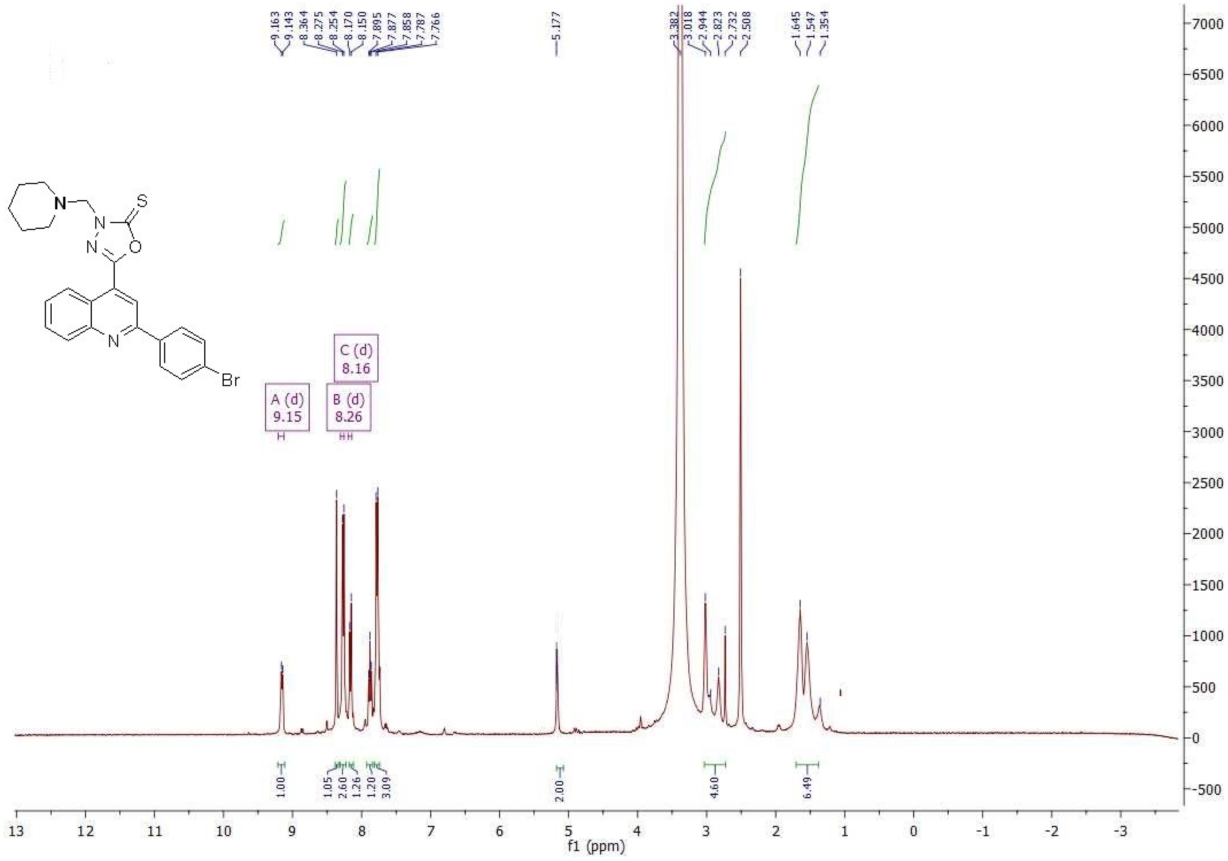

Figure S57: ^{1}HNMR spectrum of the compound **17b**.

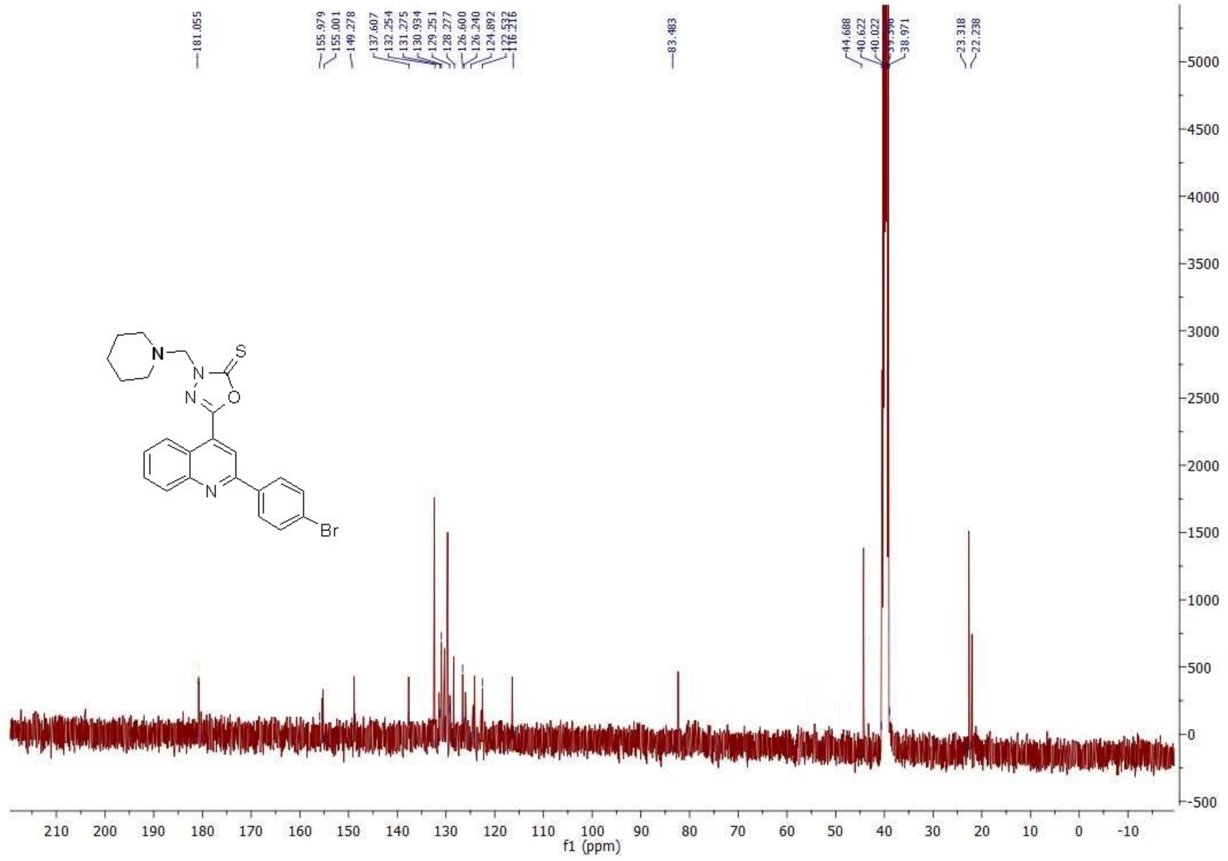

Figure S58: ^{13}CNMR spectrum of the compound **17b**.

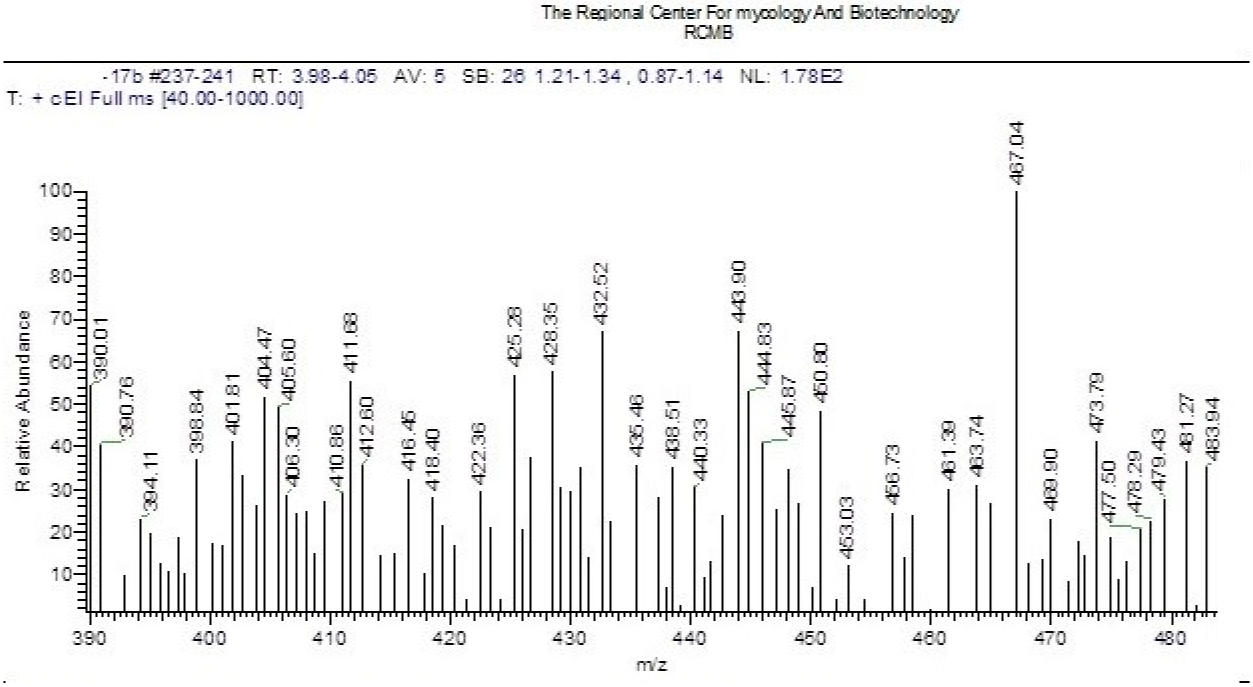

Figure S59: MS of the compound **17b**.

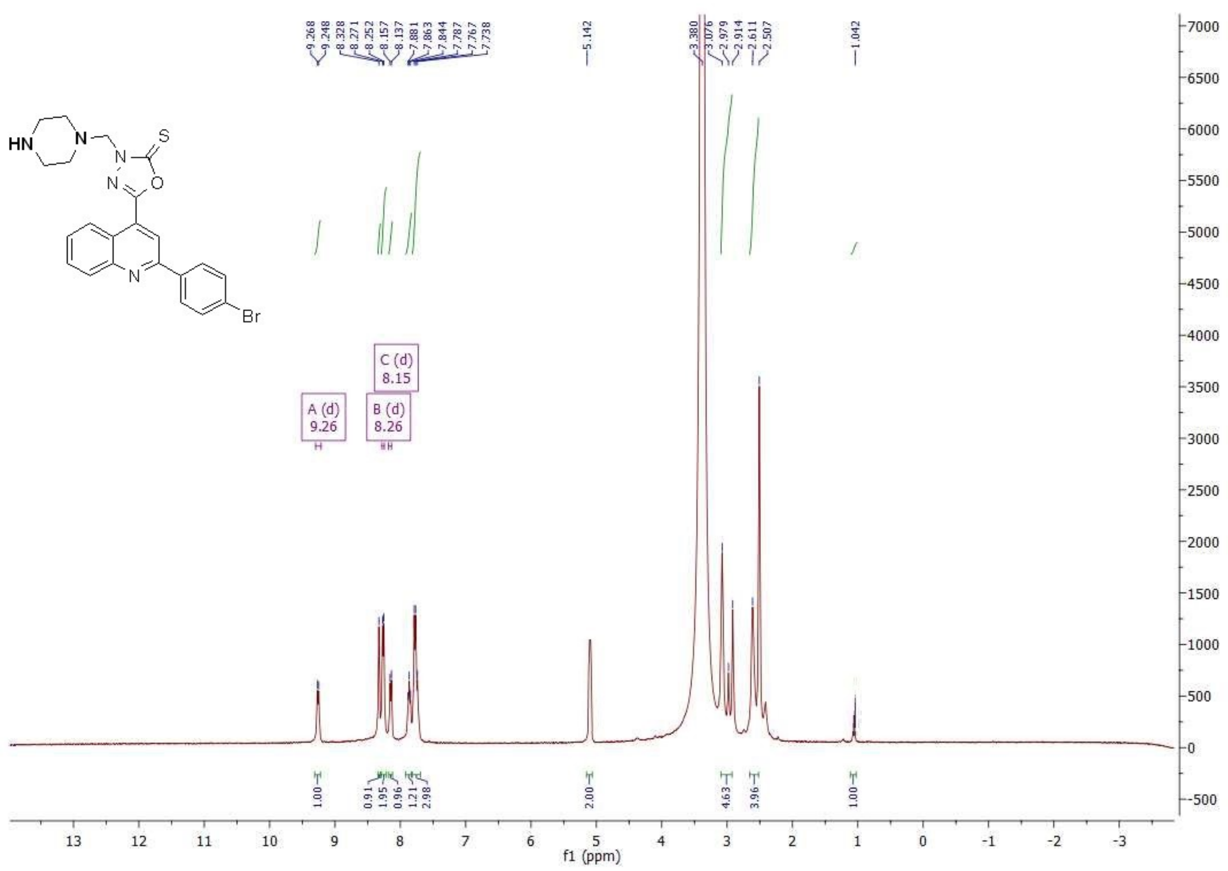

Figure S60: ^{1}HNMR spectrum of the compound **17c**.

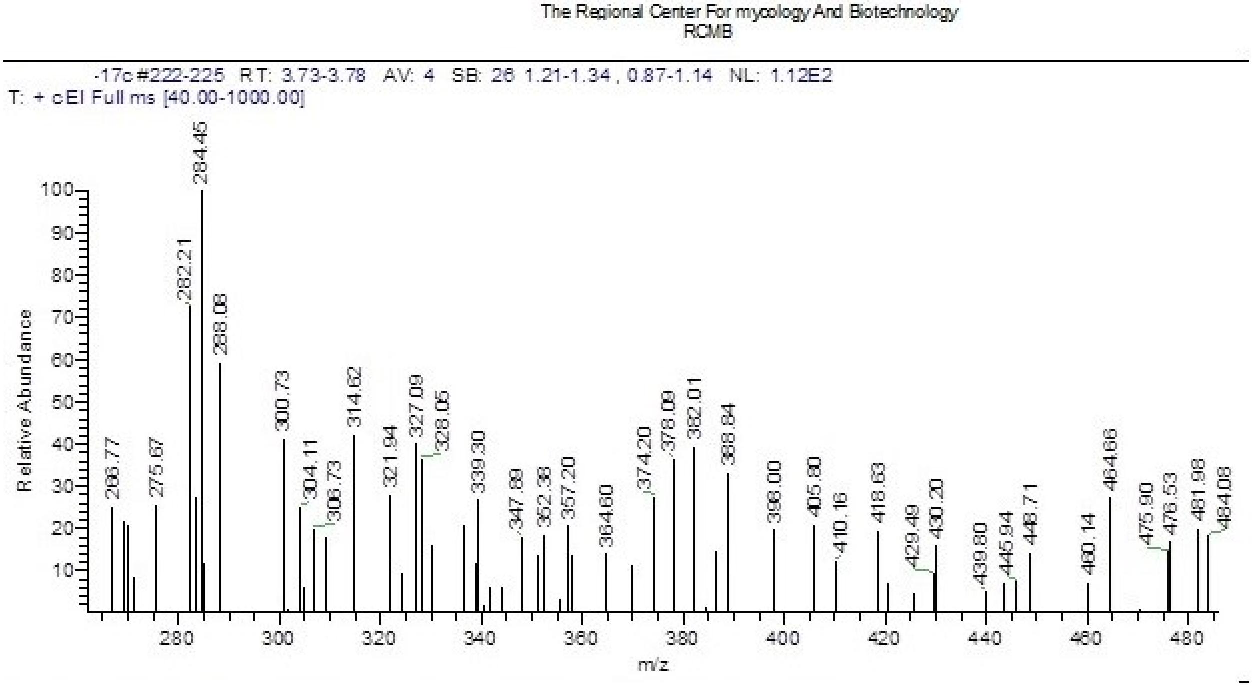

Figure S61: MS of the compound **17c**.

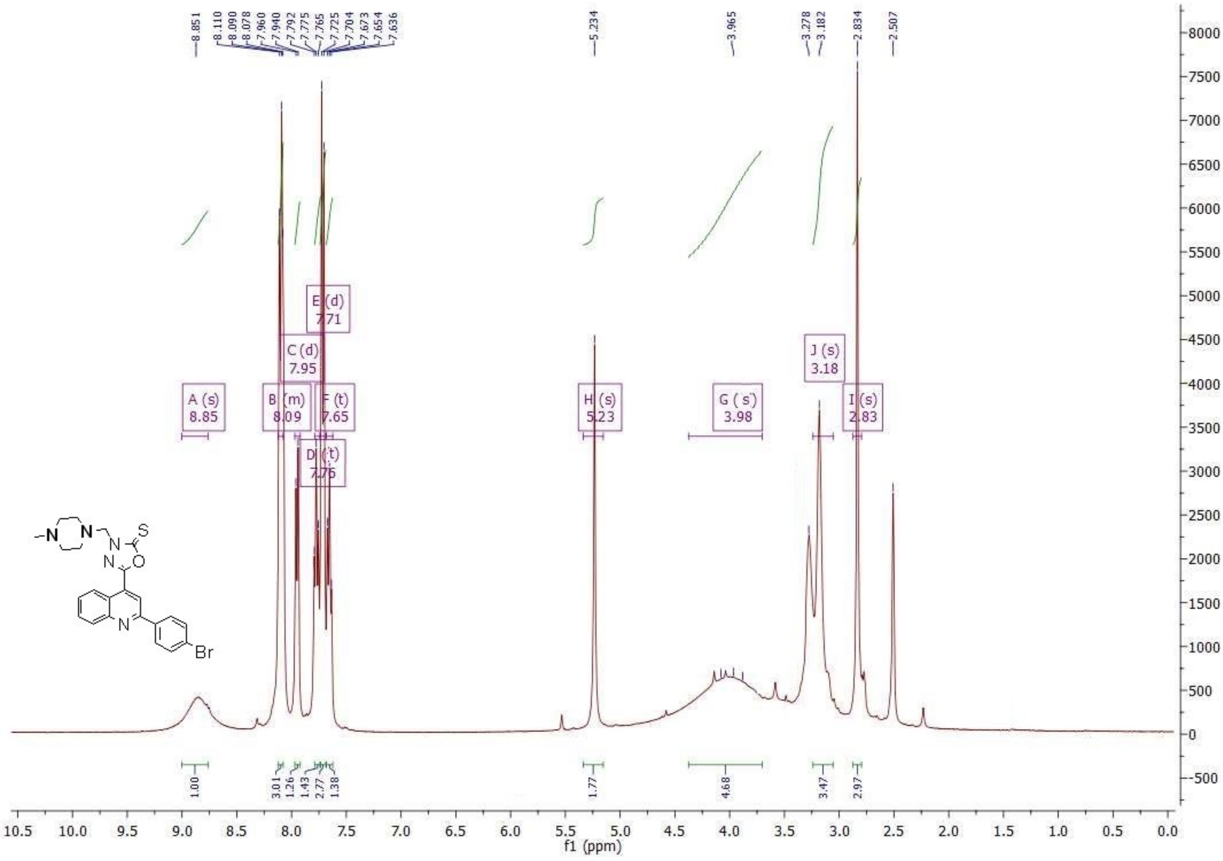

Figure S62: ^{1}HNMR spectrum of the compound **17d**.

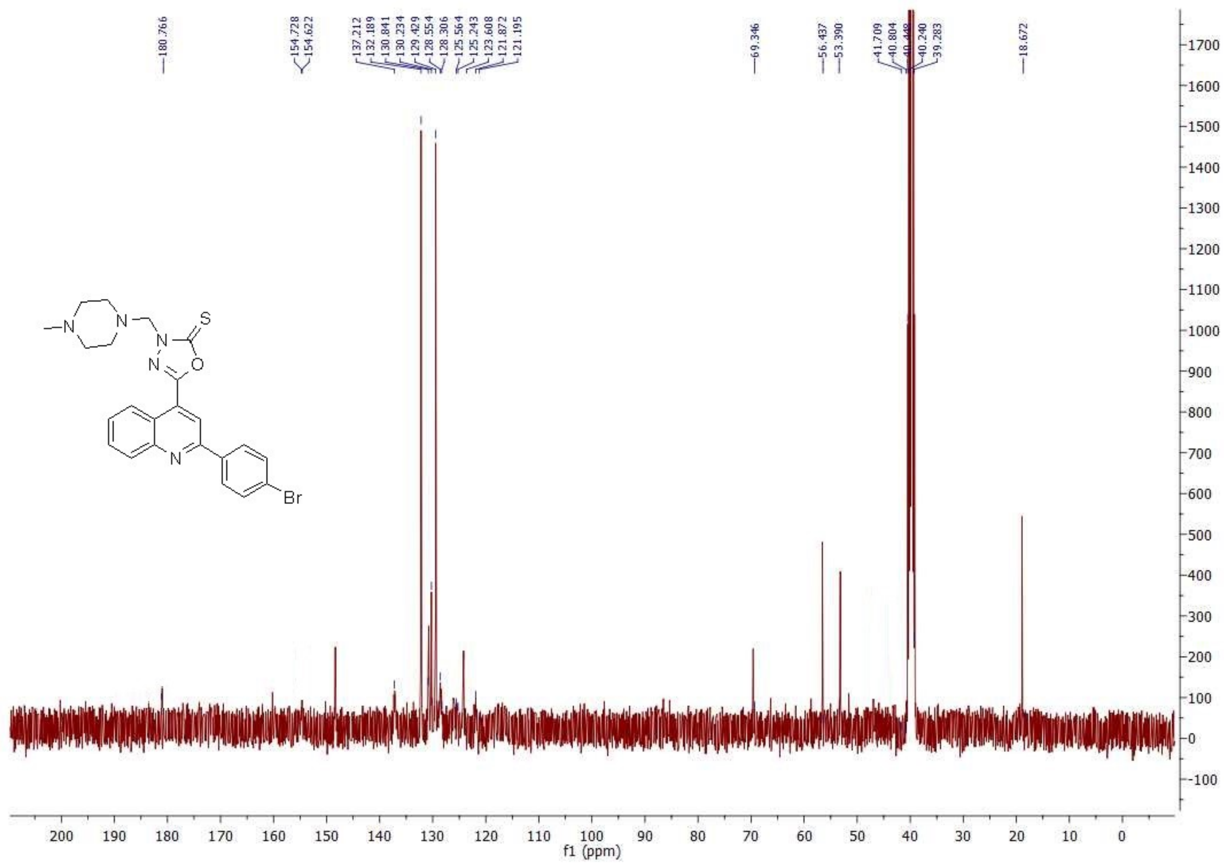

Figure S63: ^{13}CNMR spectrum of the compound **17d**.

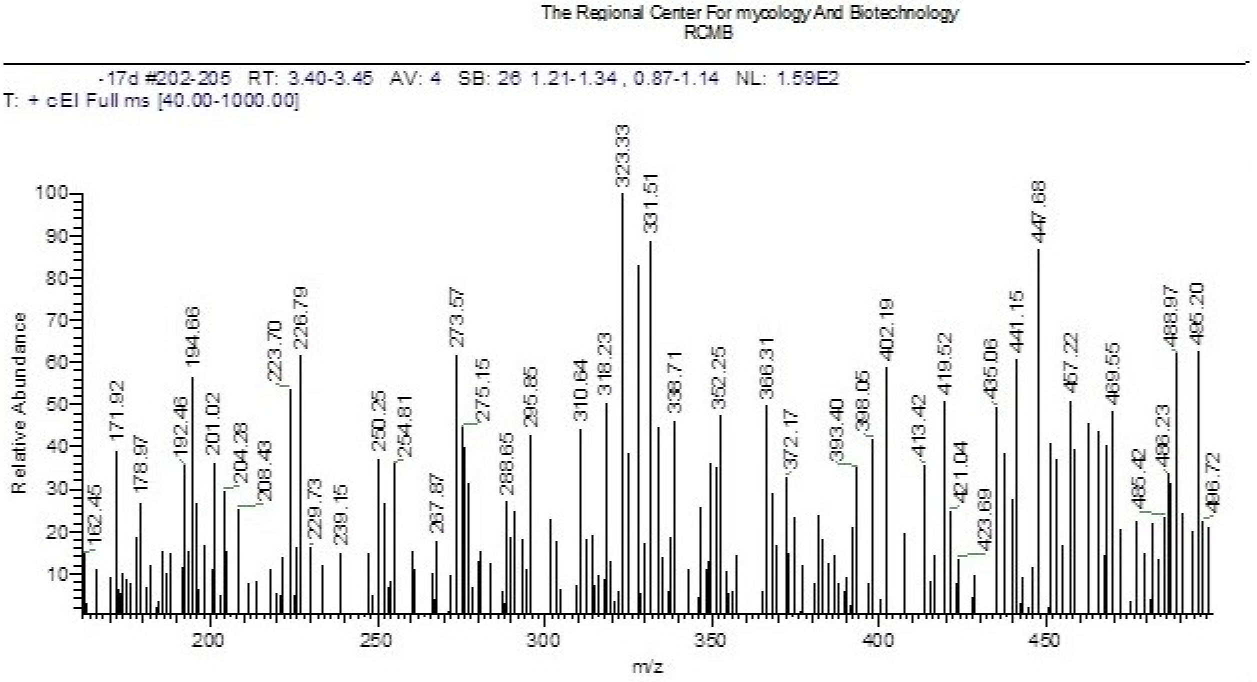

Figure S64: MS of the compound **17d**.

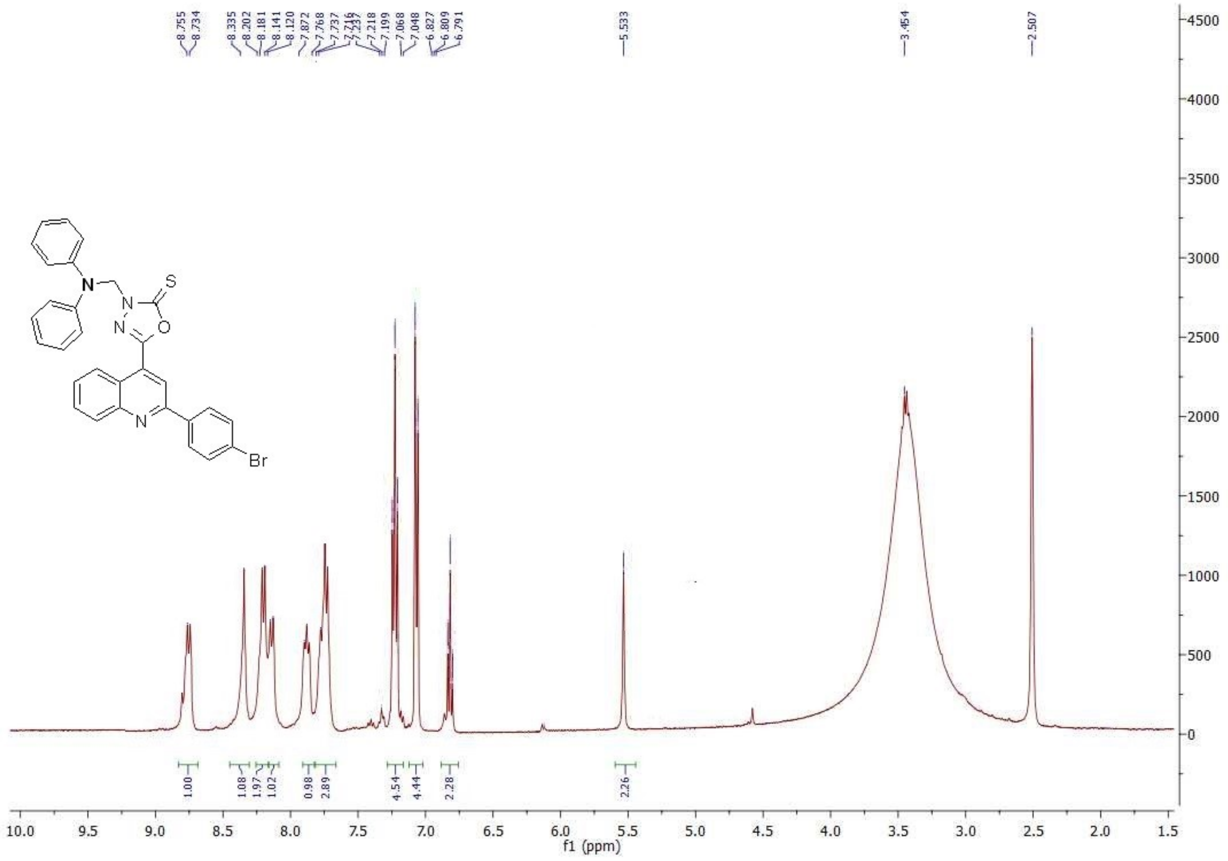

Figure S65: ^{1}HNMR spectrum of the compound **17e**.

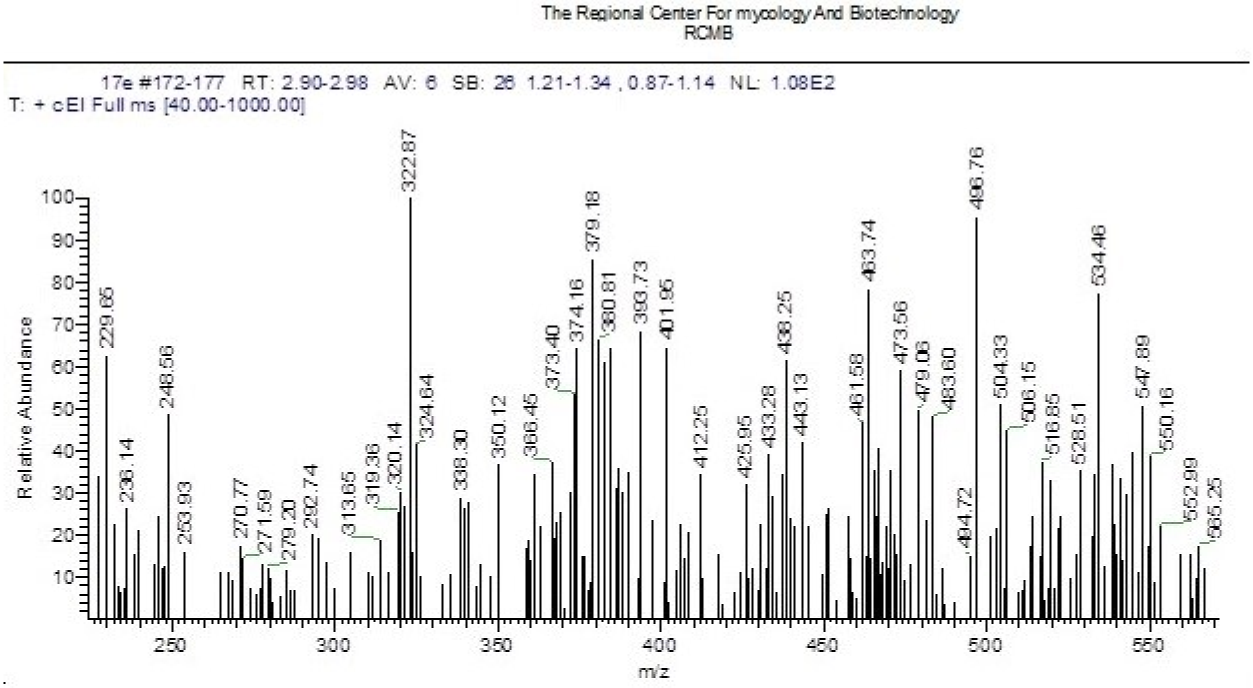

Figure S66: MS of the compound **17e**.

Appendix B

<u>Part 2</u>: Biological studies:

S4.1. Chemistry

Solvents and reagents were obtained from Aldrich and were used without further purification unless otherwise indicated. Melting points were determined by open capillary tube method using Stuart SMP10 melting point apparatus and were uncorrected. The elemental analysis was carried out by Thermo Scientific TM FLASH 2000 CHNS/O analyzer, by thermo fisher scientific at The Regional Center for Mycology and Biotechnology, Al-Azhar University, Egypt. Infrared Spectra were recorded as potassium bromide discs on Bruker FT-IR spectrophotometer, MUST university and expressed in wave number υ_{max} (cm^{-1}). ^{1}HNMR spectra were performed on Bruker 400 MHz spectrophotometer using TMS as internal standard, chemical shifts(δ) were recorded in ppm on δ scale at Ain Shams University, Egypt. ^{13}CNMR spectra were carried out using Bruker 100 MHz using TMS as internal standard, chemical shifts (δ) were recorded in ppm on δ scale at at Ain Shams University, Egypt. Mass spectra were run on Hewlett Packard 5988 spectrometer or shimadzu QP-2010 plus at The Regional Center for Mycology & Biotechnology, Al-Azhar University, Egypt. Progress of the reactions was monitored by TLC using precoated aluminum sheets silica gel (Merck 60 F $_{254}$) using chloroform:methanol (9.5:0.5) as the eluting system. and was visualized by UV lamp.

S4.2. Biological Studies

S4.2.1. Cytotoxic activity evaluation against two cancer cell lines

To measure the cytotoxic actvity of the prepared quinoline-oxadiazole derivatives **7–17e** in hepatocellular carcinoma (HepG2) and breast adenocarcinoma (MCF-7) cell lineS. Cell viability assay was assessed using MTT assay method. Cells at density of 1 x 10^4 were seeded in a 96-well plate at 37 °C for 24 h under 5% CO_2. After incubation, the cells were treated with different concentrations of the test hybrid **7–17e** and incubated for 24 h, then 20 µl of MTT solution at 5 mg/mL was applied and incubated for 4 h at 37 °C. Dimethyl sulphoxide (DMSO) in volume of 100 µl was added to each well to dissolve the purple formazan that had formed. The color intensity of the formazan product, which represents the growth condition of the cells, is quantified by using an ELISA plate reader (EXL 800, USA) at 570 nm absorbance. The experimental conditions were carried out with at least three replicates, and the experiments were repeated at least three times.

S4.2.1. Cell cycle analysis

Cell cycle analysis in HepG2 cells was investigated using fluorescent Annexin V-FITC/ PI detection kit (*BioVision* EZCell™ Cell Cycle Analysis Kit Catalog #K920) by flow cytometry assay. HepG2 cells at a density of 2 × 10^5 per well were harvested and washed twice in PBS. After that, the cells were incubated at 37 °C and 5% CO_2. The medium was incubated with the tested compounds **8c** and **12d** at the IC_{50} (µM) for 48 h, washed twice in PBS, fixed with 70% ethanol, rinsed again with PBS. Afterward, medium was stained with DNA fluorochrome PI for 15 min at 37 °C. The samples were immediately analyzed using *Facs Calibur* flow cytometer (Becton and Dickinson, Heidelberg, Germany).

S4.2.4. Apoptosis assay for compound 6h

Apoptosis in HepG2 cells was investigated using fluorescent Annexin V-FITC/ PI detection kit (*BioVision* Annexin V-FITC Apoptosis Detection Kit, Catalog #: K101) by flow cytometry assay. HepG2 cells at a density of 2 × 10^5 per well were treated with tested compounds **8c** and **12d** at the IC_{50} (µM) for 48 h, then the cells were harvested and stained with Annexin V-FITC/ PI dye for 15 min in the dark at 37 °C. The samples were immediately analyzed using *FACS Calibur* flow cytometer (Becton and Dickinson, Heidelberg, Germany).

S4.2.2. EGFR kinase Assay

Compounds **8c**, **12d** and Lapatinib were evaluated for their EGFR kinase inhibitory activity according to manufacturer's instructions using # BPS Bioscience *EGFR Kinase Assay Kit* Catalog # 40321.

BPS Bioscience

6042 Cornerstone Court W, Ste B
San Diego, CA 92121
Tel: 1.858.829.3082
Fax: 1.858.481.8694
Email: info@bpsbioscience.com

Data Sheet
EGFR Kinase Assay Kit
Catalog # 40321

DESCRIPTION: The epidermal growth factor receptor (EGFR; ErbB-1; HER1) is the cell-surface receptor for members of the epidermal growth factor family. Overexpression and/or hyperactivation of EGFR kinase is associated with several human cancers such as lung, glioblastoma, and epithelian tumors of the neck and head, leading to the development of anticancer therapeutics targeting EGFR. The *EGFR Kinase Assay Kit* is designed to measure EGFR Kinase activity for screening and profiling applications using Kinase-Glo® MAX as a detection reagent. The EGFR Kinase Assay Kit comes in a convenient 96-well format, with enough purified recombinant EGFR enzyme, EGFR substrate, ATP and kinase assay buffer for 100 enzyme reactions.

COMPONENTS:

Catalog #	Reagent	Amount	Storage	
40187	EGFR (wild type)	2 µg	-80°C	***Avoid multiple freeze/ thaw cycles!***
	5x Kinase assay buffer	1.5 ml	-20°C	
	ATP (500 µM)	100 µl	-20°C	
40217	50x PTK substrate Poly(Glu:Tyr 4:1)	100 µl	-20°C	
	96-well plate, white	1	Room Temp.	

MATERIALS OR INSTRUMENTS REQUIRED BUT NOT SUPPLIED:
Kinase-Glo MAX (Promega #V6071)
Dithiothreitol (DTT, 1 M; optional)
Microplate reader capable of reading luminescence
Adjustable micropipettor and sterile tips
30°C incubator

APPLICATIONS: Useful for studying enzyme kinetics and screening small molecular inhibitors for drug discovery and HTS applications.

STABILITY: Up to 6 months when stored as recommended.

REFERENCE:

Nakamura, J.L. *Expert Opin. Ther. Targets* **11(4)**:463-472 (2007)

S2.2.6. *In vitro* antimicrobial activity

10 mg from each compound was dissolved in 2 mL of DMSO and 100µl from each compound was tested with a final dose of 500µg for each treatment. Nutrient agar plates were heavily inoculated regularly with 0.1ml of 10^5-10^6 cells/ml in case of bacteria and yeast. Czapek-Dox agar plates seeded by 0.1ml (10^6 cells/ml) the fungal inoculum was used to evaluate the antifungal activities. Three holes were initiated in each inoculated plate. 100 microleter from

each sample were dispensed in each cup. Then plates were kept at low temperature (4°C) for 2-4 hours to allow maximum diffusion. The plates were then incubated at 37°C for 24 hours for bacteria and at 30°C for 48 hours in upright position to allow maximum growth of the organisms. The antimicrobial activity of the test agent was determined by measuring the diameter of zone of inhibition expressed in millimeter (mm). The experiment was carried out more than once and mean of reading was recorded.

S4.2.7. MIC and MBC methodology

S4.2.7.1. Preparation of bacterial culture

Bacterial cultures were prepared under sterile conditions by incubating 100 ml bottle with each test microbe, capped and incubated at 35 °C for 24 h. Clean bacterial cells were prepared by centrifuging the growth culture, under sterile condition, in cooling centrifuge at 400 rpm for 15 min. The bacterial cells were re-suspended using 20 mL of sterile normal saline and centrifuged again at 4000 rpm for 5 min. This step was repeated until the supernatant was clear. The pellet was then suspended in 20 mL of sterile normal saline. The optical density of the bacterial suspension was recorded at 500 nm, and serial dilutions were carried out with appropriate aseptic techniques until the optical density was in the range of 0.5–1.0. The actual number of colony-forming units was carried out to obtain a concentration of $5x10^6$ cfu/mL.

S4.2.7.2. Preparation of resazurin solution

The resazurin solution was prepared by dissolving a 675 mg in 100 mL of sterile distilled water and shake well with vortex mixer and sterilized by filtration through membrane filter (pore size of (0.22- 0.45 μm).

S4.2.7.3. Preparation of the plates

Microplates, 96 well were prepared and labelled under aseptic conditions. A volume of 500 μL of test material in DMSO (a stock concentration of 5mg/mL for purified compounds) was pipetted into the first row of the plate. To all other wells 50 μL of broth medium was added. Serial dilutions were performed. To each well 10μL of resazurin indicator solution was added, 10μL of bacterial suspension (5 $x10^6$cfu/mL) was added to each well. Each plate was wrapped loosely with parafilm to ensure that bacteria did not become dehydrated. The plates were

prepared in duplicate and placed in an incubator set at 37°C for 18–24 h. The colour change was then assessed visually. Any colour changes from purple to pink or colourless were recorded as positive. The lowest concentration at which colour change occurred was taken as the MIC value.

S4.2.7.4. Determination of minimum bactericidal concentrations (MBC's) of the effective plants extract

Streaks were taken from the two lowest concentrations of the plant extract plates exhibiting invisible growth (from inhibition zone of MIC plates) and subcultures onto sterile nutrient agar plates. The plates were incubated at 35°C for 24 h. then examined for bacterial growth in corresponding to plant extract concentration. MBC was taken as the concentration of plant extract that did not exhibiting any bacterial growth on the freshly inoculated agar plates.[1]

S4.2.8. Minimum biofilm inhibitory concentration (MBIC) assay

Inhibin of biofilm formation has been evaluated using 96-well microplates assay method.[2] In each well 200 µl of LB (Lauria broth) has been added. To the first well, 100 µl of the tested compound was added and two-fold dilution has been performed leaving the last two wells as control. To each well, 10 µl of the previously prepared microbial culture (0.5 McFarland standard, $5x10^5$ CFU/ml) were added. After incubating the plates at 37 ^{o}C for 24 h, the culture was gently decanted and then washed by phosphate buffered saline (PBS) buffer. The plates were left to dry for 30 min and 200 µl of crystal violet 0.1% was added to each well and left for 30 min. the excess crystal violet was decanted and washed-out three times with distilled water and left to dry for 30 min. Finally, 200 µl of 95% ethanol was added to each well and the absorbance was measured at 492 nm using ELISA reader.
